# FUS controls muscle differentiation through phase separation-mediated recruitment of the transcription factors MEF2 and ETV5

**DOI:** 10.1038/s44318-026-00874-1

**Published:** 2026-07-22

**Authors:** Gina Picchiarelli, Anne Wienand, Salim Megat, Saskia Hutten, Amr Aly, Marije Been, Isabelle Weber, Angela Bonanno, Nibha Mishra, Erin Sternburg, Pierre Cauchy, Stéphane Dieterle, Marica Catinozzi, Javier Lobo-Mancheno, Hannah Walgrave, Valérie Demais, Pierre Hener, Pascal Kessler, Laura Tzeplaeff, Annemarie Huebers, Dagmar Zeuschner, Angela Rosenbohm, Albert C Ludolph, Anne-Laurence Boutillier, Tobias Boeckers, Dorothee Dormann, Maria Demestre, Chantal Sellier, Clotilde Lagier-Tourenne, Erik Storkebaum, Luc Dupuis

**Affiliations:** 1 Université de Strasbourg, Inserm, Strasbourg Translational Neuroscience and Psychiatry, UMR-S1329, Centre de Recherches en Biomédecine, Strasbourg, France; 2 Molecular Neurobiology Laboratory, Donders Institute for Brain, Cognition and Behaviour and Faculty of Science, Radboud University, Nijmegen, Netherlands; 3 Institute of Molecular Physiology, Johannes Gutenberg-Universität (JGU), Mainz, Germany; 4 Institute of Anatomy and Cell Biology, Ulm University, Ulm, Germany; 5 Department of Neurology, The Sean M. Healey and AMG Center for ALS, Massachusetts General Hospital, Harvard Medical School, Boston, MA USA; 6 Plateforme Imagerie In Vitro, CNRS UPS-3156, NeuroPôle, Strasbourg, France; 7 Université de Strasbourg, INSERM, UMS 38, Imaging Core Facility PIC-STRA, CRBS, Strasbourg, France; 8 Université de Strasbourg, UMR 7364 CNRS, Laboratoire de Neurosciences Cognitives et Adaptatives (LNCA), Strasbourg, France; 9 Department of Neurology, Heinrich Heine-University, Düsseldorf, Germany; 10 Electron Microscopy Unit, Max Planck Institute for Molecular Biomedicine, Münster, Germany; 11 Department of Neurology, Ulm University, Ulm, Germany; 12 German Center for Neurodegenerative Diseases (DZNE) Ulm, Ulm, Germany; 13 Institute of Molecular Biology (IMB), Mainz, Germany

**Keywords:** Molecular Biology of Disease, Neuroscience, RNA Biology

## Abstract

FUS is an RNA-binding protein mutated in amyotrophic lateral sclerosis (ALS), a neurodegenerative disease characterized by progressive muscle weakness. We show in this work that a heterozygous knock-in mutation in the mouse Fus gene leads to cell-autonomous ultrastructural defects in skeletal muscle, with disruption of sarcomeres and mitochondria. Studies in mouse and Drosophila models demonstrate an evolutionarily conserved cell-autonomous function of FUS in muscle development. Mechanistically, FUS is required for the transcription of MEF2 target genes, binds to the promoter of genes bound by ETS transcription factors, in particular ETV5, and co-activates the transcription of MEF2-dependent genes with ETV5. FUS phase-separates with ETV5 and MEF2A, and stimulation of MEF2-dependent transcription by FUS is dependent upon its phase separation properties. Finally, Etv5 haploinsufficiency exacerbates muscle weakness and atrophy in Fus knock-in mice. Our findings establish a key role for FUS in skeletal muscle differentiation through its phase separation-dependent recruitment of ETV5 and MEF2, defining a novel pathway compromised in FUS-ALS.

## Synopsis

The RNA-binding protein FUS is mutated in amyotrophic lateral sclerosis (ALS), which is characterized by progressive muscle weakness. This study shows that FUS plays a direct, cell-autonomous role in skeletal muscle development and maintenance by regulating muscle gene expression through interactions with the transcription factors MEF2A and ETV5.

- Loss or mutation of FUS causes sarcomere disorganization and mitochondrial abnormalities in mouse muscle, with similar defects observed in FUS-ALS patient samples and patient-derived myotubes.
- FUS regulates muscle differentiation genes by promoting MEF2-dependent transcription and cooperating with ETV5.
- Muscle-specific restoration of normal FUS expression rescues structural defects.
- Reduced ETV5 levels accelerate muscle weakness and increase muscle atrophy in FUS mutant mice.
- The ability of FUS to undergo liquid–liquid phase separation is required for MEF2A and ETV5 recruitment and for the activation of muscle gene transcription.

## Abstract (web summary)

FUS cooperates with ETV5 to activate the transcription of MEF2-dependent genes.

## Graphical abstract

**Figure:**
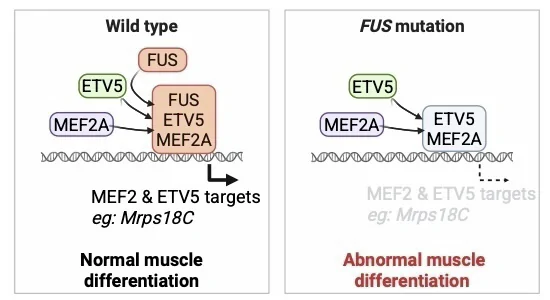

**Figure:**
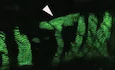

## Introduction

Amyotrophic lateral sclerosis (ALS) is the most frequent adult-onset motor neuron disease, leading to death within a few years after onset of motor symptoms (Brown and Al-Chalabi, 2017; Taylor et al, 2016; van Es et al, 2017). ALS is a genetically heterogeneous disease, with currently more than 30 associated genes. Four genes (*C9ORF72, SOD1, FUS, TARDBP*) are the major causes of familial forms of ALS. Motor symptoms of ALS are associated with degeneration of motor neurons and progressive muscle atrophy and weakness. In *SOD1*-ALS, analyses of transgenic mouse models suggested that muscle weakness is primarily a consequence of motor neuron degeneration (Shefner et al, 2023). Indeed, reducing mutant SOD1 expression in skeletal muscle of mutant SOD1 mice did not affect the disease course (Miller et al, 2006; Towne et al, 2008) and stimulating muscle mitochondrial biogenesis did not modify disease progression or survival (Da Cruz et al, 2012) in spite of delayed muscle atrophy. On the other hand, restricted expression of mutant SOD1 in skeletal muscle led to skeletal muscle damage and altered motor neurons or neuromuscular junctions (Dobrowolny et al, 2008; Martin and Wong, 2020; Wong and Martin, 2010), suggesting a possible contribution of mutant SOD1 expression in skeletal muscle to ALS-related weakness.

Compelling evidence supports the contribution of skeletal muscle defects in the pathogenesis of spinal and bulbar muscular atrophy (SBMA, a.k.a. Kennedy’s disease), another motor neurodegenerative disease caused by a polyglutamine repeat expansion in the androgen receptor (AR) (La Spada and Taylor, 2010). In a knock-in SBMA mouse model, myopathic and neurogenic skeletal muscle pathology preceded motor neuron pathology (Yu et al, 2006), and both male SBMA patients and female heterozygous carriers displayed myogenic and neurogenic myopathy (Soraru et al, 2008). In addition, muscle-specific excision of the AR121Q transgene or its peripheral knockdown in a SBMA mouse model prevented motor phenotypes, muscle pathology and motor neuronopathy, and dramatically extended survival (Cortes et al, 2014; Lieberman et al, 2014). Thus, expression of ALS-mutant genes in skeletal muscle might contribute to motor neuron disease to various extent, depending on the specific ALS gene (Picchiarelli and Dupuis, 2020; Shefner et al, 2023) and the cell autonomous impact on muscles of ALS-associated genes other than *SOD1* remains to be determined.

One of the most aggressive forms of ALS is caused by mutations in the *FUS* gene, encoding an RNA-binding protein with prominent roles in transcription, splicing and local translation (Kwiatkowski et al, 2009; Moens et al, 2025; Vance et al, 2009). *FUS-*ALS patients develop motor symptoms on average 10 to 20 years earlier than other ALS patients and show fast disease progression (Hubers et al, 2015; Millecamps et al, 2010; Waibel et al, 2010; Waibel et al, 2013). *FUS* mutations cluster in the C-terminus of the FUS protein, encoding the nuclear localization signal, which results in impaired nuclear import of the FUS protein (Dormann et al, 2010). The most severe phenotypes associated with *FUS* mutations are observed in patients carrying a nonsense or frameshift mutation, most frequently R495X, Q519Ifs*9 or G466Vfs*14, all leading to C-terminal truncation of the protein and pronounced mislocalization of FUS to the cytoplasm (Millecamps et al, 2010; Moens et al, 2025; Waibel et al, 2010; Waibel et al, 2013). We and others previously generated knock-in mouse models for *FUS*-ALS, expressing a truncated FUS protein, similar to the most severe ALS truncation mutations (Devoy et al, 2017; Scekic-Zahirovic et al, 2017; Scekic-Zahirovic et al, 2016; Zhang et al, 2020). *Fus*^∆NLS/+^ mice show cytoplasmic accumulation of FUS in the central nervous system, similar to *FUS-*ALS patients (Scekic-Zahirovic et al, 2017; Scekic-Zahirovic et al, 2021; Scekic-Zahirovic et al, 2016). These mice develop mild muscle weakness, with late onset motor neuron degeneration and cognitive phenotypes (Picchiarelli et al, 2019; Scekic-Zahirovic et al, 2017; Scekic-Zahirovic et al, 2021; Scekic-Zahirovic et al, 2016; Tzeplaeff et al, 2023). *FUS* is a ubiquitously expressed gene, including in skeletal muscle, and recent work showed abnormalities in energy metabolism pathways in *FUS*-ALS skeletal muscle (Yu et al, 2022; Zhou et al, 2022), yet it remains unknown whether these defects are cell autonomously caused by expression of mutant FUS in skeletal muscle, nor whether they are caused by loss of function of FUS in muscle or gain of toxic function of the mutant protein. Indeed, we previously showed that FUS is enriched in subsynaptic nuclei in skeletal muscles, where FUS controls transcription of acetylcholine receptor subunit genes (Picchiarelli et al, 2019), and that loss of *Fus* recapitulated these abnormalities. This suggested that expression of mutant FUS in skeletal muscle may directly contribute to neuromuscular demise in this severe form of ALS, maybe through loss of FUS function, rather than gain of toxic function.

Following up on these observations, we show here that FUS is required for muscle differentiation. Loss or mutation of *Fus* in muscle cells led to pronounced transcriptional defects, with a converging signature identifying MEF2 as a key disrupted transcription factor. Mechanistically, FUS directly binds to promoters of genes containing an ETV5-binding motif in muscle cells, and we demonstrate that FUS, MEF2A, and ETV5 cooperate to activate transcription of their target genes. As a consequence, muscle ultrastructure, including mitochondrial ultrastructure, was severely compromised in *Fus*^*ΔNLS*^ mice. Importantly, selectively reverting the *Fus*^*ΔNLS*^ mutation to wild-type in mouse skeletal muscle rescued ultrastructural defects, while muscle-selective loss of either FUS or ETV5 orthologs in *Drosophila* was sufficient to lead to muscle degeneration. Last, iPSC-derived myotubes showed similar sarcomeric and mitochondrial ultrastructural defects, suggesting that disruption of FUS-dependent transcriptional pathways contribute to muscle defects in *FUS*-ALS patients.

## Results

### Expression of mutant FUS in myocytes leads to muscle defects in mice

**Figure 1:**
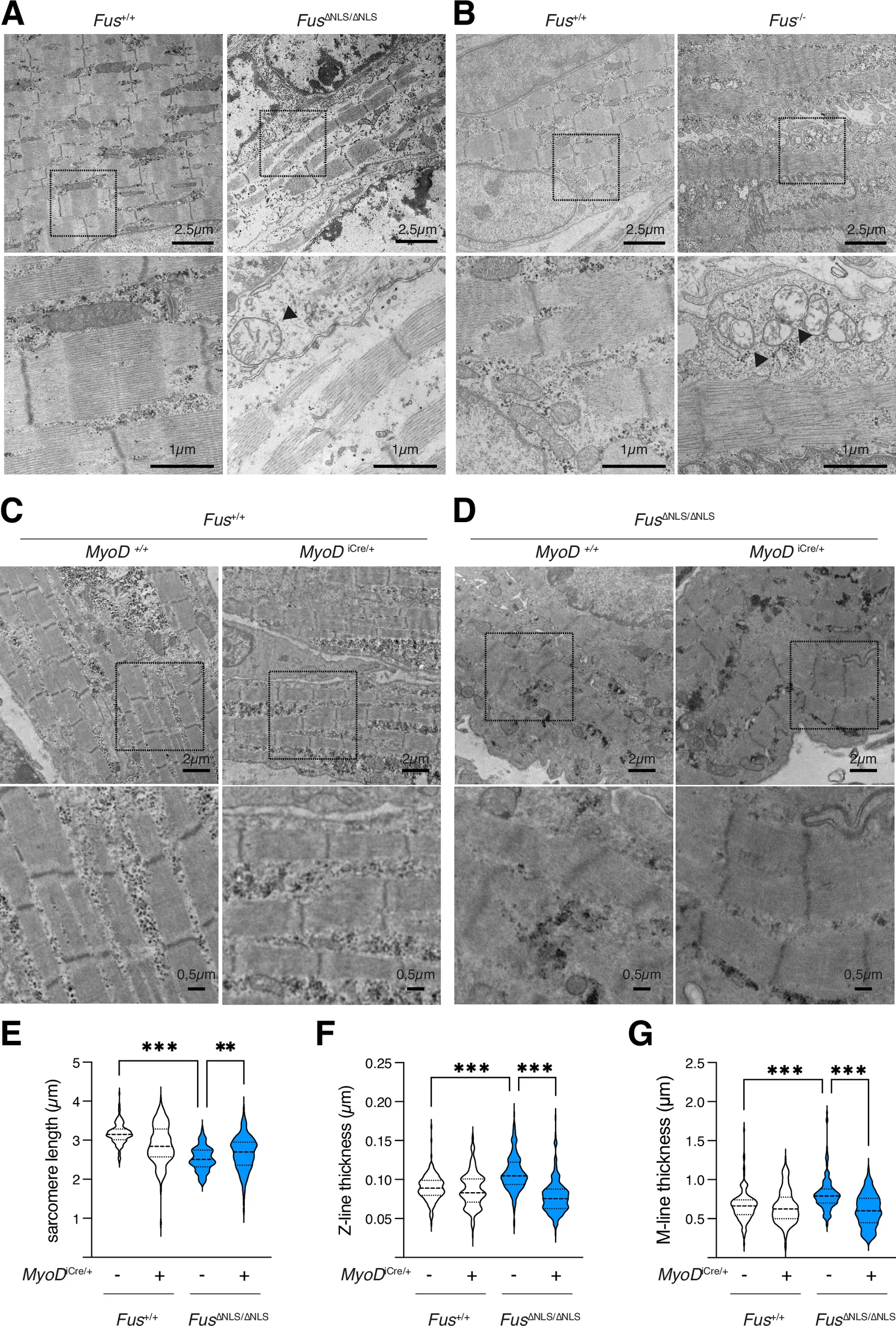
Homozygous *Fus* mutation or deletion in mice lead to muscle-autonomous ultrastructural defects. (**A**, **B**) Representative TEM images of gastrocnemius muscles of E18.5 *Fus*^*∆NLS/∆NLS*^ (**A**), *Fus*^*−/−*^ (**B**), and their respective control littermates. A higher magnification of the indicated regions (dashed boxes) is shown in the lower panels. Scale bars: 2.5 μm (upper panels) and 1 μm (lower panels). Arrowheads in lower panels indicate swollen mitochondria. (**C**,** D**) Representative TEM images in gastrocnemius muscles of E18.5 *Fus*^*+/+*^ (**C**) or *Fus*^*∆NLS/∆NLS*^ (**D**), with or without *MyoD*^iCre/+^. A higher magnification of the indicated regions (dashed boxes) is shown in the lower panels. (**E**–**G**): Quantitative analyses of ultrastructural parameters in sarcomeres of E18.5 *Fus*^*+/+*^ or *Fus*^*∆NLS/∆NLS*^, with or without MyoD^iCre/+^. *Fus*^*+/+*^: 9 animals, 697 sarcomeres analyzed; *Fus*^*∆NLS/∆NLS*^: 10 animals, 755 sarcomeres analyzed; *Fus*^*+/+*^/MyoD^iCre/+^: 4 animals, 358 sarcomeres analyzed; *Fus*^*∆NLS/∆NLS*^/MyoD^iCre/+^: 3 animals, 276 sarcomeres analyzed. (**E**) **p = 0.0039, < 0.01, ***p = 0.0002 (Kruskal–Wallis test). (**F**) ***p < 0.0001 (Kruskal–Wallis test). (**G**) ***p < 0.0001 (Kruskal–Wallis test). Violin plots show median and quartiles. Source data are available online for this figure.

**Figure EV1:**
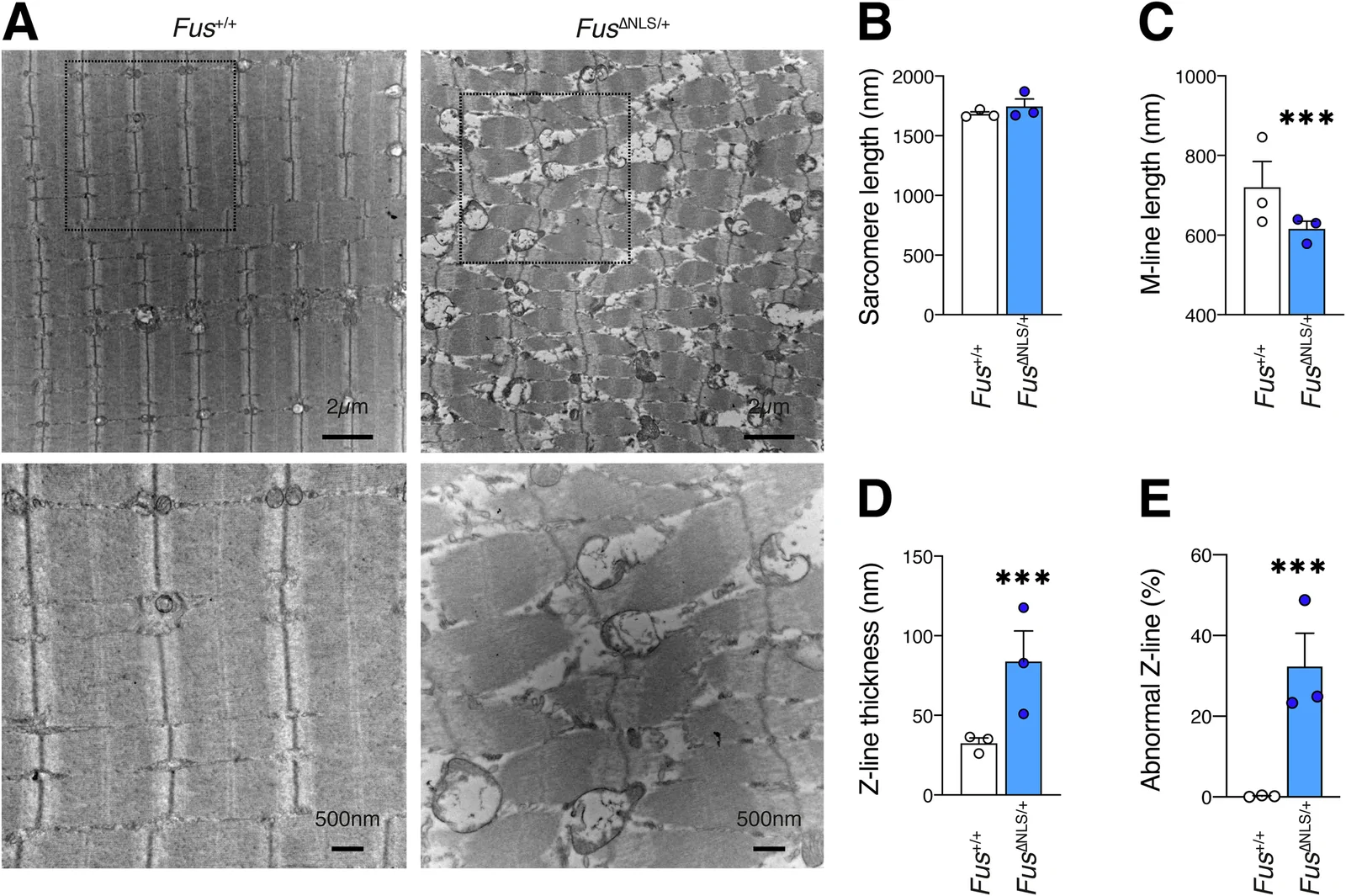
Heterozygous *Fus* mutation in mouse leads to muscle ultrastructural defects. (**A**) Representative TEM images in gastrocnemius muscle of 22-month-old *Fus*^∆NLS/+^ mice and their control littermates. A higher magnification of the indicated region (dashed box) is shown in the lower panels. (**B**–**E**) Quantitative analyses of ultrastructural parameters of sarcomeres in adult *Fus*^∆NLS/+^ mice and their control littermates. Dots represent individual animals analyzed (*n* = 3), 20 pictures were analyzed per animal. Data represent the mean ± SEM. All comparisons in (**B**–**E**) by Nested t-test. (**B**) *p* = 0.3917, (**C**) ***p = 0.004, (**D**) ***p < 0.0001, (**E**) ***p < 0.0001. Source data are available online for this figure.

**Figure EV2:**
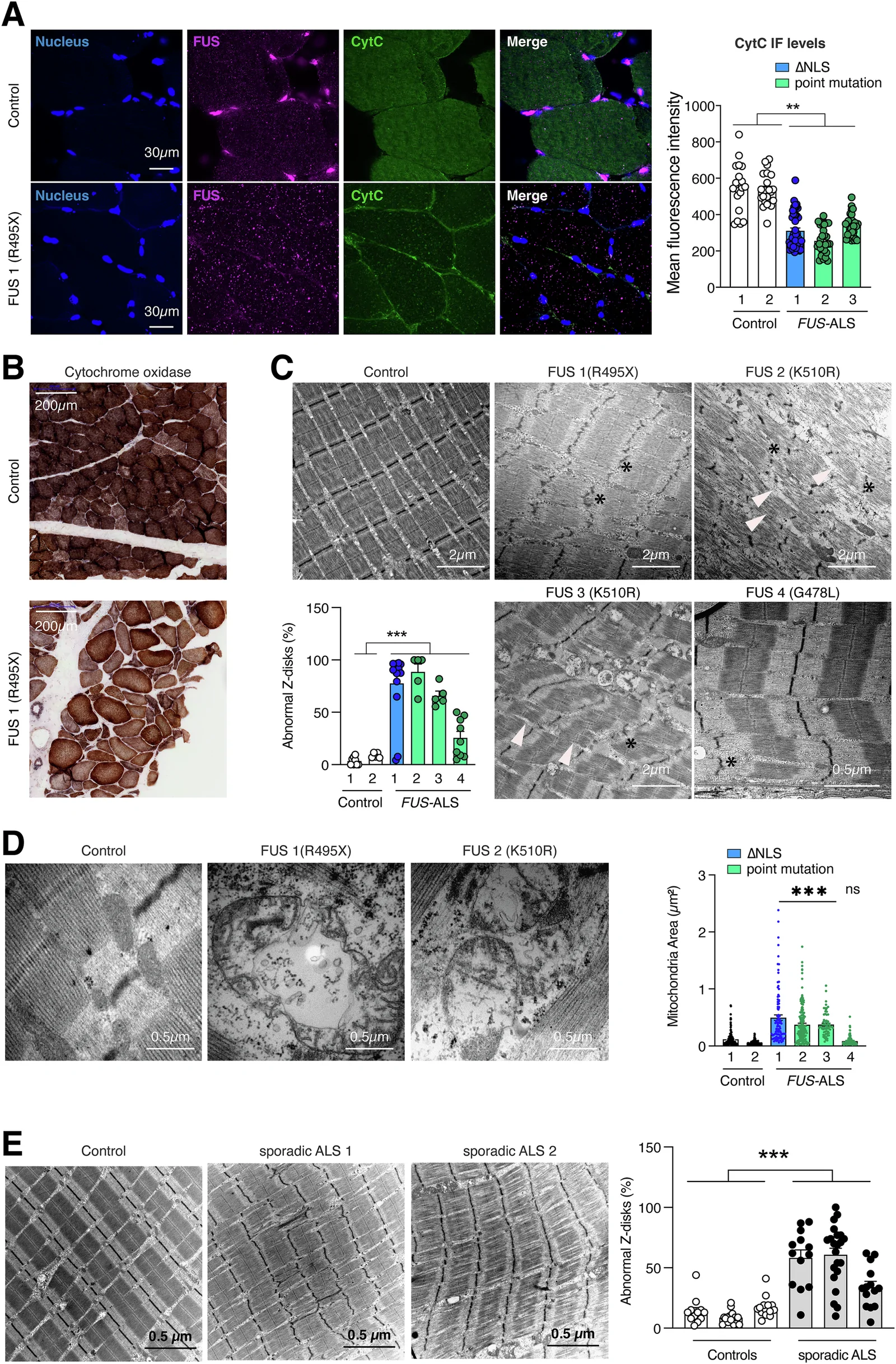
FUS mislocalization, abnormal sarcomeric ultrastructure, and mitochondrial defects in *FUS-*ALS and sporadic muscles. (**A**) Immunostaining for nucleus (DAPI, blue), FUS (magenta), and cytochrome c (CytC, green) on sections of muscle biopsies from a control individual (upper row) and FUS1 patient carrying a *FUS* truncating mutation R495X (lower row). FUS protein was mainly localized to the nucleus in the control muscle, while cytoplasmic mislocalization was evident in this *FUS-*ALS muscle. Cytochrome c staining was higher in control muscle compared to *FUS-*ALS muscle where it accumulated at the plasma membrane. Scale bar: 30 µm. Right panel shows the quantitative analysis of CytC immunoreactivity in 3 *FUS*-ALS patients. FUS1 patient carries a truncating mutation (∆NLS, blue), while FUS2 and FUS3 patients carry a K510R missense mutation (green). Data represent the mean ± SEM. Nested t-test, **p = 0.005. (**B**) Cytochrome oxidase histochemistry in control and FUS1 patient muscle. Scale bar: 200 µm. Similar results were obtained in FUS2 and FUS3 patients. (**C**) Electron micrographs of muscles in healthy control showing normal ultrastructure with aligned Z-lines, and *FUS-*ALS patients showing extensive myofibrillar disorganization (white arrowheads), including disrupted Z-lines (stars). Lower left panel shows the percentage of fragmented, curvy and/or zigzag Z-lines in controls and *FUS-*ALS patients. FUS4 patient carries a G478L mutation. Truncating mutation (∆NLS) is indicated in blue, missense mutations are indicated in green. Dots indicate the analysis of different images in the corresponding individuals. Data represent the mean ± SEM. Nested t-test; ***p < 0.0001. (**D**) Electron micrographs of muscle mitochondria in healthy control showing normal size and structure of mitochondria, and in *FUS-*ALS patients, showing fragmentation, swelling or complete destruction of mitochondria in FUS1, FUS2, and FUS3 patients, while FUS4 patient did not show altered mitochondrial ultrastructure. FUS1 patient carries a R495X truncating mutation (∆NLS, blue), while patients 2–4 carry point mutations (K510R for patients 2 and 3 and G478L for patient 4). Right panel shows quantification of mitochondrial surface area with a significant increase in mitochondrial size in *FUS-*ALS patients, except for patient FUS4 (ns: not significant). Data represent the mean ± SEM. One-way ANOVA with Tukey’s multiple comparisons test: Control versus FUS1, FUS2, FUS3: ***p < 0.0001. Scale bars: 0.5 μm. (**E**) Abnormal ultrastructure in sporadic ALS muscles. Electron micrographs of muscle in a healthy control showing normal ultrastructure with aligned Z-lines, and a sporadic ALS patient showing myofibrillar disorganization and disrupted Z-lines. Right panel shows the percentage of fragmented, curvy and/or zigzag Z-lines in 3 controls and 3 sporadic ALS patients. Dots indicate the analysis of different images in the corresponding individuals. Data represent the mean ± SEM. Nested t-test; ***p < 0.0001. Source data are available online for this figure.

To investigate the consequences of *Fus* mutation on muscle structure, we exploited two mouse models either knocked-out for the *Fus* gene (*Fus*^*−/−*^) or carrying a truncating mutation of FUS deleting the nuclear localization signal (NLS, *Fus*^*∆NLS/∆NLS*^). For both *Fus* alleles, homozygous mice die at birth of respiratory insufficiency (Scekic-Zahirovic et al, 2016) and we therefore performed transmission electron microscopy (TEM) on muscles at E18.5. We observed profound abnormalities in sarcomere ultrastructure, including faint Z-lines and disorganized myofibrils in both *Fus*^*−/−*^ and *Fus*^*∆NLS/∆NLS*^ muscles (Fig. 1A,B). These myofibrillar defects were accompanied by swollen mitochondria with loss of cristae (arrowheads in Fig. 1A,B). Heterozygous *Fus*^*∆NLS/+*^ mice carrying one mutant *Fus* allele, similar to the genetic situation in *FUS*-ALS patients, reach adulthood and show adult-onset muscle weakness and progressive motor neuron degeneration (Scekic-Zahirovic et al, 2017), although not developing full blown paralysis (Dupuis and Robertson, 2025). Notably, 22-month-old *Fus*^*∆NLS/+*^ mice also showed gastrocnemius muscle ultrastructural defects, with swollen mitochondria and sarcomeric defects, in particular in M-lines and Z-lines, similar to homozygous E18.5 animals (Fig. EV1A–E). Quantitative analysis revealed a significantly reduced M-line length and increased Z-line thickness (Fig. EV1C,D). Similar mitochondrial and ultrastructural defects were observed in muscle biopsies from two healthy control individuals, 4 *FUS-*ALS patients, and 3 sporadic ALS patients as compared to healthy control individuals, as well as in myotubes differentiated from a *FUS-*ALS patient iPSC line and its isogenic CRISPR-corrected line (Higelin et al, 2016) (Fig. EV2A–E; Appendix Table S1, Appendix Fig. S1A–C). Thus, loss or mutation of *Fus* in mouse leads to structural muscle defects, including disruption of sarcomeres and mitochondria, and similar defects are observed in human *FUS*-ALS.

The ∆NLS allele carries LoxP sites allowing CRE-dependent reversal of the mutation to wild-type *Fus* (Scekic-Zahirovic et al, 2016). To determine whether the observed muscle defects were attributable to intrinsic toxicity of mutant FUS in myocytes, we crossed *Fus*^*∆NLS*^ mice with *MyoD*^*iCre*^ mice (Kanisicak et al, 2009; Yamamoto et al, 2009), allowing targeting of myocytes as early as E10.5, as previously described and validated (Picchiarelli et al, 2019). TEM analysis of muscles of *MyoD*^*iCre/+*^*; Fus*^*∆NLS/∆NLS*^ showed a significant rescue of the decreased sarcomere length and increased Z-line and M-line thickness observed in E18.5 muscle of littermate *Fus*^*∆NLS/∆NLS*^ mice (Fig. 1C–G), demonstrating that these defects originated from expression of mutant FUS in myocytes. Thus, expression of mutant FUS in skeletal muscle is required to trigger the ultrastructural defects.

### *Drosophila caz* function in myocytes is required for maintenance of adult muscle integrity

**Figure 2:**
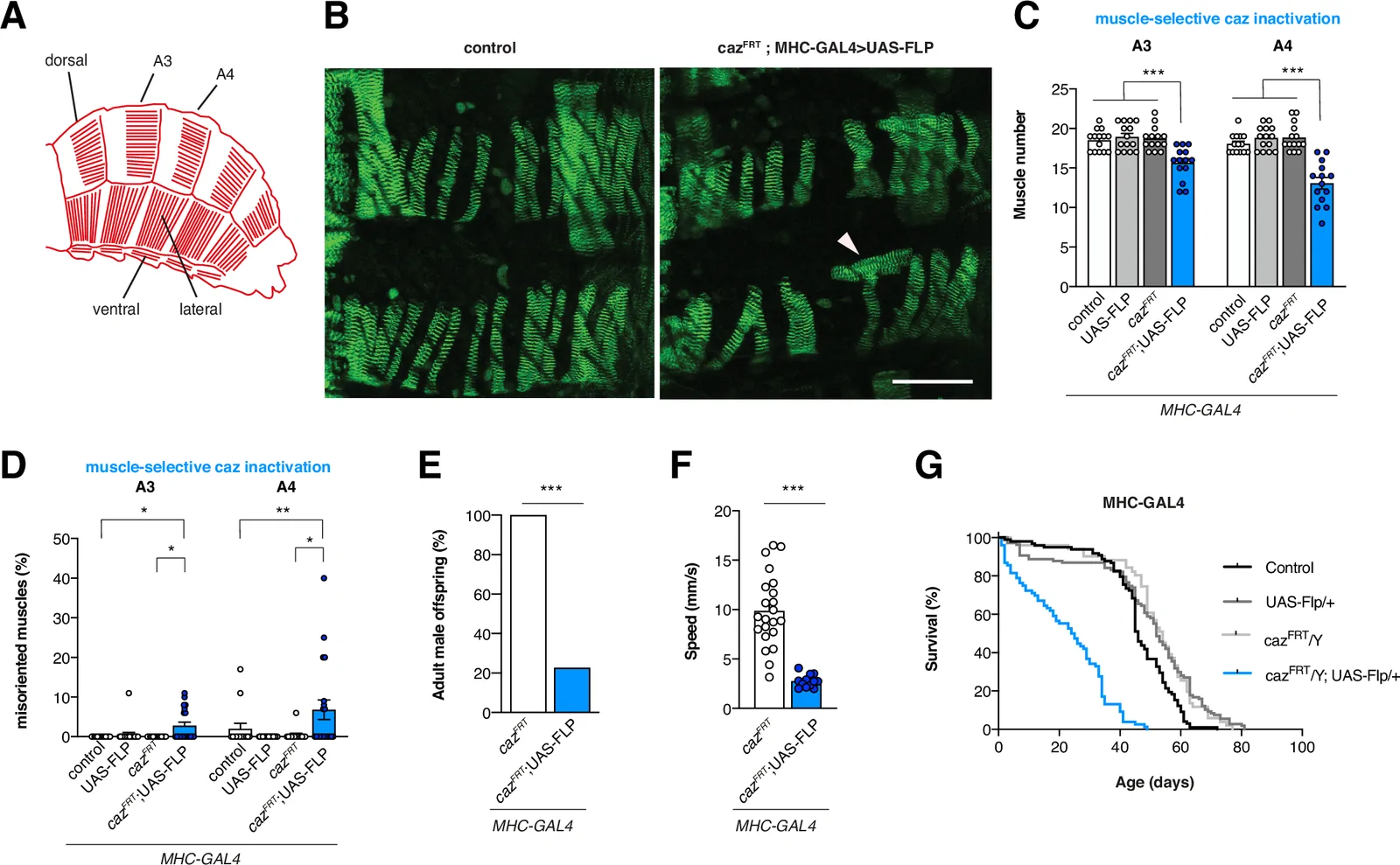
Selective *caz* inactivation in myocytes, starting after myoblast fusion, induces muscle defects, motor deficits, and reduced life span. (**A**) Schematic of adult abdominal muscle architecture in *Drosophila*. Dorsal, lateral, and ventral abdominal muscles are indicated. (**B**) Representative images of dorsal abdominal muscle defects in pharate adult flies (96 h APF: 96 h after pupa formation) in which *caz* was selectively inactivated in myocytes (*caz*^*FRT*^; *MHC-GAL4* > *UAS-FLP*) as compared to control animals. Arrowhead indicates a misoriented muscle. Scale bar: 100 µm. (**C**,** D**) Quantification of the number (**C**) and misorientation (**D**, shown as % per animal) of dorsal abdominal muscles in abdominal segments A3 and A4 of 96 h APF male pupae in which *caz* was selectively inactivated in myocytes (*caz*^*FRT*^; *MHC-GAL4* > UAS-FLP) as compared to control genotypes. (**C**) Two-way ANOVA with Tukey’s multiple comparisons test; ***p < 0.0002; *n* = 14 per genotype. Average ± SEM. (**D**) One-sample Wilcoxon signed-rank test to compare all genotypes to control, Mann–Whitney test to compare *caz*^*FRT*^ to *caz*^*FRT*^; UAS-FLP; *p = 0.0156, **p = 0.0078; *n* = 14 control, 20 UAS-FLP, 14 *caz*^*FRT*^, 20 *caz*^*FRT*^; UAS-FLP. (**E**) Adult offspring frequency of male flies in which *caz* is selectively inactivated in muscle (*caz*^*FRT*^; *MHC-GAL4* > UAS-FLP) relative to *caz*^*FRT*^; *MHC-GAL4*/+ control flies (100%). Chi-square test; ***p < 0.0001; *n* ≥ 214 F1 male flies per cross. (**F**) Climbing speed (in mm/s) in an automated negative geotaxis assay of 7-day-old *caz*^*FRT*^; *MHC-GAL4* > UAS-FLP males as compared to *caz*^*FRT*^; *MHC-GAL4*/+ control males. Mann–Whitney test; ***p < 0.0001; *n* = 22 groups of 10 *caz*^*FRT*^ flies versus 12 groups of 10 *caz*^*FRT*^; UAS-FLP flies. Average ± SEM. (**G**) Life span of *caz*^*FRT*^; *MHC-GAL4* > UAS-FLP males as compared to the relevant control genotypes. Log-rank test; *n* = 98 control, 107 UAS-FLP, 51 *caz*^*FRT*^, 76 *caz*^*FRT*^; UAS-FLP. *P* < 0.0001, UAS-FLP vs *caz*^*FRT*^; UAS-FLP. Source data are available online for this figure.

To evaluate whether the muscle cell-autonomous function of FUS is evolutionary conserved, we next turned to *Drosophila*. *Cabeza* (*caz*) is the single *Drosophila* orthologue of *FUS* and the two other FET family proteins EWSR1 and TAF15. We previously showed that *caz* is required for proper muscle development by promoting founder myoblast selection (Catinozzi et al, 2020). Here, we evaluated a possible role for Caz in later stages of muscle differentiation by selectively inactivating *caz* in differentiating myocytes, after founder myoblast selection and myoblast fusion occurred. We crossed flies that selectively express FLP recombinase in myocytes (*MHC-GAL4* > *UAS-FLP*) to conditional *caz* knock-out animals in which the *caz* gene is flanked by FRT sites *(caz*^*FRT*^*)* (Frickenhaus et al, 2015). Because both full-body *caz* knock-out and myoblast-selective *caz* inactivation resulted in a reduced number and misorientation of adult abdominal muscles in pharate adult flies (Catinozzi et al, 2020), we evaluated dorsal abdominal muscle morphology in flies in which *caz* was selectively inactivated in myocytes. Myocyte-selective *caz* inactivation resulted in a significantly reduced number of dorsal abdominal muscles, as well as occasional misorientation of these muscles in the abdominal body segments A3 and A4 (Fig. 2A–D). Consistent with defective dorsal abdominal muscle morphology and the fact that abdominal muscle function is required for adult eclosion from the pupal case, myocyte-selective *caz* inactivation resulted in a lower-than-expected number of adult offspring, indicative of an eclosion defect (Fig. 2E). To further evaluate the effect of myocyte-selective *caz* inactivation on adult muscle function, we determined the adult motor performance of ‘adult escaper’ flies using a negative geotaxis climbing assay (Niehues et al, 2015) and identified a significant motor deficit (Fig. 2F). In addition, the lifespan of myocyte-selective *caz* knock-out flies was significantly reduced (Fig. 2G). Together, these results indicate that *Drosophila caz* function in myocytes—after myoblast fusion occurred—is required for structural and functional maintenance of muscles.

### FUS modulates the MEF2-dependent transcriptome in skeletal muscle

**Figure 3:**
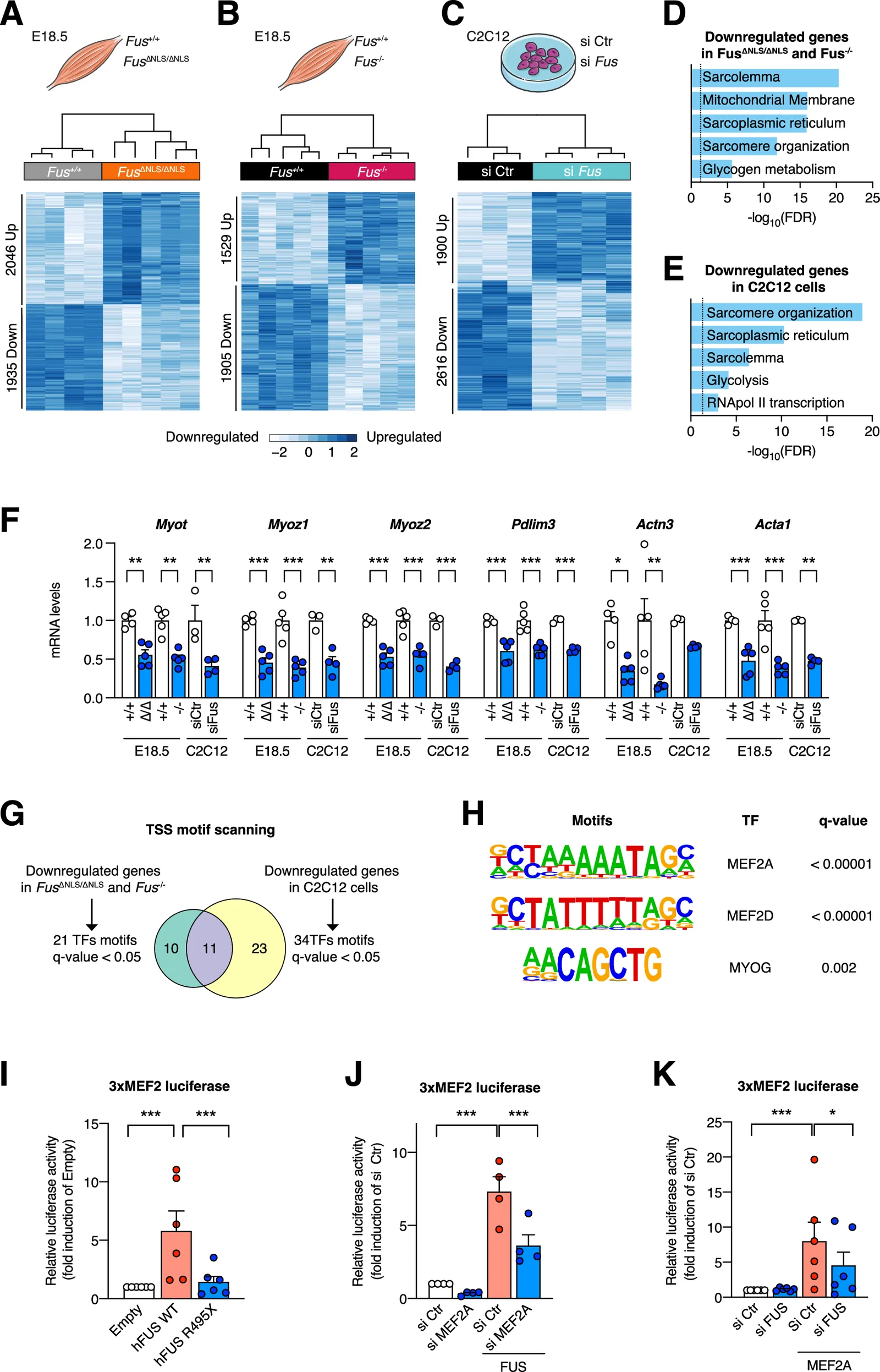
FUS modulates the MEF2-dependent transcriptome in muscle cells. (**A**–**C**) Heatmaps of differentially expressed genes in muscles from E18.5 *Fus*^∆NLS/∆NLS^ mice (**A**), *Fus*^*−*/*−*^ mice (**B**) and their respective control littermates *Fus*^+/+^, or in mouse C2C12 myoblast cells (**C**) treated with siRNA against Fus (si Fus) or control (si Ctr). (**D**,** E**) GO terms enriched in downregulated genes in *Fus*^*−*/*−*^ and *Fus*^*∆NLS/∆NLS*^ muscles (**D**) or C2C12 cells (**E**). (**F**) Normalized expression (based on FPKM from RNA-seq) of genes identified by RNA-seq to be significantly downregulated in *Fus*^*−*/*−*^ and *Fus*^*∆NLS/∆NLS*^ muscles compared to their respective wild-type controls or in C2C12 cells. Error bars represent SEM in 4–5 biological replicates. One-way ANOVA with Tukey’s multiple comparisons test. *Myot:* +/+ vs ∆/∆: *p* = 0.0058 **, +/+ vs −/− *p* = 0.0012 **, siCt vs *siFus p* = 0.0013 **; Myoz1: +/+ vs ∆/∆: *p* = 0.0003 ***, +/+ vs −/− *p* < 0.0001 ***, siCt vs *siFus p* = 0.0014 **; Myoz2: +/+ vs ∆/∆: *p* < 0.0001 ***, +/+ vs −/− *p* < 0.0001 ***, siCt vs *siFus p* < 0.0001 ***; *Pdlim3:* +/+ vs ∆/∆: *p* < 0.0001 ***, +/+ vs −/− *p* < 0.0001 ***, siCt vs *siFus p* < 0.0001 ***; *Actn3:* +/+ vs ∆/∆: *p* = 0.0414 *, +/+ vs −/− *p* = 0.0039 **, siCt vs *siFus p* = 0.6944 ns; *Acta1:* +/+ vs ∆/∆: *p* = 0.0009 ***, +/+ vs −/− *p* < 0.0001 ***, siCt vs *siFus p* = 0.0030 **. (**G**) Venn diagram showing the overlap of transcription factor (TF) motifs enriched in the promoter region of downregulated genes in *Fus*^*−*/*−*^ and *Fus*^*∆NLS/∆NLS*^ muscle and C2C12 cells with *Fus* knockdown. (**H**) Enriched motifs in transcription start sites of downregulated genes predicted to be recognized by MEF2A, MEF2D, and MYOG. (**I**) Light units (RLU) relative to empty vector in C2C12 cells 24 h after transfection with plasmids expressing hFUS WT, hFUS R495X or an empty control plasmid, and a luciferase reporter plasmid carrying three MEF2 response elements controlling luciferase expression. Nested one-way ANOVA with Tukey’s multiple comparisons test; hFUS WT vs Empty or hFUS R495X: ***p < 0.0001, *N* = 6 independent transfections. Each dot represents the mean of an individual experiment, each consisting of 4 technical replicates. (**J**,** K**) Light units relative to siCtr in C2C12 cells 24 h after transfection of 3xMEF2 luciferase plasmid and 25 nM of siCtr or siMEF2A (**J**) or siFus (**K**), along with an expression plasmid for either hFUS_WT (**J**) or MEF2A (**K**) or empty control. Nested one-way ANOVA with Tukey’s multiple comparisons test. (**J**) siCtr/empty siCtr/FUS or siCtr/FUS vs siMEF2A/FUS ***p < 0.0001. (**K**) siCtr/empty siCtr/MEF2A ***p < 0.0001; siCtr/MEF2A vs siFUS/MEF2A *p = 0.0162. Each dot represents the mean of an individual experiment (*n* = 4 for (**J**); *n* = 6 for (**K**)), each consisting of 8 (**J**) or 6 (**K**) technical replicates. Data represent the average ± SEM. Source data are available online for this figure.

We then sought to delineate underlying molecular mechanisms of FUS-mediated muscle defects, and performed RNAseq on gastrocnemius muscles of E18.5 *Fus*^*−/−*^ and *Fus*^*ΔNLS/ΔNLS*^ mice and their respective littermate controls. We also included RNAseq analysis in C2C12 cells with or without siRNA-mediated knockdown of *Fus* (Appendix Fig. S2A). In all three models, we observed extensive transcriptional alterations (Fig. 3A–C), with more than 3000 genes differentially expressed in each of the three models. Gene ontology (GO) analyses showed that downregulated genes in E18.5 *Fus*^*−/−*^ and *Fus*^*ΔNLS/ΔNLS*^ muscles and in C2C12 cells with *Fus* knock-down were related to muscle ultrastructure, including sarcolemma, sarcomere and sarcoplasmic reticulum, and mitochondrial function (Fig. 3D,E), while there was no specific GO term enrichment in the upregulated genes. Illustrating this, we observed downregulation of multiple sarcomeric protein-encoding transcripts including actin (*Acta1*) and proteins of the Z-line (e.g., *Myoz1, Myoz2, Pdlim3* or *Actn3*) in all three models (Fig. 3F).

To determine whether alterations in a similar set of transcription factors could underlie the transcriptional changes in all three models, we performed motif scanning of the transcription start site (TSS) of downregulated or upregulated genes. While there was little enrichment in specific transcription factor motifs in upregulated genes, we identified 21 transcription factor motifs enriched in downregulated genes in *Fus*^*−*/*−*^ and *Fus*^*∆NLS/∆NLS*^ mice (q < 0.05), and 34 in C2C12 cells with *Fus* knockdown. Of these transcription factor motifs, 11 were common to all three models (Fig. 3G), with a striking enrichment in motifs recognized by the MEF2 transcription factor family, in particular MEF2A and MEF2D (Fig. 3H).

To more directly determine whether FUS regulates MEF2-driven transcription in muscle cells, we transfected C2C12 cells with a reporter plasmid carrying luciferase under the control of three MEF2 response elements (3xMEF2-Luc). Co-transfection of a vector expressing wild-type FUS was able to increase the activity of the 3xMEF2-luc reporter by 2- to 10-fold (Fig. 3I**)**, while expression of ALS-linked R495X mutant FUS, leading to its cytoplasmic retention (Appendix Fig. S3A–C), did not increase 3xMEF2-luc reporter activity. The FUS-induced increase was, at least in part, dependent upon endogenous MEF2A expression as FUS-driven 3xMEF2-luc reporter activity was blunted by simultaneous knockdown of MEF2A using siRNA (Fig. 3J; Appendix Fig. S2B). Consistently, MEF2A overexpression increased 3xMEF2-luc activity, and this increase was reduced by knockdown of endogenous FUS (Fig. 3K; Appendix Fig. S2A). Thus, FUS modulates MEF2-dependent transcription, which could underlie the observed muscle defects.

### FUS binds to and transactivates genes regulated by ETS transcription factors of the PEA3 family

**Figure 4:**
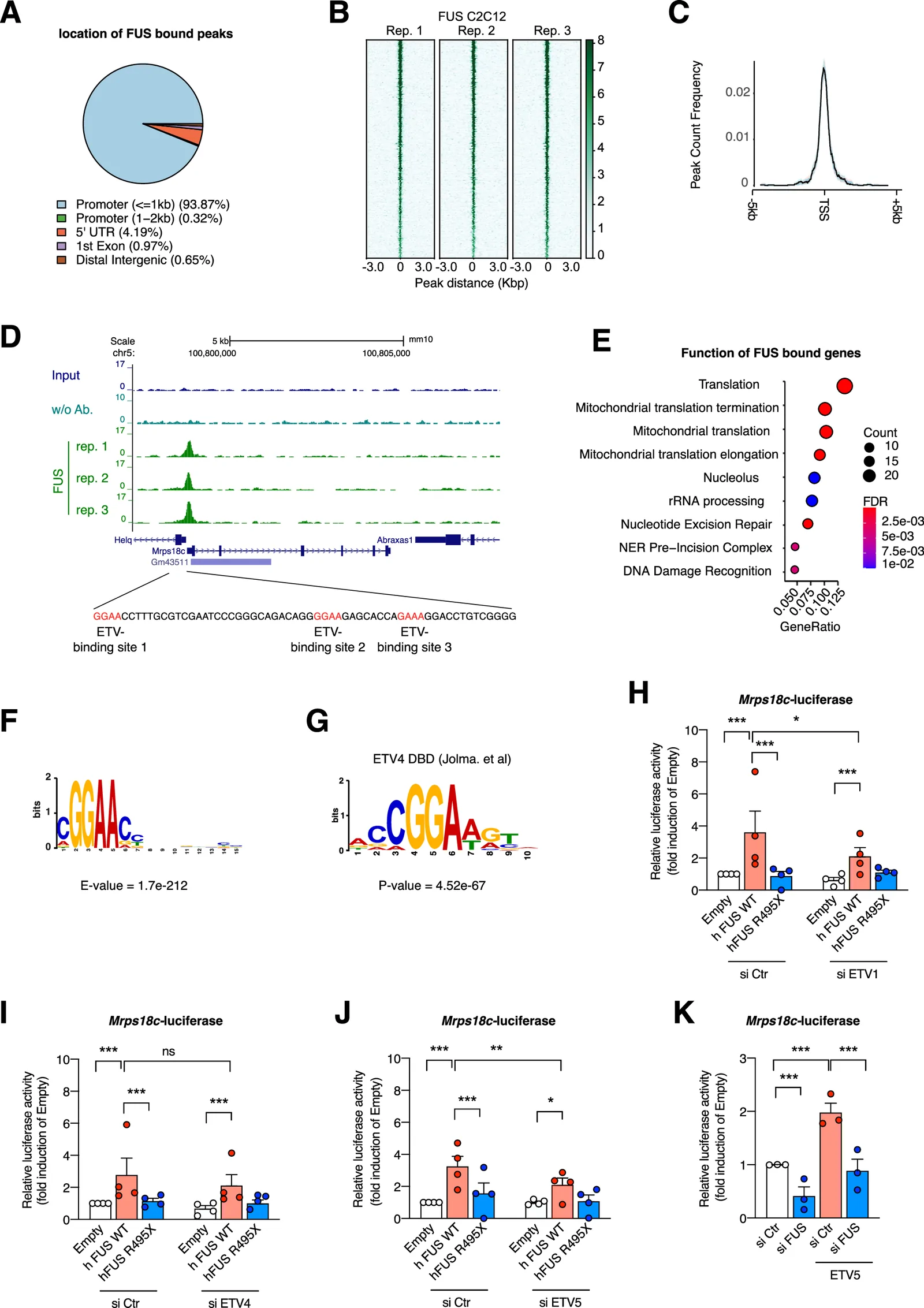
FUS is a transcriptional co-activator of PEA3 transcription factors. (**A**) Pie chart showing the distribution of FUS binding sites identified by ChIP-seq analysis in C2C12 cells. FUS binds mostly to promoters, close to the transcription start sites (TSS). (**B**,** C**) Mean profiles established for FUS binding at TSS using SeqMiner in 3 independent replicates. (**D**) Genome browser visualization of FUS binding on the promoter region of the *Mrps18c* and *Helq* genes. (**E**) Gene Ontology analysis of genes with FUS binding sites identified by ChIP-seq in C2C12 cells. (**F**,** G**) MEME motif enriched in ChIP-seq peaks bound by FUS (**F**) and the predicted motif bound by PEA3 transcription factor ETV4 (**G**). (**H**–**J**) Light units relative to empty control plasmid in C2C12 cells 24 h after transfection of an *Mrps18c-*luciferase plasmid and an expression plasmid for either hFUS WT, hFUS R495X or empty control. Cells were transfected with 25 nM of siCtr or siETV1 (**H**), siETV4 (**I**), and siETV5 (**J**). Nested one-way ANOVA with Tukey’s multiple comparisons test; ***p < 0.001, **p < 0.01, *p < 0.05. Each dot represents the mean of an individual experiment (*n* = 3), each consisting of 4 technical replicates. Data represent the mean ± SEM. (**H**) siCtr/empty vs siCtr/hFUS WT: *** *p* < 0.0001, siCtr/hFUS WT vs siCtr/hFUS R495X ***p < 0.0001, siCtr/hFUS WT vs siETV1/hFUS WT *p = 0.0153, siETV1/empty vs siETV1/hFUS WT ***p < 0.0001. (**I**) siCtr/empty vs siCtr/hFUS WT: *** *p* < 0.0001, siCtr/hFUS WT vs siCtr/hFUS R495X ***p = 0.0002, siCtr/hFUS WT vs siETV4/hFUS WT ns *p* = 0.4489, siETV4/empty vs siETV4/hFUS WT ***p < 0.0001. (**J**) siCtr/empty vs siCtr/hFUS WT: *** *p* < 0.0001, siCtr/hFUS WT vs siCtr/hFUS R495X ***p < 0.0001, siCtr/hFUS WT vs siETV5/hFUS WT ** *p* = 0.0092, siETV5/empty vs siETV5/hFUS WT *p = 0.0185. (**K**) Light units relative to si Ctr in C2C12 cells 24 h after transfection of *Mrps18c-*luciferase plasmid and 25 nM of si Ctr or si FUS and an expression plasmid for either ETV5 or empty control. Nested one-way ANOVA with Tukey’s multiple comparisons test; ***p < 0.001. Each dot represents the mean of an individual experiment (*n* = 3), each consisting of 4 technical replicates. Data represent the mean ± SEM. siCtr/empty vs siFUS/empty: ***p = 0.0029, siCtr/empty vs siCtr/ETV5 ***p < 0.0001. siCtr/ETV5 vs si*Fus*/ETV5 ***p < 0.0001. Source data are available online for this figure.

To determine whether the observed transcriptional alterations could be directly driven by FUS itself, we performed FUS ChIP-seq in C2C12 cells. We observed that FUS binds to relatively few genomic binding sites, with 294 significant peaks identified in C2C12 cells (Fig. 4A). Peaks identified in FUS ChIP-seq but not in control IgG ChIP-seq were mostly located around transcription start sites of genes (Fig. 4B,C). A typical FUS binding site is shown in Fig. 4D close to the transcription start sites of the *Mrps18c* and *Helq* genes. FUS binding sites were enriched in genes involved in mitochondrial function, in particular mitochondrial translation (Fig. 4E). Motif analysis of FUS binding peaks (Fig. 4F) revealed a striking enrichment in a motif that is the predicted binding site of ETS transcription factors of the PEA3 family (ETV1, 4, and 5) (Fig. 4G), as illustrated by the presence of 3 such PEA3-binding motifs in the *Mrps18c* promoter (Fig. 4D).

To determine whether FUS could co-activate genes with ETS transcription factors, we generated a luciferase reporter under the control of the *Mrps18c* promoter that includes three ETS transcription factor motifs at the FUS binding site (Fig. 4D). Wild-type, but not R495X, FUS overexpression induced *Mrps18c* promoter activity (Fig. 4H–J). Notably, all three PEA3 ETS transcription factors were expressed at significant levels in embryonic muscle and C2C12 cells (Appendix Fig. S4), suggesting that FUS action on its identified genomic binding sites could depend on either of the three PEA3 ETS transcription factors. Consistent with this notion, FUS mediated activation of *Mrps18c* promoter was reduced by simultaneous knock-down of *Etv1* or *Etv5*, but not *Etv4* (Fig. 4H–J). Conversely, ETV5 overexpression increased *Mrps18c* promoter activity, and this was prevented by simultaneous FUS knock-down (Fig. 4K). Together, these data suggest that FUS and PEA3 transcription factors cooperate to stimulate transcription of target genes.

### ETS transcription factors of the PEA3 family are required for muscle structure and reduced *Etv5* levels precipitates *Fus-*mediated muscle weakness and atrophy in mice

**Figure 5:**
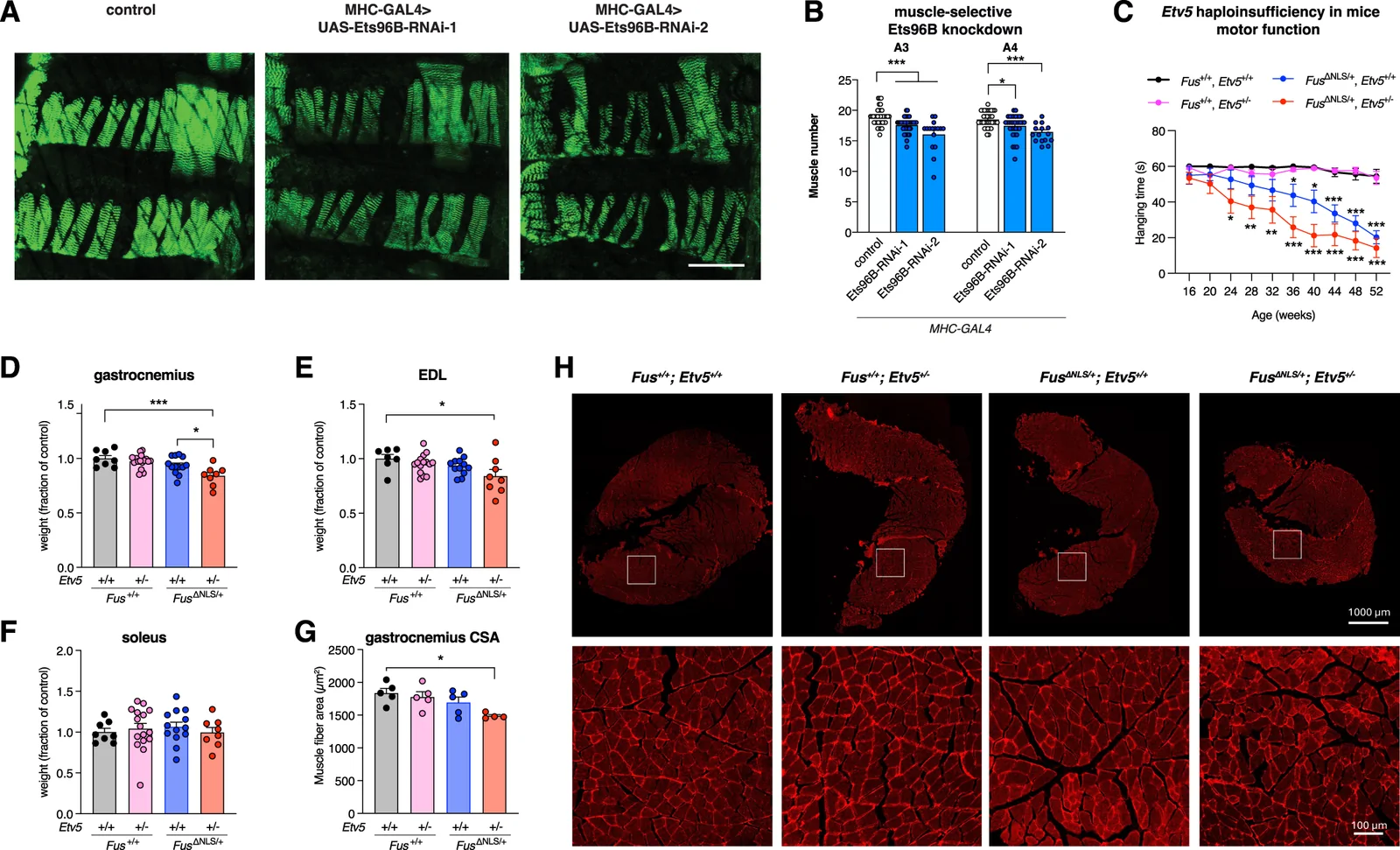
Reduced expression of *ETV5* orthologues induces muscle defects in *Drosophila* and precipitates muscle deficits of *Fus*^ΔNLS/+^ mice. (**A**) Representative images of dorsal abdominal muscles in pharate adults (96 h APF) in which Ets96B was selectively knocked-down in myocytes (MHC-GAL4 > UAS-Ets96B-RNAi) using two independent RNAi lines as compared to control. Scale bar: 100 µm. (**B**) Quantification of the number of dorsal abdominal muscles in abdominal segments A3 and A4 of 96 h APF male pupae in which Ets96B was selectively knocked down in myocytes (as compared to control). Kruskal–Wallis test followed by Dunn’s multiple comparisons test. Data represent the mean ± SEM. A3: control vs Ets96B-RNAi-1: ***p = 0.0007, control vs Ets96B-RNAi-2: ***p < 0.0001. A4: control vs Ets96B-RNAi-1: *p = 0.047, control vs Ets96B-RNAi-2: ***p < 0.0001. *n* = 37 control, 37 Ets96B-RNAi-1, and 16 Ets96B-RNAi-2. (**C**) Hanging time in the inverted grid test of male *Fus*^ΔNLS/+^ x *Etv5*^+/*−*^ mice from 16 to 52 weeks of age. *n* = 9 or 10 mice per genotype. Data represent the mean ± SEM. One-sample t-test with theoretical mean 60 s. 24 weeks, *Fus*^ΔNLS/+^ x *Etv5*^+/*−*^ : *p = 0.0169; 28 weeks, *Fus*^ΔNLS/+^ x *Etv5*^+/*−*^ : **p = 0.0055; 32 weeks, *Fus*^ΔNLS/+^ x *Etv5*^+/*−*^ : **p = 0.01; 36 weeks, *Fus*^ΔNLS/+^ x *Etv5*^+/+^ : *p = 0.0274, *Fus*^ΔNLS/+^ x *Etv5*^+/*−*^ : ***p = 0.0003; 40 weeks, *Fus*^ΔNLS/+^ x *Etv5*^+/+^ : *p = 0.0119, *Fus*^ΔNLS/+^ x *Etv5*^+/*−*^ : ***p = 0.0002; 44 weeks, *Fus*^ΔNLS/+^ x *Etv5*^+/+^ : ***p = 0.0004, *Fus*^ΔNLS/+^ x *Etv5*^+/*−*^ : ***p = 0.0001; 48 weeks, *Fus*^ΔNLS/+^ x *Etv5*^+/+^ : ***p < 0.0001, *Fus*^ΔNLS/+^ x *Etv5*^+/*−*^ : ***p < 0.0001; 52 weeks, *Fus*^ΔNLS/+^ x *Etv5*^+/+^ : ***p < 0.0001, *Fus*^ΔNLS/+^ x *Etv5*^+/*−*^ : ***p < 0.0001. (**D**–**F**) Weight of the gastrocnemius (**D**), extensor digitorum longus (EDL) (**E**) and soleus (**F**) muscles of *Fus*^*ΔNLS/+*^ x *Etv5*^*+/−*^ mice at 96 weeks of age, shown as percentage (%) of *Fus*^*+/+*^; *Etv5*^*+/+*^. *n* = 7–16 animals per genotype. Data represent the mean ± SEM. One-way ANOVA with Sidak’s multiple comparisons test. (**D**) *Fus*^+/+^ x *Etv5*^+/+^ vs *Fus*^ΔNLS/+^ x *Etv5*^+/*−*^: ***p = 0.0005, *Fus*^∆NLS/+^ x *Etv5*^+/+^ vs *Fus*^ΔNLS/+^ x *Etv5*^+/*−*^: *p = 0.0250; (**E**) *Fus*^+/+^ x *Etv5*^+/+^ vs *Fus*^ΔNLS/+^ x *Etv5*^+/*−*^: *p = 0.0255. (**F**) *p* = 0.8550 (ns). (**G**) Average muscle fiber area size in the gastrocnemius muscle of 96-week-old *Fus*^*ΔNLS/+*^ x *Etv5*^*+/−*^ mice. *n* = 4–5 animals per genotype. Data represent the mean ± SEM. One-way ANOVA with Sidak’s multiple comparisons test. *Fus*^ΔNLS/+^ x *Etv5*^+/*−*^ vs *Fus*^ΔNLS/+^ x *Etv5*^+/+^ : *p = 0.0197. (**H**) Representative images of dystrophin-stained gastrocnemius muscle of the indicated genotypes. Scale bar: 1000 µm (upper row), 100 µm (lower row). Source data are available online for this figure.

**Figure EV3:**
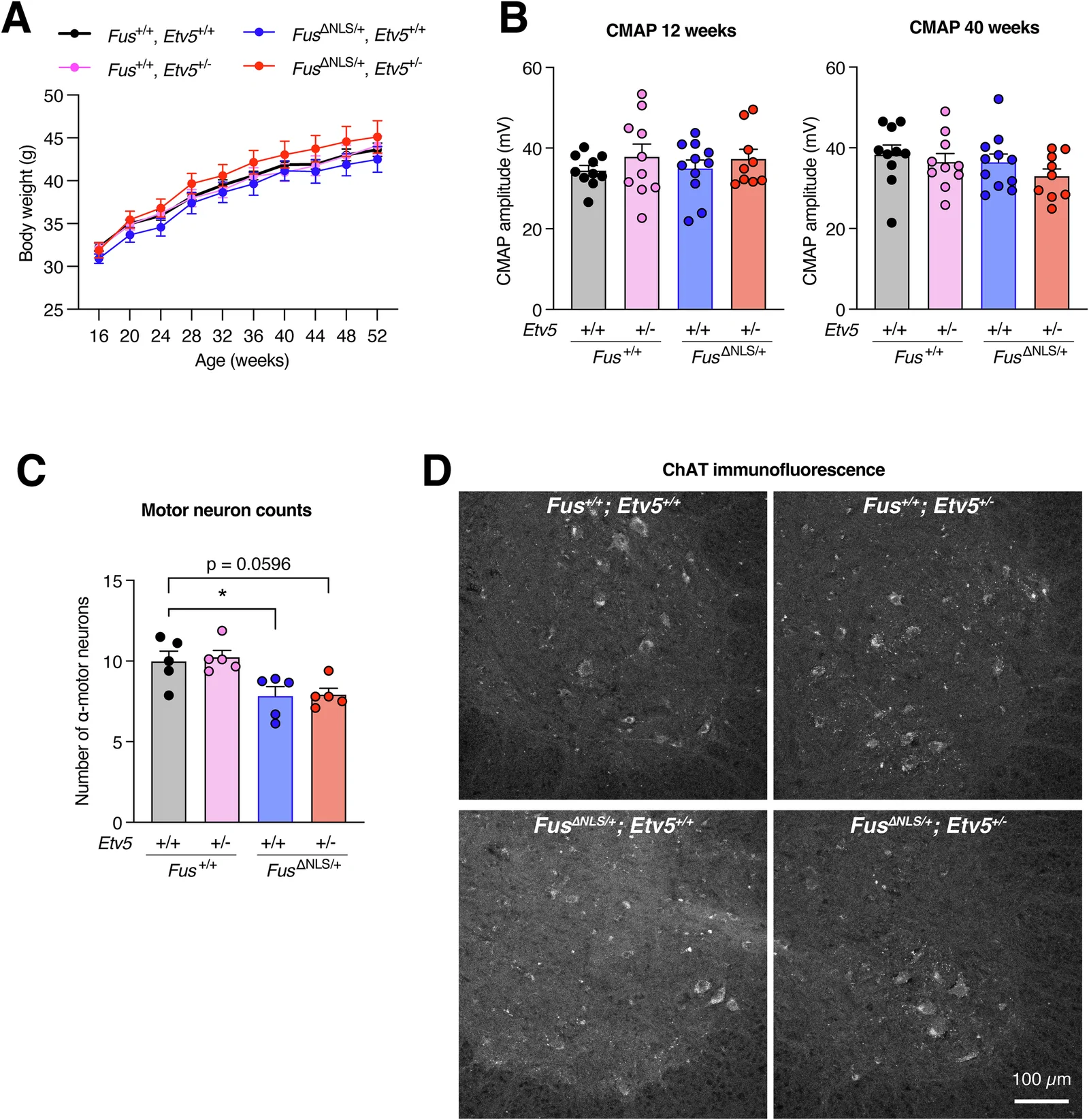
Additional characterization of *Etv5* haploinsufficiency in *Fus*^*∆NLS/+*^ mice. (**A**) Body weight of *Fus*^*+/+*^; *Etv5*^*+/+*^, *Fus*^*+/+*^; *Etv5*^*+/−*^, *Fus*^*ΔNLS/+*^; *Etv5*^*+/+*^ and *Fus*^*ΔNLS/+*^; *Etv5*^*+/−*^ mice determined every 4 weeks from 16 to 52 weeks of age. *n* = 9–10 mice per genotype. Data represent the mean ± SEM. *p* = 0.4799 for genotype effect by mixed effect analysis with Tukey’s multiple comparisons test. (**B**) Compound muscle action potential (CMAP) amplitude in the gastrocnemius muscle as determined by electromyography (EMG) after stimulation of the sciatic nerve of *Fus*^*ΔNLS/+*^ x *Etv5*^*+/−*^ mice at 12 (left) or 40 (right) weeks of age. *n* = 9–11 mice per genotype. Data represent the mean ± SEM. *p* = 0.6677 (12 weeks) and *p* = 0.3952 (40 weeks) by one-way ANOVA. (**C**) Average number of α-motor neurons (defined as ChAT-positive motor neurons with an area size of ≥300 μm^2^) per section in the ventral horn of the lumbar spinal cord. *n* = 5 animals per genotype. Data represent the mean ± SEM. One-way ANOVA with Sidak’s multiple comparisons test. *Fus*^+/+^ x *Etv5*^+/+^ vs *Fus*^ΔNLS/+^ x *Etv5*^+/+^ * *p* = 0.0418; *Fus*^+/+^ x *Etv5*^+/+^ vs *Fus*^ΔNLS/+^ x *Etv5*^+/*−*^: *p* = 0.0596. (**D**) Representative ChAT immunohistochemistry images in spinal cord of the indicated genotypes. Scale bar: 100 µm. Source data are available online for this figure.

Since FUS co-activates transcriptional activity of the PEA3 family transcription factors, we reasoned that loss of PEA3 family genes may phenocopy loss of FUS function. In *Drosophila*, *Ets96B* is the single ortholog of the mammalian *ETV1*, *ETV4*, and *ETV5* genes. Consistent with our hypothesis, myocyte-selective knock-down of *Ets96B* resulted in a reduced number of dorsal abdominal muscles for two independent transgenic RNAi lines (Fig. 5A,B), a very similar phenotype as that induced by myocyte-selective *caz* inactivation (Fig. 2). To extend this analogy in a mammalian system, we crossed *Etv5* heterozygous knock-out mice (Chen et al, 2005) to *Fus*^∆NLS/+^ mice. *Etv5*^*+/−*^ mice did not show muscle weakness as evaluated using inverted grid test (Scekic-Zahirovic et al, 2017) (Fig. 5C). However, heterozygosity for *Etv5* triggered earlier onset and more severe muscle weakness in *Fus*^∆NLS/+^ mice (Fig. 5C), as well as decreased muscle mass in gastrocnemius (Fig. 5D) and EDL (Fig. 5E), but not soleus muscle (Fig. 5F). This was accompanied by decreased cross sectional area of muscle fibers in gastrocnemius muscle of *Fus*^∆NLS/+^ mice with *Etv5* haploinsufficiency (Fig. 5G, H). Interestingly, *Etv5* haploinsufficiency did not affect body weight (Fig. EV3A) or compound muscle action potential amplitude at 12 and 40 weeks of age (Fig. EV3B) and did not exacerbate late-onset motor neuron loss in *Fus*^∆NLS/+^ mice (Fig. EV3C,D). In summary, FUS and ETV5 are synergistically required to maintain muscle function through a mechanism conserved across species.

### FUS co-activates MEF2- and ETV5-dependent transcription

**Figure 6:**
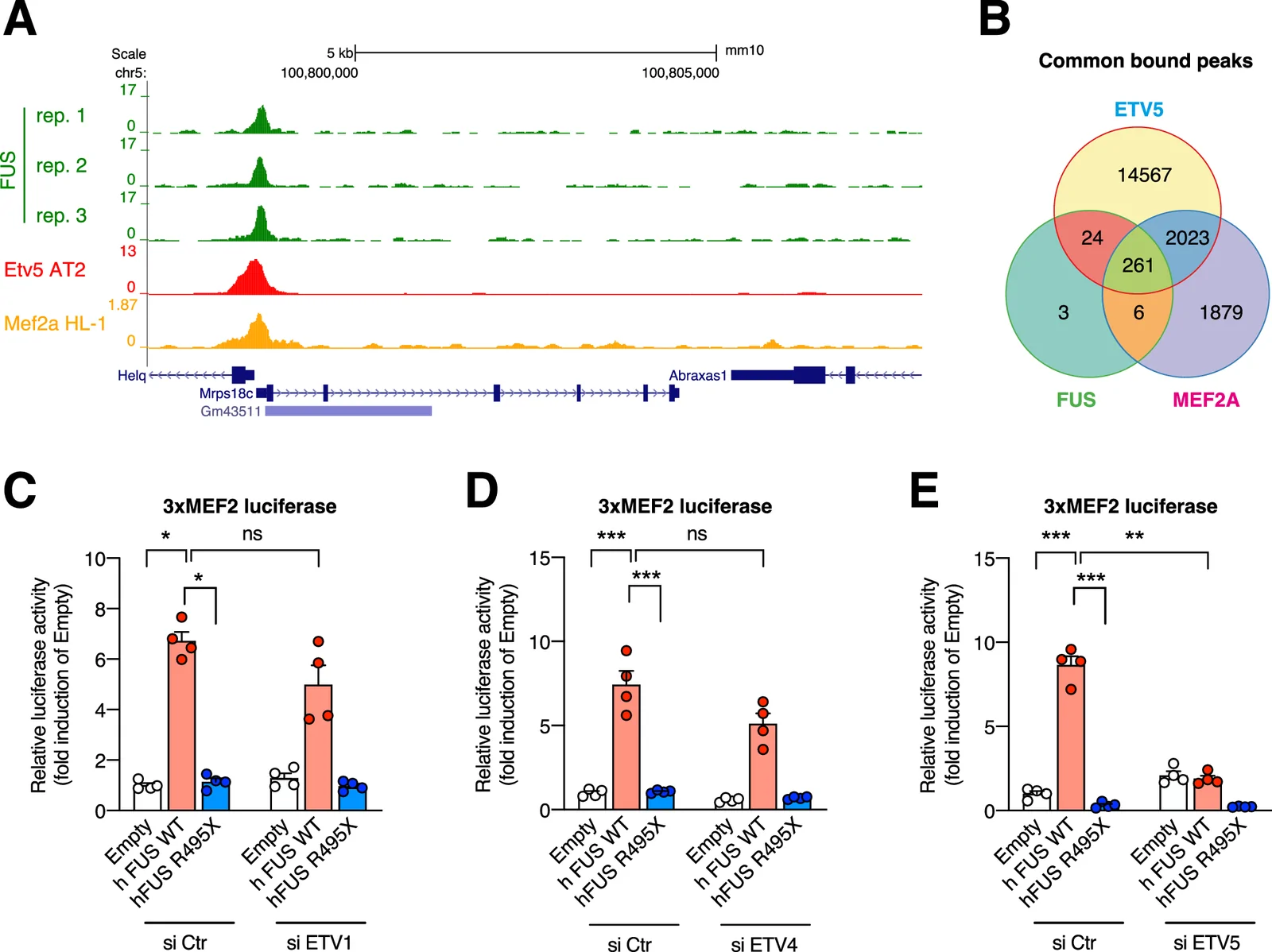
ETV5 is required for FUS-mediated co-activation of MEF2A target genes. (**A**) Genome browser snapshot of MEF2A, ETV5 and FUS ChIP-seq binding sites at the *Mrps18c/Helq* locus. (**B**) Venn diagram showing the overlap between FUS, MEF2A and ETV5 binding sites. Hypergeometric *p*-values: FUS vs ETV5: 3.66 × 10^*−*248^; FUS vs MEF2A: <5 × 10^*−*324^; ETV5 vs MEF2A: <5 × 10^*−*324^. (**C**–**E**) Light units relative to Empty vector + si Ctr in C2C12 cells 24 h after transfection of a *3xMEF2* luciferase plasmid and an expression plasmid for either hFUS WT, hFUS R495X or empty control. Cells were transfected with 25 nM si Ctr or siETV1 (**C**), siETV4 (**D**), and siETV5 (**E**). ETV5, but not ETV1 or ETV4, is required for co-activation of the MEF2 reporter by FUS. Nested one-way ANOVA with Tukey’s multiple comparisons test. (**C**) siCtr/empty vs siCtr/hFUS WT: *p = 0.0112, siCtr/hFUS WT vs siCtr/hFUS R495X *p = 0.0132, siCtr/hFUS WT vs siETV1/hFUS WT ns *p* = 0.7841. (**D**) siCtr/empty vs siCtr/hFUS WT: ***p = 0.0005, siCtr/hFUS WT vs siCtr/hFUS R495X ***p = 0.0005, siCtr/hFUS WT vs siETV4/hFUS WT ns *p* = 0.3053. (**E**) siCtr/empty vs siCtr/hFUS WT: ***p = 0.0006, siCtr/hFUS WT vs siCtr/hFUS R495X ***p = 0.0003, siCtr/hFUS WT vs siETV5/hFUS WT **p = 0.0019. Each dot represents the mean of an individual experiment (*n* = 4), each consisting of 4 technical replicates. Data represent the mean ± SEM. Source data are available online for this figure.

Our previous results showed a direct functional connection between FUS and PEA3 ETS transcription factors in muscle, and that *Fus* mutation hampers MEF2-dependent transcription. Notably, previous studies had suggested functional relationships between MEF2 and ETS transcription factors. Indeed, MEF2 co-activates the activity of ETV transcription factors during myogenic differentiation (Taylor et al, 1997), and MEF2 and ETS transcription factors share genomic binding sites (Kheradpour and Kellis, 2014). Indeed, analysis of ChIP-seq datasets demonstrated that MEF2A and ETV5 binding sites were present at locations of FUS genomic binding (Fig. 6A) with a significant overlap between MEF2A, ETV5, and FUS binding sites (Fig. 6B). While only a small proportion of ETV5 and MEF2A peaks were also bound by FUS, almost all FUS binding sites were also bound by ETV5 and/or MEF2A (Fig. 6B; Appendix Fig. S5A–C). Consistently, gene set enrichment analysis (GSEA) showed significant enrichment between FUS peaks and genes downregulated following siRNA-mediated knockdown of *Fus* in C2C12 cells and E18.5 *Fus*^*ΔNLS/ΔNLS*^ muscles, and between MEF2A binding and genes downregulated in all three datasets (Appendix Fig. S6A–C), suggesting a direct functional co-activation of MEF2A target genes by FUS and ETV5. Indeed, knockdown of ETV5, but not of ETV1 or ETV4, blunted activation of MEF2 transcriptional activity by FUS 24 h after transfection of a MEF2-dependent luciferase reporter (Fig. 6C–E). These data indicate that ETV5 is required for FUS-mediated co-activation of MEF2A target genes.

### FUS phase separation promotes recruitment of MEF2A and ETV5 and is required for transactivation of target genes

**Figure 7:**
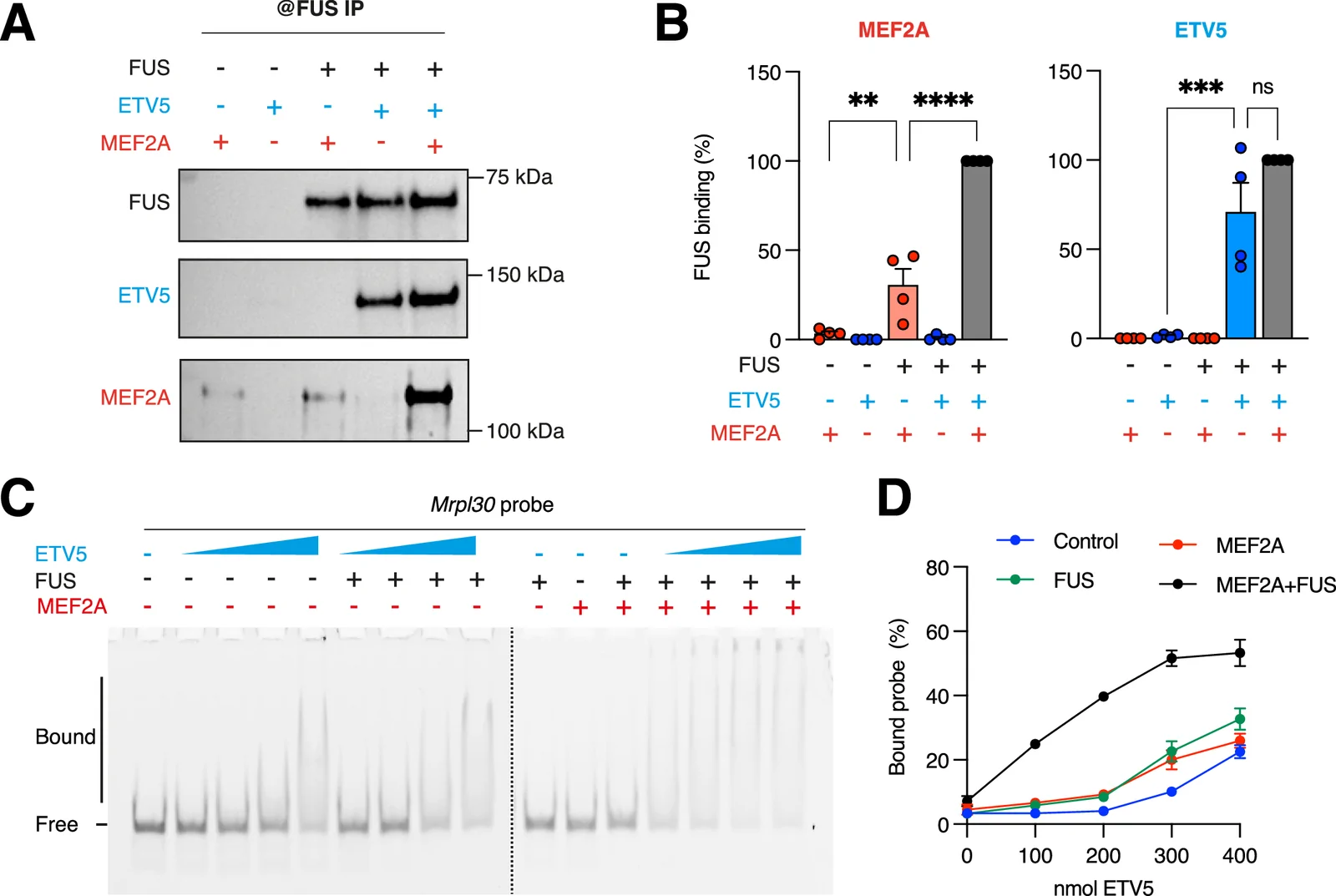
FUS binds to MEF2A and ETV5 and allows cooperative binding on DNA target sites. (**A**,** B**) Recombinant FUS protein was incubated in presence of ETV5 or/and MEF2A recombinant proteins. After immunoprecipitation using FUS antibody then pulled out with A/G-coupled magnetic beads, immunoprecipitated proteins were examined by western blot using FUS, ETV5 and MEF2A antibody. The band intensities of co-immunoprecipitated MEF2A and ETV5 were quantified using iBright Analysis Software (**B**). Error bars represent SEM of four independent experiments. Two-way ANOVA followed by Tukey post-hoc test with experiment and conditions as two factors. Panel MEF2A: **p = 0.0053 (MEF2A vs MEF2A + FUS), ****p < 0.0001 (MEF2A + FUS vs MEF2A + FUS + ETV5). Panel ETV5: ***p = 0.0002 (ETV5 vs ETV5 + FUS), ns *p* = 0.0918 (ETV5 + FUS vs MEF2A + FUS + ETV5). (**C**,** D**) Cooperative binding assay of FUS, ETV5, MEF2A recombinant proteins to FAM-labeled *Mrpl30* promoter-derived DNA probe. EMSA experiments performed by using increasing concentrations of ETV5 (60, 120, 180 and 240 nM) and constant concentration of FUS (300 nM) and/or MEF2A (50 nM). Panel (**D**) shows the percentage of *Mrpl30* probe bound across 3 independent experiments. The intensity of the signals of the protein-*Mrpl30* probe complex was quantified using the iBright Analysis Software. Error bars represent SEM of three independent experiments. Source data are available online for this figure.

To further examine whether this functional interaction could involve direct protein interaction, we performed co-immunoprecipitation of FUS in presence of ETV5 and/or MEF2A using recombinant proteins. Both MEF2A and ETV5 proteins interacted with FUS alone, and MEF2A/FUS interaction was increased by addition of ETV5 (Fig. 7A,B). Electrophoretic mobility shift assays (EMSA) using a *Mrpl30*-derived DNA probe encompassing the FUS binding motif at this gene showed that addition of ETV5 resulted in a diffuse high-molecular-weight smear, consistent with higher-order assembly of ETV5 and the probe, and this smear appeared at lower ETV5 concentrations in the presence of FUS and MEF2A (Fig. 7C,D). Similar results were obtained for ETV1, but not ETV4 (Appendix Fig. S7A,B). Importantly, mutating the ETV5 binding motifs in the probe abolished binding (Appendix Fig. S7C,D).

**Figure 8:**
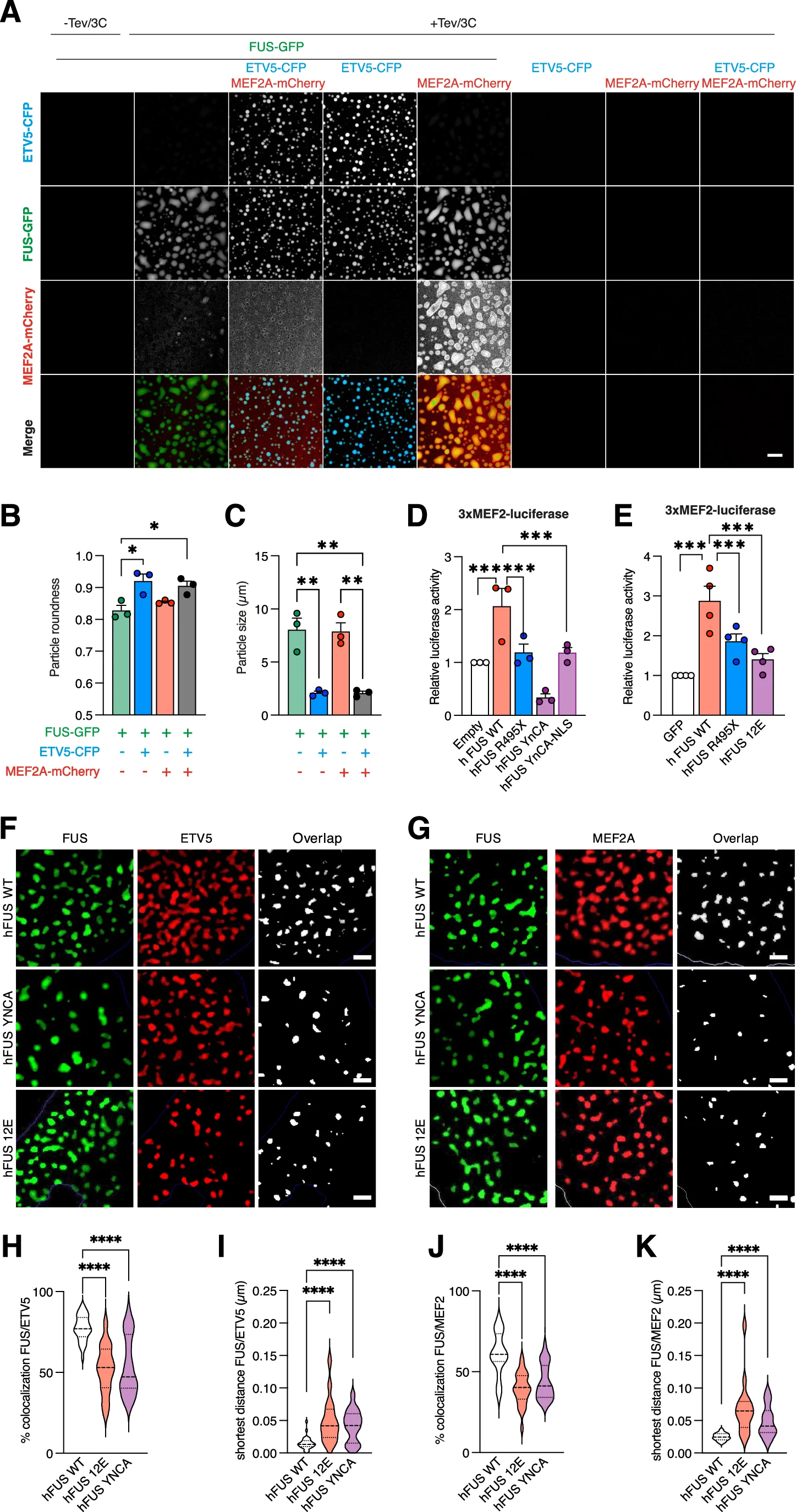
FUS phase separation properties are required for MEF2A/ETV5 transactivation. (**A**) Representative confocal images of FUS-EGFP condensates (5 µM) in absence or presence of ETV5-CFP (3 µM) and/or MEF2A-mCherry (0.85 µM) following protease-mediated cleavage of the MBP-solubility tags. No condensates were observed for ETV5 or MEF2A alone or mixed together in absence of FUS condensates. Bar, 10 µm. (**B**,** C**) Quantification of FUS-EGFP condensate roundness (**B**) and size (**C**). Mean ± SEM of three independent replicates with at least 179 condensates in total from ≥3 different fields of view analyzed. One-way ANOVA with Tukey’s multiple comparison test. (**B**) FUS vs FUS + ETV5: *p = 0.0121, FUS vs FUS + ETV5 + MEF2A: *p = 0.0318. (**C**) FUS vs FUS + ETV5: **p = 0.0013, FUS vs FUS + ETV5 + MEF2A: **p = 0.0013, FUS + MEF2A vs FUS + ETV5 + MEF2A: **p = 0.0016. (**D**,** E**) Light units relative to Empty vector in C2C12 cells 24 h after transfection of *3xMEF2* luciferase plasmid and an expression plasmid for either hFUS WT, hFUS R495X, LLPS deficient hFUS (hFUS YnCA), LLPS-deficient hFUS with SV40 NLS or empty control plasmid (**D**) or hFUS WT, hFUS R495X, LLPS deficient phosphomimetic variant 12E hFUS (hFUS 12E), or GFP expression plasmid (**E**). Nested one-way ANOVA with Tukey’s multiple comparisons test. (**D**) Empty vs hFUS WT: ***p < 0.0001; hFUS WT vs hFUS R495X: ***p = 0.0002; hFUS WT vs hFUS YNCA NLS: ***p = 0.0002. (**E**) GFP vs hFUS WT: ***p < 0.0001; hFUS WT vs hFUS R495X: ***p < 0.0001; hFUS WT vs hFUS R495X: ***p < 0.0001. Each dot represents the mean of an individual experiment (*n* = 3, (**D**); *n* = 4 (**E**)), each consisting of 3–4 technical replicates. Data represent the mean ± SEM. Expression of human wild-type FUS but neither R495X nor two different LLPS-deficient FUS variants increases activity of the MEF2 reporter. (**F**,** G**) HEK293 cells were transfected with Myc-tagged versions of either hFUS WT or LLPS-deficient hFUS YnCA and hFUS 12E (green) and HA-tagged versions of either ETV5 (red, **F**) or MEF2A (red, **G**). HA (red) and Myc (green) immunofluorescence were imaged using STED. Representative STED image of a magnified regions of a nucleus of a transfected cell is shown. Colocalized regions between FUS and ETV5 or MEF2 are highlighted in white. Scale bar, 0.4 µm. (**H**–**K**) Percentage of FUS signal colocalizing with ETV5 (**H**) or MEF2A (**J**) defined by the proportion of FUS found in close spatial association with either ETV5 (**H**) or MEF2A (**J**). Panels (**I**) and (**K**) show the mean shortest distances from individual FUS puncta to the nearest ETV5 (**I**) or MEF2A (**K**) puncta and vice versa, providing a quantitative measure of their nanoscale proximity. All quantifications were performed on STED microscopy images processed and analyzed using Imaris. 32–43 cells analyzed per condition in two independent experiments. All comparisons in panels (**H**–**K**), ****p < 0.0001 by Kruskal–Wallis followed by Dunn’s multiple comparison test. Violin plots show median and quartiles. Source data are available online for this figure.

**Figure EV4:**
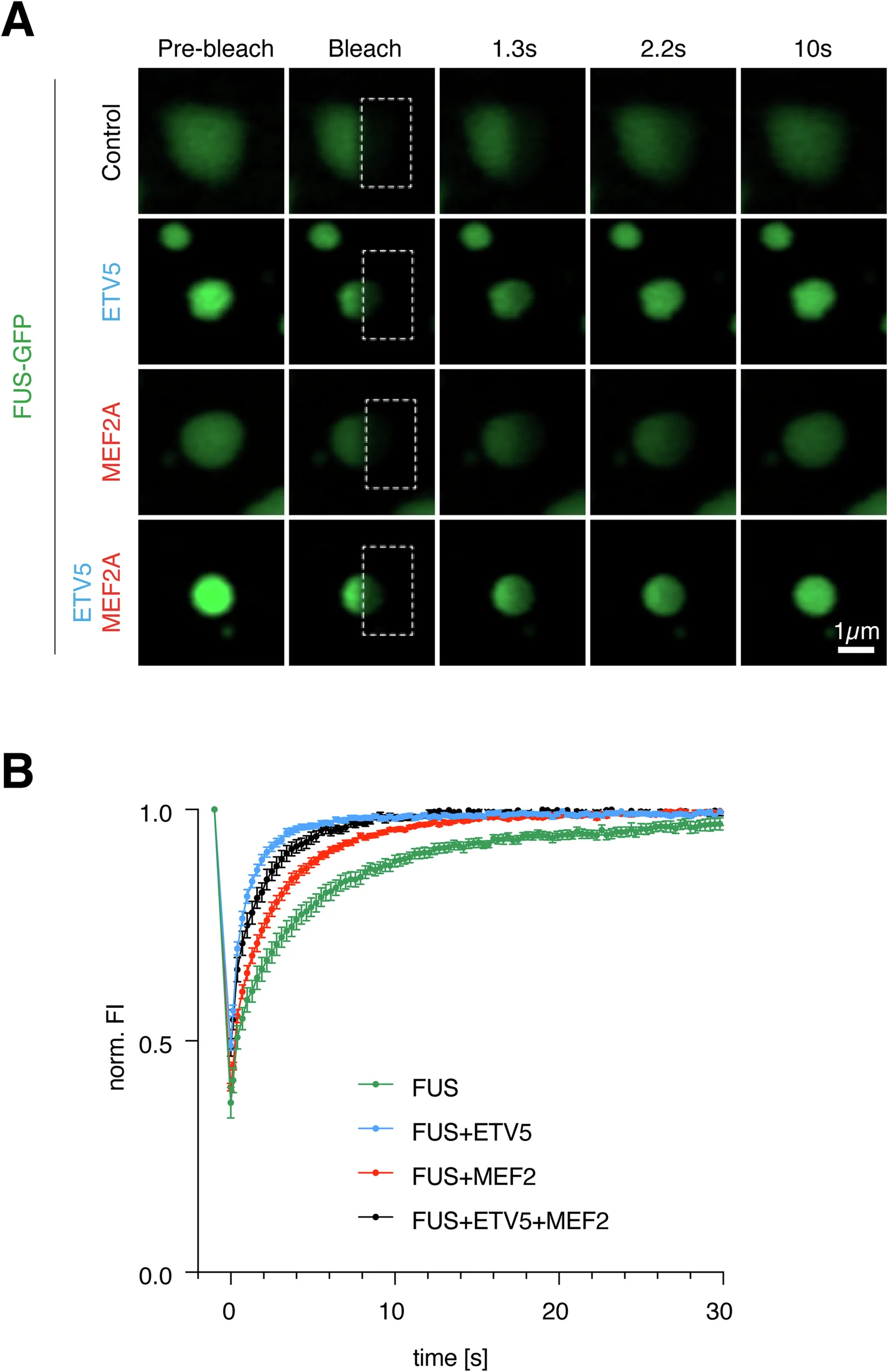
effects of MEF2A and ETV5 on FUS liquidity. (**A**) Representative FRAP images of GFP-FUS condensates (15 µM) at the indicated time points, in the absence or presence of ETV5 and/or MEF2A. Dotted box indicates the bleached area (half of the condensate). Scale bar: 1 µm. (**B**) FRAP recovery curves following half-bleaching of GFP-FUS condensates in absence or presence of ETV5 and/or MEF2A. Shown is the mean ± SEM of 19–25 condensates out of a total of three independent experiments. Source data are available online for this figure.

FUS is known to undergo liquid–liquid phase separation (LLPS) at physiological concentrations (Hofweber et al, 2018) and LLPS is critically involved in chromatin compartmentalization during transcription (Rippe, 2022) and other gene regulatory processes (Hirose et al, 2023). We thus hypothesized that FUS LLPS could be critical in mediating ETV5 and/or MEF2 transcriptional function. Interestingly, both ETV5 and MEF2A are enriched in intrinsically disordered regions, as predicted using PONDR (Garner et al, 1999) (Appendix Fig. S8A) and were predicted to undergo phase separation using PSP predictor (Chu et al, 2022) (Appendix Fig. S8B). We produced fluorescently tagged versions of ETV5 (with CFP) and MEF2A (with mCherry). Despite LLPS predictions, neither ETV5-CFP nor MEF2A-mCherry or the combination of both proteins were able to form condensates on their own, at least at low µM concentrations after 60 min (Fig. 8A). To assess whether presence of FUS might trigger LLPS of ETV5 and/or MEF2A, we introduced MBP-FUS-GFP (Hofweber et al, 2018), whose LLPS can be induced by Tev protease-mediated cleavage of the MBP-solubility tag. When FUS LLPS was triggered in presence of ETV5-CFP or MEF2A-mCherry, each of these proteins partitioned into FUS condensates, suggesting that ETV5 and MEF2A might get recruited and concentrated at promotors through FUS condensation. While MEF2A did not affect FUS droplet formation and morphology, the presence of ETV5 led to smaller and rounder FUS droplets (Fig. 8A–C). To address properties of FUS condensates in absence or presence of ETV5 and/or MEF2A, we subsequently performed fluorescence recovery after photobleaching (FRAP) following “half-bleach” of FUS-EGFP condensates. In presence of ETV5 and/or MEF2A, FUS shows increased internal mobility within the condensates indicating the presence of those factors does not result in enhanced solidification of FUS (Fig. EV4A,B).

To demonstrate a causal role of LLPS properties of FUS in MEF2-driven transcription, we took advantage of the previous characterization of two sets of LLPS-deficient mutants of FUS. Abrogation of LLPS properties of FUS, through the mutation of 7 tyrosine residues to alanine (Qamar et al, 2018) abrogated FUS-mediated activation of 3xMEF2-luc reporter activity, even after addition of a SV40 NLS to force entry of the LLPS-deficient FUS in the nucleus (Fig. 8D). Similarly, a FUS variant expressing phosphomimetic mutations (12E FUS) leading to disruption of LLPS (Monahan et al, 2017) abrogated FUS-mediated activation of the 3xMEF2-luc reporter activity (Fig. 8E). Importantly, this lack of activation of MEF2-driven transcription was not due to lack of protein expression, as LLPS-deficient mutants increased FUS protein levels in transfected cells (Appendix Fig. S3A,B), or to lack of nuclear import as these mutants properly localized to the cell nucleus (Appendix Fig. S3C). To further confirm the spatial proximity between FUS, ETV5, and MEF2A and its dependency to LLPS properties of FUS, we used STED microscopy, which enables nanoscale resolution of subcellular structures. HEK293 cells were transfected with tagged expression vectors of FUS or its LLPS-deficient mutants, along with either HA-ETV5 (Fig. 8F) or FLAG-MEF2A (Fig. 8G). As expected, FUS condensates were nuclear and smaller in size and decreased in density when FUS carried LLPS-deficient mutations (Appendix Figs. S9 and S10A). To assess potential colocalization between FUS and either ETV5 or MEF2A, we calculated the “Overlapped Area Ratio to Surfaces” parameter, which quantifies the ratio of overlapping surface area between the two protein volumes relative to the total surface area of each protein, thus providing a normalized measure of their spatial proximity. Quantitative analyses revealed that WT FUS exhibited significantly greater overlap with ETV5 compared to the LLPS-deficient mutants E12 and YNCA (Fig. 8H). To complement these colocalization measures, we quantified the shortest distance between surfaces, defined as the minimal distance separating the FUS and ETV5 signals. This metric offers a sensitive estimate of nanoscale proximity, even in the absence of direct overlap. As expected, mutants showed significantly increased average distances between FUS and ETV5 (Fig. 8I). Importantly, similar results were observed for MEF2A (Fig. 8J,K), with reduced colocalization and increased separation distances for the LLPS-deficient FUS mutants compared to wild-type, and ETV5 and MEF2A condensates were smaller when expressed with LLPS-deficient FUS as compared to expression with wild-type FUS (Appendix Fig. S10A,B). Together, these findings indicate that the ability of FUS to undergo LLPS is crucial for maintaining close spatial associations with both ETV5 and MEF2A within the nuclear environment. Thus, we propose that LLPS properties of FUS may help to recruit MEF2A and ETV5 and that FUS LLPS is required for FUS-mediated activation of these transcription factors.

## Discussion

Our study identifies a critical function of FUS in muscle development and differentiation through co-activation of key transcription factors involved in muscle differentiation. Specifically, FUS-mediated phase separation of ETV5 and MEF2 promotes the transcription of their target genes. These findings have major implications for our understanding of physiological functions of FUS and could have consequences for *FUS-*ALS.

Our results demonstrate that FUS is critical in muscle development in multiple species. In mice, ablation of *Fus* or deletion of its nuclear localization sequence results in structural muscle defects including defects in sarcomeric and mitochondrial ultrastructure. In Drosophila, muscle selective deletion of the *Fus* ortholog leads to similar alterations in early (Catinozzi et al, 2020) and late muscle development (this study). This novel role for FUS in muscle development and maintenance adds on to the increasing evidence that mutations in ALS-associated genes may also lead to prominent muscle phenotypes. Indeed, mutations in *MATRN3* (Malik and Barmada, 2021), *HNRNPA1* (Kim et al, 2013), or *CHCHD10* (Bannwarth et al, 2014) lead to phenotypes ranging from pure myopathic syndromes to complex mitochondrial phenotypes and multi-system proteinopathy (Korb et al, 2021; Shefner et al, 2023). Mutations in *TARDBP*, encoding TDP-43, have also been described in patients with myopathy (Ervilha Pereira et al, 2023; Zibold et al, 2023) which may result from disruption of TDP-43’s function in RNA granules involved in muscle development and regeneration (Vogler et al, 2018). To our knowledge, mutations in *FUS* in patients with a primary myopathic phenotype have not yet been identified, although reports exists of clinical myopathic features in *FUS*-ALS patients (Yamamoto-Watanabe et al, 2010) and of altered neurophysiological or histological muscle properties in *FUS*-ALS patients, suggesting either initial or accompanying myopathy in these patients (Picchiarelli et al, 2019; Rademakers et al, 2010; Yu et al, 2022).

**Figure EV5:**
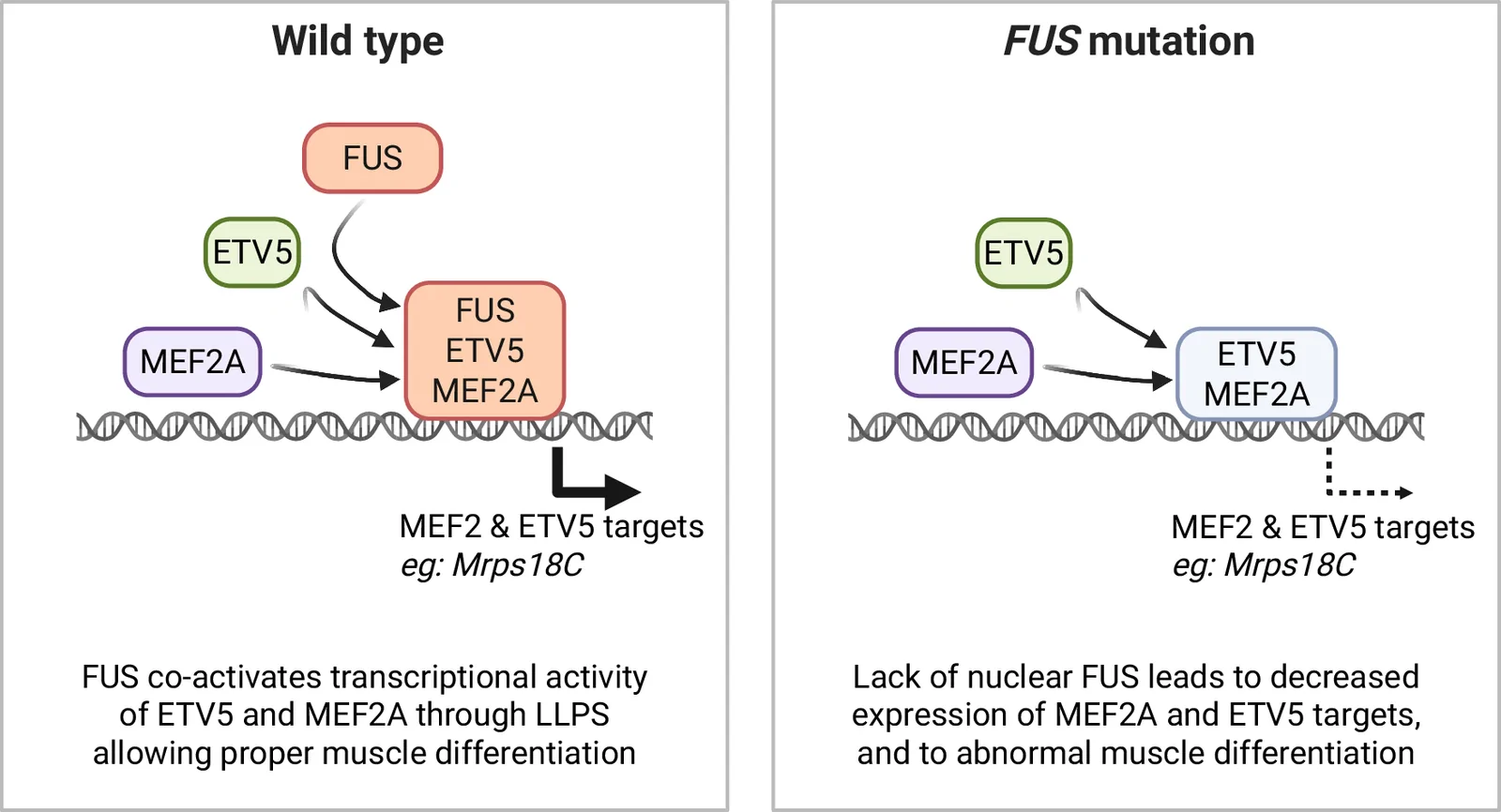
FUS phase separation properties are required for ETV5 and MEF2A to transactivate key muscle differentiation pathways. FUS mutations compromise muscle differentiation.

Our study identifies two different transcription factors interacting with FUS, which could contribute to the muscle phenotype (Fig. EV5). First, expression of genes controlled by MEF2 transcription factors, in particular MEF2A, is severely decreased in three models of FUS depletion or mutation. Interestingly, muscle defects observed upon *Fus* loss or mutation, are not recapitulated by single ablation of either *Mef2a* or *Mef2d* (Potthoff et al, 2007), yet *Mef2a* ablation leads to a similar developmental defect in cardiac muscle (Naya et al, 2002). It is possible that FUS is required for the transcriptional activity of both MEF2A and MEF2D, and that loss of FUS leads to functional loss of both MEF2 isoforms and a more drastic phenotype than loss of the single MEF2 isoforms. Alternatively, other transcription factors, such as *Mef2c* or *Myog* may also require FUS, leading to a more severe muscle phenotype in *Fus* mutant mice than in mice with a single mutation of any of these transcription factors.

Notably, we did not observe a significant enrichment of FUS on MEF2 genomic binding sites, while FUS binding was very specifically associated with PEA3 transcription factor binding sites. A possible explanation is that FUS may only indirectly associate to its chromatin binding sites through binding of transcription factors. It is possible that the physical interaction between ETV5 and FUS is stronger than the looser FUS/MEF2 interactions, leading to better preservation through chromatin immunoprecipitation steps. Our observation of FUS binding to a subset of PEA3 transcription factor binding sites in muscle cells is consistent with our recent results in hippocampus (Tzeplaeff et al, 2023), and is also in line with our previous observation that FUS transactivates subsynaptic genes in an ERM/ETV5-dependent manner (Picchiarelli et al, 2019). Despite the binding of FUS to PEA3 transcription factor target genes, the transcriptional outcome of FUS loss or mutation on these specific genes remains modest. We hypothesize that loss of FUS may be compensated by other FET proteins, such as EWSR1 or TAF15. It is also possible that a strong transcriptional response induced by FUS requires MEF2 binding. Consistent with this hypothesis, downregulation of genes bound by FUS was much stronger when MEF2A was also binding to these genes, suggesting that MEF2A dependency was critical in driving the transcriptional defect upon loss of FUS. Importantly, our results show that MEF2 and PEA3 transcription factor pathways intersect.

We found that FUS, MEF2A, and ETV5 physically interact in vitro, and cooperatively bind to FUS DNA-binding sites. Furthermore, both ETV5 and MEF2A partition into FUS condensates. While ETV5 potently modified FUS condensate morphology and appeared to make them smaller, MEF2A did not modify the condensate shape. It seems possible that FUS and ETV5 thereby potentiate MEF2A transcriptional response through modification of condensate dynamics. Consistent with this notion, super-resolution imaging demonstrated a close proximity between FUS, MEF2A, and ETV5 in transfected cells, that was disrupted when LLPS-deficient FUS was expressed (Fig. 8). It is important to note that our results were mostly obtained upon overexpression of FUS, MEF2A, and ETV5, and that it will be critical to demonstrate these interactions at native endogenous expression levels. Further research is needed to elucidate the relationships between these 3 proteins and their LLPS behavior in transcriptional regulation of muscle development.

FUS, MEF2 and PEA3 transcription factors are broadly expressed, and our findings could thus have consequences beyond muscle development. In adult muscle, both *Fus* and *Mef2a* are expressed in myonuclei, with an enrichment in subsynaptic nuclei (Huang et al, 2020; Petrany et al, 2020; Picchiarelli et al, 2019), while *Etv5* expression is restricted to subsynaptic nuclei (Hippenmeyer et al, 2007). This restricted pattern of expression in adult muscle might explain why haploinsufficiency of *Etv5* in *Fus*^∆NLS/+^ mice leads to defect in muscle endurance rather than muscle strength. Indeed, it is possible that the FUS/MEF2A/ETV5 pathway is more relevant for regulating gene expression in subsynaptic than extrasynaptic nuclei in adults, consistent with our previous study (Picchiarelli et al, 2019). Beyond skeletal muscle, both MEF2 and *PEA3* transcription factors are expressed in the central nervous system and have been involved in synaptic plasticity (Assali et al, 2019) and neuronal specification (Dalla Torre di Sanguinetto et al, 2008). It will be important to determine whether FUS also co-activates MEF2 and PEA3 transcription factors in neurons.

We also observed that mutation of one allele of *FUS*, either in *FUS*-ALS patients or in *Fus*^*∆NLS/+*^ mice, significantly compromised adult muscle ultrastructure. However, our observation of similarly damaged muscle ultrastructure in sporadic ALS patients suggests that these alterations could be either primary or secondary to denervation (Shefner et al, 2023). Future studies should determine the relevance of these developmentally relevant pathways to late-onset muscle weakness and motor neuron degeneration.

Here we show that loss of FUS nuclear function triggers cell autonomous detrimental effects in muscles. This evidence has an immediate translational importance as current strategies to treat *FUS*-ALS are based on antisense oligonucleotides (ASOs) to reduce FUS expression (Lopez-Erauskin et al, 2018; Shneider et al, 2025). The rationale for lowering strategies in *FUS-*ALS is supported by several studies, including ours, showing that *FUS* mutation leads to dominant gain of function responsible for motor neuron loss (Lopez-Erauskin et al, 2018; Scekic-Zahirovic et al, 2017; Scekic-Zahirovic et al, 2021; Scekic-Zahirovic et al, 2016; Zhang et al, 2020). Intrathecal administration of non-allele-specific ASOs targeting FUS is currently tested in *FUS-*ALS patients (Korobeynikov et al, 2022). Considering our observation that loss of FUS in skeletal muscle may have deleterious consequences, it will be important to evaluate possible muscle damage as possible adverse effects of chronic *FUS* downregulation.

In summary, we show here that FUS is critical for muscle development, and provide evidence that this occurs, at least in part, through co-activation of two transcription factors, MEF2A and ETV5 (Fig. EV5).

## Methods

**Table:** Reagents and tools table

Reagent/Resource	Reference or Source	Identifier or Catalog Number
**Experimental models**		
*Fus*^*∆NLS/+*^ mice	(Scekic-Zahirovic et al, 2016)	Custom
*Fus*^*−/−*^ mice	(Scekic-Zahirovic et al, 2016)	Custom
*MyoD*^*iCre*^ mice	(Kanisicak et al, 2009)	#014140
*Erm*^*−/+*^ mice	(Chen et al, 2005)	#022300
UAS-FLP flies	BDSC	stock 4539
UAS-Ets96B-RNAi-1	BDSC	stock 34783
UAS-Ets96B-RNAi-2	BDSC	stock 31935
C2C12 cells	ATCC	CRL-1772
HEK293 cells	ATCC	CRL-1573
**Recombinant DNA**		
pCMV-Myc-FUS	Thermo Scientific	Custom
pCMV-Myc-FUS-R495X	Thermo Scientific	Custom
pCMV-MycFUS YnCA	Thermo Scientific	Custom according to Qamar et al (2018)
pCMV-MycFUS YnCA-SV40 NLS	Thermo Scientific	Custom according to Qamar et al (2018)
pCMV-MycFUS 12E	Thermo Scientific	Custom according to Monahan et al (2017)
pCGN-MEF2A α2β	Todd Gulick	(Zhu et al, 2005)
pGL3-MEF2-luc	Addgene	32967
Mrps18C-luciferase	Gene Copoeia	
pTarget ETV1	Home made subcloning	Custom
pTarget ETV4	Home made subcloning	Custom
pTarget ETV5	Home made subcloning	Custom
**Antibodies**		
@FUS (human tissues)	Sigma	HPA008784
@FUS Chip-seq 1	Bethyl	A300-294A
@FUS Chip-seq 2	Bethyl	A300 293A
@FUS IP	Santa Cruz	sc-47711
@RNA pol II Chip-seq	Millipore	05-623
@ETV5	Proteintech	13011-1-AP
@MEF2A	Santa Cruz	sc-17785
@ChAT	Chemicon	AB144P
@dystrophin	DSHB	Mandra1(7A10)
@Cytochrome c	BD Biosciences	556432
@Actinin	Sigma	A7811
@Myc tag	Cell Signaling	2276
@HA tag	Cell Signaling	3724
@Flag tag	Cell Signaling	14793
Alexa Fluor 488 goat anti-rabbit	Invitrogen	A-11034
Alexa Fluor 647 goat anti-mouse	Invitrogen	A-21235
Donkey anti-goat 568	Thermo Fisher Scientific	A-11057
Goat anti-mouse 568	Thermo Fisher Scientific	A-11031
STAR-ORANGE-goat-anti-mouse	Abberior	STORANGE-1001-500UG
STAR-635P-goat-anti-rabbit	Abberior	ST635P-1002-500UG
**Oligonucleotides and other sequence-based reagents**		
siRNA *Fus*	Horizon	L-051741-00-0020
siRNA *Mef2a*	Horizon	L-041004-00-0020
siRNA *Etv1*	Horizon	L-042535-01-0020
siRNA *Etv4*	Horizon	L-048237-01-0020;
siRNA *Etv5*	Horizon	L-062952-01-0020
negative control siRNA	Horizon	D-001810-01-50
MRPL30 probe	Eurogentec	Custom made
MRPL30 mutant probe	Eurogentec	Custom made
**Chemicals, enzymes and other reagents**		
M1 embedding matrix	Epredia	1310
ProLong Gold Antifade reagent with DAPI	Invitrogen	P36935
Prolong Glass Antifade mounting medium	Thermo Fisher Scientific	P-36980
Stemline medium	Sigma	SA3194
Heparan Sulfate	Sigma	H7640
Accutase	Sigma	A6964
Matrigel-coated plates	Corning	354277
mTeSR medium	Stem Cell Technologies	83850
MitoTracker™ orange CMTMRos	Thermofisher	M7510
DPBS	Gibco	H15-001
FBS	Gibco	10500
BSA	Biomol	01400.100
Normal goat serum	Abcam	Ab7481
Donkey serum	Euroclone	ECS0217D
Triton X-100	VWR	0694-1L
Cresyl Violet acetate	Alfa Aesar	J64318
Glutaric dialdehyde	Thermo Fisher Scientific	233281000
Mono basic sodium phosphate NaH2PO4.1H2O	Merck	6346
Di basic sodium phosphate Na2HPO4.7H2O	Merck	6586
Luciferase assay kit	Promega	E4550
BCA assay	Interchim	UP95424A
protein A/G sepharose beads	Thermo Scientific	3133
tRNA	Sigma	R5636
FUS recombinant protein (IP)	Novus Biologicals	NBP1-42462
FUS recombinant protein (EMSA)	Origene	TP301808
ETV1	Origene	TP310533
ETV4	Origene	TP760035
ETV5	Origene	TP761784
MEF2A	Origene	TP312830
MEF2A-mCherry	IGBMC baculovirus platform	Custom made
ETV5-CFP	IGBMC baculovirus platform	Custom made
MBP-*tev*-FUS-EGFP-*tev*-His_6_		(Hofweber et al, 2018)
His_6_-Tev		(Hofweber et al, 2018)
**Software**		
Fiji/Image J	https://fiji.sc/	
Signal	Written by Thom Oostendorp	
Graphpad Prism	https://www.graphpad.com/	
**Other**		

### Patient biopsies

#### Human skeletal muscle biopsies

For human skeletal biopsy samples, muscle specimens were extracted in an open biopsy of the triceps (FUS1), deltoid (FUS2), vastus lateralis (FUS3), and biceps (FUS4, CNTRLs 1-2) and prepared for cryosection or electron microscopy (Appendix Table S1). Control subjects who presented at our center with chronic muscle pain, showed no myopathic changes in the biopsy results. Skeletal muscle was obtained for diagnostic reasons as a part of the regular clinical work-up, including electromyography and muscle biopsy. This study was conducted according to the Helsinki Declaration. All included patients gave informed written consent for scientific use of remaining biopsy material, which was approved by the local University of Ulm Ethics Committee of the University of Ulm (reference no. 20/10).

#### Immunohistochemistry and histology on patient muscle biopsies

Samples were frozen with chilled isopentane and stored at −80 °C. Serial cuts of 10 μm thick were made using a cryostat (Leica CM 3050S) and placed on Superfrost slides (VWR, 631-0448). Slides were dried for 20 min then fixed with 4% PFA in 0.1 mM sucrose for 15 min at room temperature. Sections were used for immunohistochemistry. After washing with PBS, sections were then permeabilized with 0.1% Triton X-100, and nonspecific binding sites were blocked with 10% goat serum and 5% FBS in PBS for 2 h at room temperature. Rabbit anti-FUS (Sigma, HPA008784, 1:1000) and mouse anti-Cytochrome c (BD Biosciences, 556432, 1:1000) were incubated overnight at 4 °C. After rinsing in PBS, Alexa Fluor 488 goat anti-rabbit (A-11034) and Alexa Fluor 647 goat anti-mouse (A-21235) (all 1:1000, Invitrogen) were incubated for 2 h at room temperature. Sections were then washed with PBS and water and mounted with ProLong Gold Antifade reagent with DAPI (Invitrogen, P36935).

#### Imaging of human tissues

All fluorescence images were acquired using a Leica TCS SPE II high-resolution spectral confocal microscope with a resolution of 1024 × 1024 pixels. The mean fluorescence intensity of cytochrome c was quantified in human muscle sections. Briefly, five random images were taken for each control and mutant section, followed by selecting regions of interest (ROIs) in each image. To select ROIs, transverse sections of the muscle were specifically chosen, and then ROIs were always placed inside the muscle fiber. Afterwards, mean fluorescence intensity for five ROIs was measured in each picture after applying the same settings to all samples using Image J Fiji software. Sections with Cytochrome c oxidase (COX) histochemical labeling were imaged using the MIRAX scan.

TEM images were acquired using a JEOL 1400 Transmission Electron Microscope (TEM), and the images were digitally recorded with a Veleta camera (Olympus). Quantification of TEM images was performed using Image J Fiji software. In human muscle sections, five images were randomly taken for longitudinal parts of the muscle with the same magnification for each control and patient sample. Aligned Z-disk were counted as normal Z-disks, while fragmented, zigzag, and curvy ones were counted as abnormal Z-disks. The percentage of abnormal Z-disks was determined for each image of the control and mutant samples. Regarding muscle mitochondria, the area of nearly 100 muscle mitochondria from random images of healthy control and FUS patient was measured using Image J.

### Human iPSCs culture and differentiation

#### Human iPSCs

The *FUS* and isogenic control hiPSC lines have been previously described and characterized (Higelin et al, 2016). Briefly, the *FUS* hiPSC line was derived from a 26-year-old patient carrying a de novo frameshift mutation in *FUS* (R495QfsX527 (c.1483delC)). The isogenic control cell line (Corrected^FUS^) was generated by inserting a cytosine in position 1483 and correcting the mutation using Clustered Regularly Interspaced Short Palindromic Repeats (CRISPR) technology. HiPSCs were cultured at 37 °C (5% CO_2_, 5% O_2_) on Matrigel®-coated (Corning, 354,277) 6-well plates using mTeSR1 medium (Stem Cell Technologies, 83,850). At 80% confluence, the colonies were detached using Dispase (Stem Cell Technologies, 07923) and passaged in a 1:3 or 1:6 split ratio.

#### Differentiation of myotubes

Differentiation of myogenic cells from human iPSCs was performed as previously published using a sphere-based culture method (Hosoyama et al, 2014). Briefly, hiPSCs were cultured in Stemline medium (Sigma, SA3194) containing 100 ng/ml FGF-2, 100 ng/ml EGF, and 5 ng/ml heparan sulfate (Sigma, H7640-1mg) in suspension using ultra-low attachment flasks. Cells were split on a weekly basis using a 2 min accutase (Sigma, A6964) digestion, and media were exchanged every second day. After 6 weeks in culture, cells were dissociated using accutase digestion, and filtered through a 30 μm pre-separation filter (Miltenyi Biotec, 130-041-407). Then a total of 250,000 cells per well were seeded on PLO and laminin-coated glass coverslips in 24-well plates. After 8 weeks of differentiation, mature myotubes were obtained and either fixed or lysed for analysis.

#### Mitochondrial network labeling and analysis

Mitochondrial labeling was carried out as previously described (Battaglia et al, 2020). In summary, derived myotube mitochondria were labeled with MitoTracker™ orange CMTMRos (50 nM final concentration, Thermofisher # M7510) for 40 min at 37 °C in muscle differentiation medium. Myotubes cultures were then fixed in 4% PFA for 15 min at room temperature (RT) and imaged. Mitochondrial networks were analyzed using the “MiNA plugin” in Image J which allows the semiautomated analysis of the mitochondrial network, providing a topological skeleton of mitochondria (Sladkova et al, 2015; Valente et al, 2017). Briefly, five images were acquired for the mitochondrial network of control and FUS-mutated-derived myotubes. Particle analysis took place after applying a threshold over the network, allowing measurement of the mitochondrial footprint. All experiments were performed in a minimum of *N* = 3 independent replicates (independent differentiations).

#### Immunocytochemistry in sphere-derived myotubes

Immunostaining was performed as previously described (Picchiarelli et al, 2019). Myotube cultures were fixed in 4% (Merck) and 10% sucrose (Roth, 4621.1) in PBS (DPBS, Gibco, H15-001) for 15 min at room temperature. After washing, samples were permeabilized with 0.2% Triton X-100 (Roche, 10789704001) for 10 min. After blocking with 5% FBS (Gibco, 10500) and 10% goat serum (Millipore, S26-100mL) in DPBS for 1 h, primary antibodies rabbit anti-FUS (Sigma, HPA008784, 1:1000) and mouse anti-actinin (Sigma, A7811, 1:500) were incubated for 48 h at 4 °C. After subsequent washing, the following secondary antibodies were incubated for 1 h at room temperature: Alexa Fluor 488 goat anti-rabbit (A-11034), Alexa Fluor 647 goat anti-mouse (A-21235) (all 1:1000, Invitrogen). Then samples were mounted using ProLong Gold Antifade reagent with DAPI (Invitrogen, P36935).

### Mouse husbandry and behavior

#### Mouse housing and breeding

Mice were housed in the Faculty of Medicine from Strasbourg University, the animal facility of the Max Planck Institute for Molecular Biomedicine, and the animal facility of Radboud University, with 12/12 h of light/dark cycle and unrestricted access to food and water.

*Fus*^∆NLS/+^ knock-in mice were generated in the Institut Clinique de la Souris (ICS, Illkirch, Strasbourg) and were maintained and genotyped as previously described (Picchiarelli et al, 2019; Scekic-Zahirovic et al, 2017; Scekic-Zahirovic et al, 2016). This mouse strain expresses a truncated FUS protein that lacks the PY-NLS, which is encoded by exon 15 of the *Fus* gene. This mutation can be reverted to re-express a wild-type FUS protein upon CRE-mediated recombination (Scekic-Zahirovic et al, 2017; Scekic-Zahirovic et al, 2016). Mice heterozygous and homozygous for the targeted allele will hereafter be referred to as *Fus*^ΔNLS/+^ and *Fus*^ΔNLS/∆NLS^, respectively.

*MyoD*^*iCre/+*^ (FVB.Cg-Myod1tm2.1(icre)Glh/J; Strain #:014140 (Kanisicak et al, 2009; Yamamoto et al, 2009)) and *Erm*^*−/+*^ (Stock Etv5tm1Kmm/J, Strain #:022300) (Chen et al, 2005) mice were purchased from the Jackson Laboratory and maintained in the Animal Research Facility (Centraal Dierenlaboratorium) in Nijmegen. Animal experiments performed in Nijmegen were approved by the national Dutch ethics committee ‘Centrale Commissie Dierproeven’ (Centrale Commissie Dierproeven) under reference number AVD10300 2019 8544.

*Fus* knock-out (*Fus*^*−/−*^) mice were generated by the transgenesis facility of the Max Planck Institute for Molecular Biomedicine, using ES cells obtained from the European Conditional Mouse Mutagenesis Consortium (EUCOMM) and were previously described (Scekic-Zahirovic et al, 2017; Scekic-Zahirovic et al, 2016). Unless otherwise noted, the genetic background of mice used in this study is C57Bl/6J. *Fus*^ΔNLS/∆NLS^*; MyoD*^*iCre/+*^ mice have a C57Bl/6N background and *Fus*^ΔNLS/+^*; Erm*^*−/+*^ have a mixed 50% C57Bl/6N, 50% 129S6/SvEvTac background.

#### Mouse motor behavior

The inverted grid test was performed every 4 weeks from 16 weeks of age until 52 weeks of age. Prior to testing, mice were habituated in the testing room for 30 min. Mice were allowed to hang on an inverted grid for up to 60 s, and the hanging time was recorded and documented. The hanging time of each mouse was evaluated twice per session with 15 min intervals between tests.

#### Electromyography

Mice were anesthetized with intraperitoneal injection of a mixture of ketamine and xylazine (100 mg/kg; 5 mg/kg). Animals were kept on a heating pad. Ten minutes after injection of the anesthesia mixture, the sciatic nerve was supramaximally stimulated to trigger a compound muscle action potential (CMAP) which was recorded from the gastrocnemius muscle. CMAPs were elicited by a single square pulse of 0.045 ms to the sciatic nerve at the sciatic notch level with a stimulation box (GRASS Instruments, SD9), using a monopolar needle electrode inserted at the sciatic notch level and measured by a monopolar needle electrode in the gastrocnemius muscle.

The signal was digitized by an amplifier (BIOPAC systems, MP36R) and the custom-written software “Signal”. The electric signal was analyzed with custom-written scripts in MATLAB, which identified the CMAP peak amplitude. Per animal, the average CMAP amplitude of both legs was calculated and plotted as a single data point.

#### Histological analysis

At 96 weeks of age, mice were transcardially perfused with PBS followed by 1% paraformaldehyde (PFA). Hindlimb muscles (gastrocnemius, extensor digitorum longus, and soleus) were dissected and their weight was immediately determined. The spinal cord was dissected and the lumbar area of the spinal cord was microdissected. Next, muscles and spinal cords were post-fixed in 4% PFA for 1 h on ice. After washes with PBS, tissues were stored in 30% sucrose with 0.02% sodium azide at 4 °C.

#### Muscle fiber area size

Gastrocnemius muscle was used for quantification of the area size of muscle fibers. Muscles were embedded in M1 medium and 20 µm transversal cryosections were prepared using a Leica CM1860 cryostat. To label muscle plasma membranes, immunostaining was performed using an anti-dystrophin antibody. Briefly, muscle sections were incubated at room temperature and permeabilized for 30 min with 0.3% Triton X-100 in PBS. After blocking for 1 h at room temperature in blocking buffer (5% normal goat serum, 1% BSA and 0.3% Triton X-100 in PBS), sections were incubated with mouse anti-dystrophin primary antibody (MANDRA1, DSHB, 1:20) primary antibody in blocking buffer at 4 °C overnight. Sections were subsequently washed with 0.3% Triton X-100 in PBS (3 × 10 min) and incubated with secondary antibody (Goat anti-mouse 568, Thermo Fisher Scientific, A-11031, 1:500) in blocking buffer for 2 h at room temperature. Sections were washed in 0.3% Triton X-100 in PBS (3 × 10 min) and mounted in Prolong Glass Antifade mounting medium (Thermo Fisher Scientific, P-36980).

Dystrophin-stained sections were imaged at 10× on a Leica Sp8 liachroic confocal microscope. Per mouse, 2 muscle sections from the middle of the muscle (largest total muscle area size) were imaged using the tilescan function. The muscle fiber area size was determined by delineating a total of 250 muscle fibers per muscle section. Hereby, five defined locations across the gastrocnemius muscle were selected and in each location 50 fibers were measured. For delineation of muscle fibers, a Wacom drawing pad was used and the ROIs were saved with the ROI manager. The average of the 2 sections per mouse was plotted as a single data point.

#### Motor neuron number in lumbar spinal cord

Lumbar spinal cords were embedded in M1 medium and 16 µm transversal cryosections were prepared using a Leica CM1860 cryostat. To anatomically identify the lumbar part of the spinal cord, sections were first stained with 0.1% cresyl violet acetate (Nissl staining). On verified lumbar spinal cord sections, motor neurons were labeled by immunostaining with an anti-choline acetyltransferase (ChAT) antibody. Slides were permeabilized with PBS containing 0.5% Triton X-100 for 2 h at room temperature and then blocked for 4 h at room temperature in blocking buffer (2% donkey serum, 1% BSA and 0.5% Triton X-100 in PBS). Subsequently, slides were incubated with goat anti-ChAT (AB144P, Chemicon, 1:250) primary antibody for 72 h at 4 °C. After washes in 0.5% Triton X-100 in PBS (3 × 15 min), slides were incubated with secondary antibody (Donkey anti-goat 568, Thermo Fisher Scientific, A-11057, 1:500) for 2 h at room temperature. After washes in 0.5% Triton X-100 in PBS (3 × 15 min), slides were mounted in Prolong Glass Antifade mounting medium (Thermo Fisher Scientific, P-36980).

For quantification of the number and area size of α-motor neurons in the ventral horn, sections were imaged on a Leica Sp8 liachroic confocal microscope using a 20× objective. Counting of α-motor neurons was performed in the L1–L3 ventral horn region in every tenth section for eight to ten sections in total per animal. Only ChAT-positive cells located in the ventral horn and with an area size of ≥300 μm^2^ were counted as α-motor neurons. The average of the eight to ten sections per animal was plotted as a single data point. ImageJ/Fiji software was used to measure the area size of ChAT-positive cells using a Wacom drawing pad and the ROI manager in Fiji.

### *Drosophila* experiments

Flies were kept in a temperature-controlled incubator with 12 h on/off light cycle at 25 °C. To induce myocyte-specific *caz* inactivation, *caz*^*FR*T^; MHC-GAL4,UAS-gma-GFP/Tm6b,Tb females were crossed to UAS-FLP (BDSC stock 4539) males and non-Tubby male offsprings was used for the experiments. For Ets96B knockdown experiments, UAS-Ets96B-RNAi-1 (BDSC stock 34783) and UAS-Ets96B-RNAi-2 (BDSC stock 31935) were crossed to MHC-Gal4, UAS-gma-GFP.

#### Imaging of dorsal abdominal muscles

To image dorsal abdominal muscles, male larvae were selected and placed in a new vial containing standard *Drosophila* medium supplemented with yeast paste. White prepupae were collected on wet Whatman filter paper in petri dishes and kept at 25 °C for 96 h. After 96 h, pharate adults were dissected out from the pupal case and placed in an approximately 2 mm furrow of a plastic slide with a drop of 60% glycerol. Images were acquired using a Leica SP8 laser scanning confocal microscope. For quantifications, dorsal abdominal muscles (DAMs) were counted using Fiji (http://fiji.sc/Fiji) by going through Z-stacks.

#### Motor performance assay

To evaluate motor performance, male flies were collected within 48 h after eclosion and housed in groups of 10 individuals. Motor performance of 7-day-old flies was evaluated as described previously (Niehues et al, 2015). Average climbing speed (mm/s) was determined and compared between genotypes.

#### Adult offspring frequency and life span analysis

To determine adult offspring frequencies, the number of adult flies per genotype, eclosing from relevant crosses were counted daily for 9 days.

For life span analysis, male flies were collected on the day of eclosion and housed at a density of 10 flies per vial. The number of dead flies was counted every day and flies were transferred to fresh food vials every 2–3 days.

### C2C12 myoblast culture experiments

#### C2C12 myoblast culture

C2C12 myoblast cells were purchased from ATCC (ATCC® CRL-1772™). Cells were cultured in Dulbecco’s modified Eagle’s medium containing 10% Fetal Bovine Serum (Fisher Scientific, 11531831) and 1% Penicilin-Streptomycin (Sigma, P4333) at 37 °C in an incubator with 5% CO_2_. Between 60 and 80% of confluency, myoblasts were differentiated into myotubes with differentiation medium (identical medium, with 0.1% Fetal Bovine Serum). Culture medium was changed every day and transfections were performed between 5–15 passages.

#### siRNA transfection

C2C12 were cultured in 6-well plates until 60% of confluency. Cells were transfected with siRNA in differentiation medium using Lipofectamine RNA iMAX (Fischer Scientific SAS, 13778150) according to the manufacturer’s instructions. siRNA against *Fus*, *Mef2a*, *Etv1*, *Etv4*, *Etv5* and negative control siRNA were provided by Horizon (L-051741-00-0020; L-041004-00-0020; L-042535-01-0020; L-048237-01-0020; L-062952-01-0020; D-001810-01-50, respectively. Cells were harvested 48 h after transfection.

#### Plasmid transfection and luciferase assay

C2C12 were cultured in 24-well plates until 80% of confluency. Transfections were performed in differentiation medium with expression and reporter plasmids using TransIT-X2 (MIR6000, Myrus) according to the manufacturer’s instructions.

Expression vectors used for transfections were: pCMV empty plasmid, pCMV-Myc-FUS (expressing N-terminal myc-tagged human wild-type FUS), pCMV-Myc-FUS-R495X, pCGN-MEF2A α2β (Zhu et al, 2005). pCMV-MycFUS YnCA, pCMV-MycFUS YnCA-SV40 NLS, and pCMV-MycFUS 12E were synthesized by Thermo Scientific using previously published sequences (Monahan et al, 2017; Qamar et al, 2018). Reporter plasmids used are pGL3-MEF2-luc (termed 3xMEF2-luciferase, Addgene, 32967) and *Mrps18C*-luciferase (Gene Copoeia). After 24 h of transfection, proteins were extracted and luciferase activity was measured (Promega, E4550). For pGL3-MEF2-luc experiments, luciferase activity was normalized by total proteins measured with BCA assay (Interchim, UP95424A). For *Mrps18C*-luciferase, luciferase activity was normalized to alkaline phosphatase activity as an internal control following provider’s instructions.

### Electron microscopy

#### Adult Fus^∆NLS/+^ mice

Mice were anesthetized with intraperitoneal injection of 100 mg/kg ketamine chlorhydrate and 5 mg/kg xylazine and transcardially perfused with glutaraldehyde (2.5% in 0.1 M cacodylate buffer at pH 7.4) Gastrocnemius muscles were dissected and immersed in the same fixative overnight. After 3 rinses in Cacodylate buffer (EMS, 11650), muscles were post fixed in 0.5% osmium and 0.8% potassium ferrocyanide in Cacodylate buffer 1 h at room temperature. Finally, muscles were dehydrated in graded ethanol series and embedded in Embed 812 (EMS, 13940). The ultrathin sections (50 nm) were cut with an ultramicrotome (Leica, EM UC7), counterstained with uranyl acetate (1% (w/v) in 50% ethanol) and observed with a Hitachi 7500 transmission electron microscope (Hitachi High Technologies Corporation, Tokyo, Japan) equipped with an AMT Hamamatsu digital camera (Hamamatsu Photonics, Hamamatsu City, Japan).

#### Embryonic MyoD^iCre/+^; Fus^∆NLS/∆NLS^ mice

Dissected Gastrocnemius samples were fixed by immersion in 2.5% glutaraldehyde in Sorenson buffer (0.1 M, pH 7.4) for 24 h. The samples were washed and stored in Sorenson buffer. Samples were post fixed in 1% osmium tetroxide in 0.1 M cacodylate buffer for 1 h at 4 °C and dehydrated through graded alcohol (50, 70, 90, 100%) and propylene oxide for 30 min each. Samples were embedded in Epon 812. Semi-thin sections were cut at 2 µm with an ultramicrotome (Leica Ultracut UCT) and stained with toluidine blue, and histologically analyzed by light microscopy. Ultrathin sections were cut at 70 nm and contrasted with uranyl acetate and lead citrate and examined at 70kv with a Morgagni 268D electron microscope. Images were captured digitally by a Mega View III camera (Soft Imaging System).

#### Embryonic Fus^+/+^, Fus^∆NLS/∆NLS^, and Fus^*−*/*−*^ mice

Separated leg samples were fixed first in 2% glutaraldehyde, 2% formaldehyde in 0.1 M cacodylate buffer, pH 7.2 for at least 2 h at room temperature, before the muscle part was dissected. Before embedding the tissue was supported by 2% LMP agarose and postfixed in 1% osmium tetroxide including 1.5% potassium cyanoferrate. Samples were subsequently dehydrated stepwise in ethanol with finally 0.5% uranyl acetate en-bloc staining during 70% ethanol. Then the samples were infiltrated in epon. Ultrathin sections of the polymerized sample blocks were cut in different orientations. Representative images were obtained with an electron microscope (Tecnai 12-biotwin, Thermo Fisher Scientific, the Netherlands) with a ditabis plate camera (Ditabis, Pforzheim, Germany).

### Molecular biology and bioinformatics

#### Chromatin immunoprecipitation

Chromatin immunoprecipitation was performed in C2C12 as previously described (Tzeplaeff et al, 2023). C2C12 were cultured in 6-well plates until 70% of confluency, approximately 1.08 × 10^7^cells were used per conditions. After 48 h of differentiation in 0.1%FBS medium, cells were fixed with medium containing 1% formaldehyde for 10 min at room temperature. Cross-linking was stopped by adding cold glycine at 1.25 M during 5 min at 4 °C. Following washing with PBS1x, cells were lysed with Nuclei lysis buffer (50 mM TrisHCL pH 8, 2 mM EDTA pH 8, 0.1% NP40, 10% Glycerol) during 5 min then nuclei were sonicated in sonication Buffer (50 mM TrisHCL pH 8, 10 mM EDTA, 0.3% SDS, 1 M Na-Butyrate) with Bioruptor (Diagenode, B0102001, 3 Cycles of 5 min - 30 s on/30 s off) to reduce DNA length to between 100 and 500 base pairs ideally 250 base pairs.

Before immunoprecipitation (IP), an input DNA fraction was kept and the protein A/G sepharose beads (Thermo Scientific, 3133) were blocked with 10 mg/ml tRNA (Sigma, R5636) and 20 mg/ml bovine albumin serum (Sigma, A3294) during 2 h at 4 °C in order to remove unspecific binding. 30 µg of sonicated cross-linked chromatin was diluted (3×) with low salt Buffer (50 mM TrisHCL pH 8, 150 mM NaCl, 1 mM EDTA, 1% Triton 100X, 0.1% Deoxycholic Acid, 0.1% SDS, protease inhibitors 1X, 5 mM Na-Butyrate), pre cleared during 1 h at 4 °C with blocked sepharose beads then incubated overnight at 4 °C with 5 µg of primary antibodies (Fus 294: Bethyl A300-294A, Fus 293: Bethyl A300, A300 293A, RNA pol II: Millipore 05-623), or without antibody as negative control. Beads were then resuspended in elution buffer (10 mM Tris pH 8, 1 mM EDTA pH 8), and chromatin was reverse cross-linked (0.2 M NaCl, 50 µg/ml of RNase, proteinase K 0.15%) during 3 h at 65 °C. DNA purification was performed with QIAquick ® PCR Purification Kit (28106 – Qiagen).

#### ChIP-Seq analysis

Reads were aligned to the mouse mm10 genome using bowtie2 (Langmead and Salzberg, 2012). BigWig coverage tracks were obtained using deepTools bamCoverage. Peak detection was carried out using macs2 (Zhang et al, 2008). Heatmap images were obtained using deepTools computeMatrix and plotHeatmap (Ramirez et al, 2014). Motif discovery and identification was performed using MEME (Bailey and Elkan, 1994) and AME (Buske et al, 2010). To compare coverage and peak files with human datasets, conversion to the hg38 genome was performed using UCSC Genome Browser liftover (Hinrichs et al, 2006). Venn diagram overlaps were generated with ChIPpeakAnno (Zhu et al, 2010).

#### Public datasets

Murine ETV5 (Zhang et al, 2017), MEF2A (He et al, 2011; Telese et al, 2015), and TAF15 (Alexander et al, 2018) Chip-seq datasets were obtained from CistromeDB (Mei et al, 2017). Human ETV5, MEF2A, and TAF15 Chip-seq binding sites were obtained from ENCODE (Consortium, 2012). ChIP-Seq signal was retrieved from processed bigwig files and plotted on FUS, ETV5, MEF2a peaks using deepTools computeMatrix and plotHeatmaps.

#### RNA-Seq

RNA either from muscle of E18 homozygous *Fus*^ΔNLS/∆NLS^, *Fus*^*−*/*−*^ and control littermates, or from C2C12 cells treated with siRNA against Fus or control siRNA, was extracted using Trizol (Invitrogen). Quality of extracted RNA was determined using the Agilent Bioanalyzer system according to the manufacturer’s recommendations. cDNA libraries after polyA selection were sequenced using an Illumina sequencer with 3–5 biological replicates per condition using single-end 50 bp reads for the mouse muscles samples and 2 × 150 bp for the C2C12 samples.

Reads were aligned to the mouse mm10 genome using STAR (Dobin et al, 2013). Differential expression analysis was carried out using edgeR (Robinson et al, 2010). Heatmaps were generated in R using pheatmap. Integration of C2C12 and E18.5 muscle datasets was performed using batch correction via limma::removeBatchEffect (Ritchie et al, 2015), treating each dataset as a batch and using Fus knock-down, Fus knock-in, and Fus knock out as bridging populations on the one hand, and WT datasets on the other hand. To integrate ChIP-Seq data, GSEA analyses (Subramanian et al, 2005) were performed using the gene names of the top 500 ChIP-Seq peaks as genesets, using vsd transformed and batch-corrected expression data.

### Protein biochemistry and phase separation assays

#### Recombinant proteins

FUS recombinant protein was obtained from Novus Biologicals (NBP1-42462, immunoprecipitation) or Origene (# TP301808, EMSAs). ETV1 (# TP310533), ETV4 (# TP760035), ETV5 (# TP761784) and MEF2A (# TP312830) were purchased from Origen. MEF2A-mCherry and ETV5-CFP were produced with the baculovirus expression vector system and purified by the baculovirus platform of IGBMC (Illkirch-Graffenstaden). Expression of recombinant MBP-*tev*-FUS-EGFP-*tev*-His_6_ and His_6_-Tev was performed as described (Hofweber et al, 2018). In brief, for expression of MBP-*tev*-FUS-EGFP-*tev*-His_6_, the bacterial expression plasmid pMal-*tev*-FUS-EGFP-*tev*-His_6_ was transformed into E. coli BL21-DE3 Rosetta LysS and cells were grown in standard lysogeny broth (LB) medium. At OD600 of ~0.7–0.8, cells were induced with 0.1 mM IPTG for ca 20 h at 12 °C. Cells were lysed in resuspension buffer (50 mM Na_2_HPO_4_/NaH_2_PO_4_, pH 8.0, 300 mM NaCl, 10 µM ZnCl_2_, 40 mM imidazole, +10% glycerol supplemented with 4 mM β-mercaptoethanol and 1 µg/ml each of aprotinin, leupeptin hemisulfate and pepstatin A) using sonication and purified by tandem-affinity purification using Ni-NTA agarose (Qiagen) and amylose resin (NEB). The protein was eluted using 300 mM imidazole and 10 mM maltose, respectively, and eventually dialyzed into storage buffer (20 mM Na_2_HPO_4_/NaH_2_PO_4_, pH 8.0, 150 mM NaCl, 5% glycerol, 1 mM EDTA, 2 mM DTT).

Expression of His_6_-Tev was induced in E. coli BL21 Star using 0.5–1 mM IPTG at 20 °C overnight. Cells were lysed in lysis buffer (50 mM Tris pH 8, 200 mM NaCl, 20 mM imidazole, 10% glycerol supplemented with 4 mM β-mercarptoethanol) by addition of lysozyme and sonication in presence of 0.1 mg/ml RNase A. His_6_-Tev was purified using Ni-NTA agarose, washed using lysis buffer containing 1 M NaCl and eluted in lysis buffer containing 600 mM imidazole. The protein was dialyzed against storage buffer (50 mM Tris pH 8, 150 mM NaCl, 20% glycerol, 2 mM DTT).

His_6_-3C protease was purified by the IMB protein production core facility and stored in 20 mM Tris pH 7.4, 150 mM NaCl, 20% glycerol, 1 mM DTT.

Protein concentrations for MBP-tev-FUS-EGFP-tev-His_6_ and His_6_-Tev were determined from their absorbance at 280 nm using ε predicted by the ProtParam tool. 260/280 nm ratios of purified proteins were ≤0.8.

#### Immunoprecipitation

FUS recombinant protein (1 µg; Novus Biologicals NBP1-42462) was incubated overnight at 4 °C on a rocking platform in presence of ETV5-CFP (1 µg), MEF2A-mCherry (1 µg) and 20 µl of FUS antibody (Santa Cruz sc-47711) or control IgG antibody (Jackson # 115-007-003) in buffer containing 75 mM Hepes pH 7.3, 100 mM KOAc, 1% NP-40, 2.5% glycerol, 0.5 mM DTT and PIC 1X. A/G magnetic bead (20 µl - ThermoFisher Scientific #88802) was added to the sample and placed on a rocking platform at room temperature during 1h30 then the supernatant was removed and the beads were washed three times with RIPA. Finally, beads were resuspended in Laemmli sample buffer and protein were eluted by boiling 10 min at 90 °C and analyzed by SDS-PAGE and immunoblotting using FUS (Santa Cruz sc-47711), ETV5 (Proteintech 13011-1-AP), and MEF2A (Santa Cruz sc-17785) antibodies.

#### EMSA

Oligonucleotides were purchased from Eurogentec. For MRPL30 probe, a single-strand sense (FAM-labeled; 5’-FAM-TTTTAACGGCGTGGTTTGAATGTTGCACTGGGTCCACCTGCCTCTGTCTCCGGCTGCGGGGACGTTCGGA-3’) and antisense (5’-TCCGAACGTCCCCGCAGCCGGAGACAGAGGCAGGTGGACCCAGTGCAACATTCAAACCACGCCGTTAAAA-3’) oligonucleotides were annealed in buffer containing 100 mM Tris, pH 7.5–8, 500 mM NaCl, 10 mM EDTA at 90 °C for 5 min and gradually cooled at room temperature during 45 min. MRPL30 mutant probe was obtained by annealing single-strand sense (FAM-labeled; 5’-FAM- TCCGAACGTCCCCGCAGCCGGAGACAGAGGCAGGTGGACCCAGTGCAACATTCAAACCACGCCGTTAAAA-3’) and antisense (5’-TTTTAACGGCGTGGTTTGAATGTTGCACTGGGTCCACCTGCCTCTGTCTCCGGCTGCGGGGACGTTCGGA-3’) oligonucleotides.

EMSAs were performed in 20 µl reaction volume containing 20 mM HEPES-KOH pH 7.5, 5 mM MgCl_2_, 50 mM KCl, 10 mM DTT, 10% Glycerol. Binding reactions were performed in presence of increasing concentration ETV5 (60, 120, 180, and 240 nM) or constant concentration of ETV1, ETV4, or ETV5 (120 nM) and/or Fus (300 nM) and/or MEF2A (50 nM) and MRPL30 double-strand probe WT or mutant (5 nM/reaction). After 25 min incubation at 25 °C, the reaction mixture was loaded onto a 6% polyacrylamide gel (ThermoFisher Scientific # EC63652BOX) in 0.5x TBE and electrophoresed at 100 V for 2–3 h. The gels were imaged by iBright FL1000 Invitrogen Imaging System and the signal intensity was quantified using the iBright™ Analysis Software.

#### Microscopy condensation assay

Purified MBP-*tev*-FUS-EGFP-*tev*-His_6_, MBP-3C-CFP-ETV5, and MBP-3C-MEF2A-mCherry were mixed at concentrations determined ideal for DNA binding by EMSA in 50 mM Tris pH 7.5 supplemented with 2 mM DTT containing 150 mM NaCl final. Cleavage of the MBP-solubility tag was induced by addition of 1.3 µM His_6_-Tev and/or 1.6 µM His_6_-3C proteases, respectively, in 18-well ibiTreat dishes (Ibidi) and imaged by confocal microscopy using a Leica Stellaris Microscope (Imaging Core Facilty of IMB Mainz) after 45–60 min. Images were acquired using unidirectional scanning at a pixel size of 64 nm using an HC PL APO CS2 63x/1.4 oil objective. Using a white light laser, CFP-ETV5 was excited at 440 nm and detected using a HyDX2 detector (445–742 nm), FUS-EGFP was excited at 489 nm and detected by a HyDS3 detector (494–593 nm), MEF2A-mCherry was excited at 587 nm and detected by a HyDX4 detector (593–750 nm). Images were analyzed using Fiji/Image J built in plug-ins for particle analysis. In brief, first a median filter (3 px) and subsequently a common threshold to include all condensates were applied to all images from the same experiment. Finally, images were converted into binary and watershedding was performed if applicable. Eventually, condensates were analyzed using the “Analyze Particles” plugin.

#### FRAP of FUS-EGFP condensates and analysis

To analyze FUS dynamics in condensates in absence or presence of MEF2A and/or ETV5 by FRAP, condensates of MBP-*tev*-FUS-EGFP-*tev*-His (5 µM) were induced by addition of 2 µM His-Tev protease in absence or presence of 3 µM MBP-*3C*-ETV5-CFP-*3C*-His and/or 0.85 µM MBP-*3C*-MEF2A-mCherry-*3C*-His in condensation buffer (50 mM Tris pH 8, 150 mM NaCl, 2 mM DTT) using Ibidi µ-Slide 18well flat chambers (ibidi, #81826). All reactions contained 1.25 µM 3C-protease to simultaneously induce cleavage of the MBP-solubility tags of ETV5-CFP or MEF2A-mCherry, respectively. FRAP experiments were performed 10–35 min after protease addition on a Zeiss LSM900 equipped with AiryScan2 Detector in SR-4Y-multiplex mode at a pixel size of 43 nm using a Plan-Apochromat 63x/1.40 oil objective, bidirectional scanning at 34.34 ms/frame, a 488 nm laser for excitation/bleaching and a detector window of 498–548 nm. After three pre-bleach images, the bleach of half a condensate was carried out using identical ROI settings for all conditions using the 488 nm laser at higher power and to 50% reduced scan speed for good bleaching efficiency. Post-bleach images were acquired in 0.3 s intervals.

FRAP analysis were performed as described in (Gruijs da Silva et al, 2022) using Image J/Fiji software by calculating the fluorescence intensity over time (I(t)) using the built-in Time Series Analyzer and the following formula:

$${{{\rm{I}}}}\left({{{\rm{t}}}}\right)=\left[{{{{\rm{ROI}}}}}_{1}\left({{{\rm{t}}}}\right)-{{{{\rm{ROI}}}}}_{3}\left({{{\rm{t}}}}\right)\right]/ \left[\left(\right.{{{{\rm{ROI}}}}}_{2}\left({{{\rm{t}}}}\right)-{{{{\rm{ROI}}}}}_{3}\left({{{\rm{t}}}}\right)\right]$$

ROI_1_ corresponds to the averaged gray values of the bleached area, and ROI_2_ to the averaged gray values of the total droplet. ROI_3_ corresponds to the averaged background values. Values were further normalized to the initial fluorescence by dividing I(t) by the mean of the mean gray value of I(t) the three frames acquired before bleaching. Graphs were generated using GraphPad Prism 9.

### Super-resolution imaging of FUS, ETV5, and MEF2A

#### Cell culture and co-transfection

HEK cells were cultured in DMEM supplemented with 10% FBS and 1% penicillin-streptomycin at 37 °C in 5% CO₂. For experiments, cells were seeded onto glass coverslips and, upon reaching ~60–70% confluency, co-transfected with plasmids encoding either FUS-Myc (WT or mutant, 100 ng) together with ETV5-HA or MEF2A-Flag (75 ng), using Lipofectamine 2000 (Invitrogen, Cat. no. 11668019) according to the manufacturer’s protocol. Twenty-four hours post-transfection, cells were fixed and processed for immunofluorescence.

#### Fixation, immunostaining, and mounting

Cells were rinsed with PBS and fixed using 4% paraformaldehyde (PFA) in PBS for 15 min at room temperature. Permeabilization was performed using 0.1% Triton X-100 in PBS for 10 min. Primary antibodies against Myc tag (Cell Signaling #2276) and HA tag (Cell Signaling #3724) or Flag tag (Cell Signaling #14793) were applied for 1 h at room temperature. After three washes with PBS, glass coverslips were incubated for 1 h at room temperature in the dark in the secondary antibodies STAR-ORANGE-goat-anti-mouse (Abberior # STORANGE-1001-500UG) and STAR-635P-goat-anti-rabbit (Abberior #ST635P-1002-500UG). Stained coverslips were mounted in an anti-fade Prolong Gold (Life Technologies # P36930).

#### Super-resolution STED-FLIM imaging

For super-resolution STED imaging, an inverted confocal laser scanning microscope (STELLARIS 8, Leica Microsystems) equipped with a 405 nm laser, a white light laser (440–790 nm), three hybrid detectors (HyD type S and X), and a 100x objective (NA = 1.40, oil immersion, WD = 130 µm) was used. A STED module (Leica Microsystems) with 775-nm pulsed depletion lasers was combined together with a fully fast integrated FLIM module (FAst Lifetime CONtrast “FALCON”, Leica Microsystems).

Images were acquired in a sequential mode. As a first sequence, StarOrange was excited at 590 nm (WLL, 5% laser power) and depleted with 40% of a 775-nm STED laser. In a photon counting mode, HyD-S was used to detect fluorescence emission from 599 to 624 nm. In a second step, Star635P was excited at 635 nm (WLL, 5.5% laser power) and depleted with 26.2% of a 775-nm STED laser. In a photon counting mode, HyD-X was used to detect fluorescence emission from 644 to 750 nm. Acquisitions were performed through a 1024 × 1024 image format, a scan speed at 200 Hz, 8 line accumulations and Airy 1 pinhole. A zoom of 5 was applied to obtain a pixel size of 22.73 nm. Gating between fluorescence lifetime 0.8 ns to 6 ns was activated. Removal of uncorrelated STED process photons through background substraction and weighting of lifetimes was done using TauSTED module by applying 100 Tau Strength filter and 50 denoise parameters. Hoechst was acquired using confocal imaging (Ex 405 nm laser line 5%, HyD-S 421 nm – 464 nm, 3 accumulations).

#### Imaris analysis

STED images were analyzed using Imaris. Nuclei were first segmented by creating masks based on the DAPI staining. Subsequent masks were generated for training the software to recognize the FUS (WT or mutants), ETV5, or MEF2 proteins of interest. After segmentation, colocalization was quantified by calculating the ratio of overlapping signal (“Overlapped Area Ratio to Surfaces”), and spatial relationships were further assessed by measuring the shortest distance between protein-specific signals (“Shortest Distance to Surfaces”). All analyses were performed on at least 30 cells per condition, in 2 independent experiments.

### Statistics

All results are presented as mean ± standard error of the mean (SEM) and differences were considered significant when *p* < 0.05. Significance is presented as follows: *p < 0.05, **p < 0.01, and ***p < 0.001.

Before analysis, a Robust regression and Outlier removal method (ROUT) was performed to detect statistical outliers. This nonlinear regression method fits a curve that is not influenced by outliers. Residuals of this fit were then analyzed by a test adapted from the False Discovery Rate approach, to identify any outliers. All data points that were considered outliers were excluded from further data analysis.

For comparison of two groups, two-tailed unpaired Student’s *t*-test was used in combination with F-test to confirm that the variances between groups were not significantly different.

Comparison for more than two groups was performed using one-way ANOVA and Tukey or Bonferroni’s multiple comparison post hoc test or Kruskal–Wallis as indicated in figure legends. For cell biology and biochemistry experiments, all experiments were performed at least 3 times, each biological replicates consisting in several technical replicates. In these cases, we used a Nested one-way ANOVA with Tukey’s multiple comparisons test, pairing technical replicates of each biological replicate experiment.

For statistical analysis of dorsal abdominal muscle number in *Drosophila* pharate adult flies, two-way ANOVA with Tukey’s multiple comparisons test and Kruskal–Wallis test with Dunn’s multiple comparisons test were used. To analyze muscle misorientation, Wilcoxon signed-rank test was used to evaluate differences as compared to the control genotype, and direct comparison between MHC-GAL4, *caz*^*FRT*^ and MHC-GAL4, *caz*^*FRT*^; UAS-FLP was analyzed by Mann–Whitney test. Chi-square statistics was used to analyze offspring frequency data. For life span analysis, the log-rank test was used to test for statistical significance. Motor performance was analyzed using the Mann–Whitney U test to compare climbing speed of individual flies per genotype and per run. Because all flies were tested in three independent runs, three *P* values were generated per genotype. These *P* values were combined using the Fisher’s combined probability test. For EMG, muscle weight, muscle fiber size and α-motor neuron number, one-way ANOVA was used to determine statistical differences between groups. For analysis of mouse body weight, a mixed-effects analysis was performed with Tukey’s multiple comparisons test. Statistical analysis was performed using Graphpad Prism software.

## Supplementary information


Appendix
Peer Review File
Source data Fig. 1
Source data Fig. 2
Source data Fig. 3
Source data Fig. 4
Source data Fig. 5
Source data Fig. 6
Source data Fig. 7
Source data Fig. 8
EV Figure Source Data
Appendix Figure Source Data
Expanded View Figures


## Data Availability

Datasets generated in this study are deposited in GEO: GSE275325 and GSE308810. Source data for all figures are included in the article or its online Source data files. All reagents, including plasmids and recombinant proteins, are available through the corresponding authors. The source data of this paper are collected in the following database record: biostudies:S-SCDT-10_1038-S44318-026-00874-1.
