# Supplementary material for: FUS controls muscle differentiation through phase separation-mediated recruitment of the transcription factors MEF2 and ETV5
Source: EMBO J. 2026 Jul 22;45(17):6267–98. doi: 10.1038/s44318-026-00874-1 (PMC13534636; doi:10.1038/s44318-026-00874-1)
Supplement: Supplementary file 1 [file 44318_2026_874_MOESM13_ESM.pdf]

# Expanded View Figures

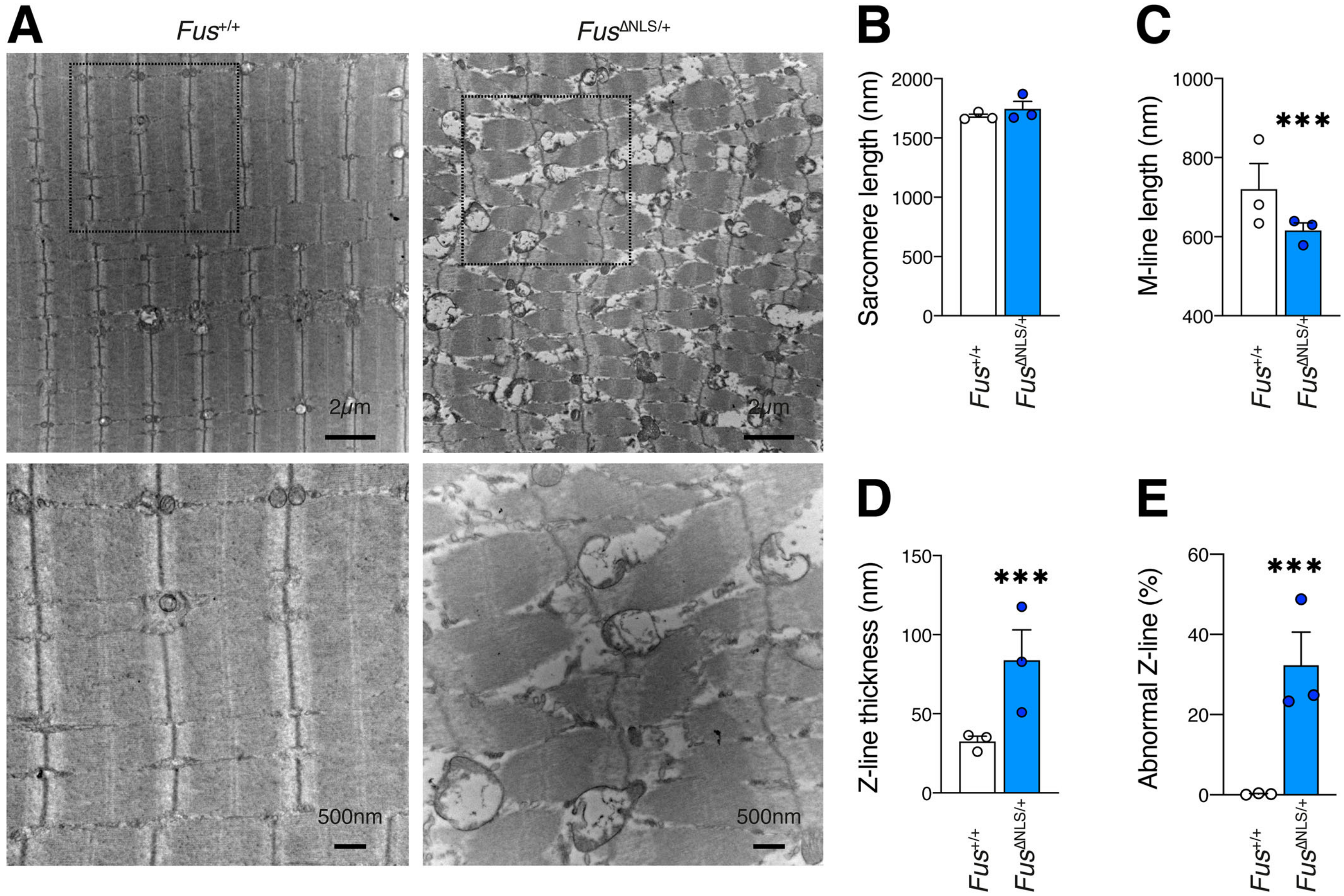


**Figure EV1. Heterozygous *Fus* mutation in mouse leads to muscle ultrastructural defects.**

(**A**) Representative TEM images in gastrocnemius muscle of 22-month-old $Fus^{\Delta NLS/+}$ mice and their control littermates. A higher magnification of the indicated region (dashed box) is shown in the lower panels. (**B–E**) Quantitative analyses of ultrastructural parameters of sarcomeres in adult $Fus^{\Delta NLS/+}$ mice and their control littermates. Dots represent individual animals analyzed ($n = 3$), 20 pictures were analyzed per animal. Data represent the mean ± SEM. All comparisons in (**B–E**) by Nested t-test. (**B**) $p = 0.3917$, (**C**) $^{***}p = 0.004$, (**D**) $^{***}p < 0.0001$, (**E**) $^{***}p < 0.0001$. Source data are available online for this figure.

 © The Author(s)

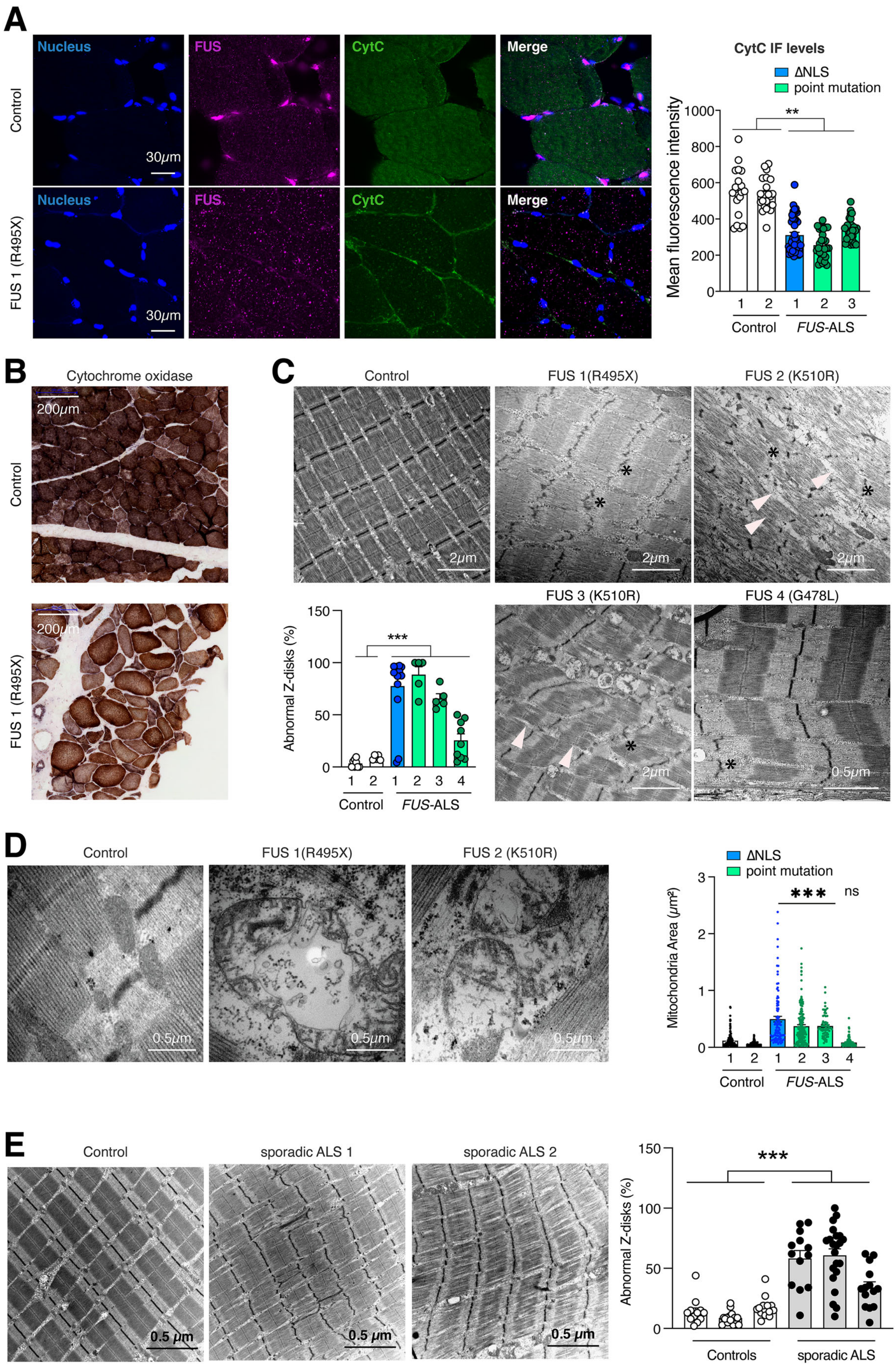
A
Nucleus
FUS
CytC
Merge
Control
FUS 1 (R495X)
30μm
CytC IF levels
ΔNLS
point mutation
Mean fluorescence intensity
**
Control
FUS-ALS
B
Cytochrome oxidase
200μm
C
Control
FUS 1(R495X)
FUS 2 (K510R)
FUS 3 (K510R)
FUS 4 (G478L)
2μm
0.5μm
Abnormal Z-disks (%)
***
D
Control
FUS 1(R495X)
FUS 2 (K510R)
0.5μm
Mitochondria Area (μm²)
ns
E
Control
sporadic ALS 1
sporadic ALS 2
0.5 μm
Abnormal Z-disks (%)
Controls
sporadic ALS

◀ **Figure EV2. FUS mislocalization, abnormal sarcomeric ultrastructure, and mitochondrial defects in *FUS*-ALS and sporadic muscles.**

(**A**) Immunostaining for nucleus (DAPI, blue), FUS (magenta), and cytochrome c (CytC, green) on sections of muscle biopsies from a control individual (upper row) and FUS1 patient carrying a *FUS* truncating mutation R495X (lower row). FUS protein was mainly localized to the nucleus in the control muscle, while cytoplasmic mislocalization was evident in this *FUS*-ALS muscle. Cytochrome c staining was higher in control muscle compared to *FUS*-ALS muscle where it accumulated at the plasma membrane. Scale bar: 30 µm. Right panel shows the quantitative analysis of CytC immunoreactivity in 3 *FUS*-ALS patients. FUS1 patient carries a truncating mutation (ΔNLS, blue), while FUS2 and FUS3 patients carry a K510R missense mutation (green). Data represent the mean ± SEM. Nested t-test, $^{**}p = 0.005$. (**B**) Cytochrome oxidase histochemistry in control and FUS1 patient muscle. Scale bar: 200 µm. Similar results were obtained in FUS2 and FUS3 patients. (**C**) Electron micrographs of muscles in healthy control showing normal ultrastructure with aligned Z-lines, and *FUS*-ALS patients showing extensive myofibrillar disorganization (white arrowheads), including disrupted Z-lines (stars). Lower left panel shows the percentage of fragmented, curvy and/or zigzag Z-lines in controls and *FUS*-ALS patients. FUS4 patient carries a G478L mutation. Truncating mutation (ΔNLS) is indicated in blue, missense mutations are indicated in green. Dots indicate the analysis of different images in the corresponding individuals. Data represent the mean ± SEM. Nested t-test; $^{***}p < 0.0001$. (**D**) Electron micrographs of muscle mitochondria in healthy control showing normal size and structure of mitochondria, and in *FUS*-ALS patients, showing fragmentation, swelling or complete destruction of mitochondria in FUS1, FUS2, and FUS3 patients, while FUS4 patient did not show altered mitochondrial ultrastructure. FUS1 patient carries a R495X truncating mutation (ΔNLS, blue), while patients 2–4 carry point mutations (K510R for patients 2 and 3 and G478L for patient 4). Right panel shows quantification of mitochondrial surface area with a significant increase in mitochondrial size in *FUS*-ALS patients, except for patient FUS4 (ns: not significant). Data represent the mean ± SEM. One-way ANOVA with Tukey's multiple comparisons test: Control versus FUS1, FUS2, FUS3: $^{***}p < 0.0001$. Scale bars: 0.5 µm. (**E**) Abnormal ultrastructure in sporadic ALS muscles. Electron micrographs of muscle in a healthy control showing normal ultrastructure with aligned Z-lines, and a sporadic ALS patient showing myofibrillar disorganization and disrupted Z-lines. Right panel shows the percentage of fragmented, curvy and/or zigzag Z-lines in 3 controls and 3 sporadic ALS patients. Dots indicate the analysis of different images in the corresponding individuals. Data represent the mean ± SEM. Nested t-test; $^{***}p < 0.0001$. Source data are available online for this figure.

 © The Author(s)

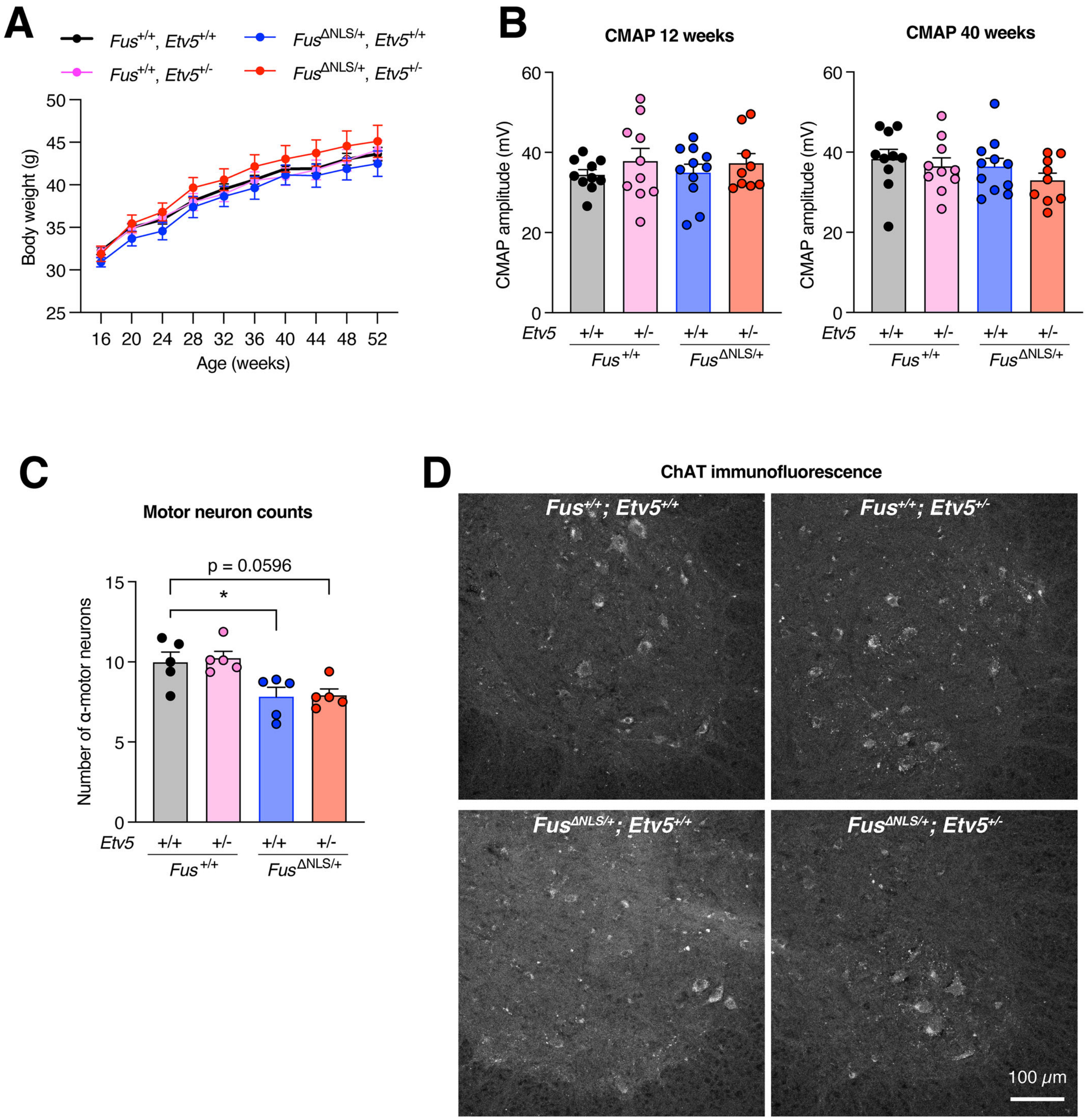


**Figure EV3. Additional characterization of *Etv5* haploinsufficiency in *Fus*$^{ΔNLS/+}$ mice.**

(**A**) Body weight of $Fus^{+/+}$; $Etv5^{+/+}$, $Fus^{+/+}$; $Etv5^{+/-}$, $Fus^{ΔNLS/+}$; $Etv5^{+/+}$ and $Fus^{ΔNLS/+}$; $Etv5^{+/-}$ mice determined every 4 weeks from 16 to 52 weeks of age. $n = 9$–10 mice per genotype. Data represent the mean ± SEM. $p = 0.4799$ for genotype effect by mixed effect analysis with Tukey's multiple comparisons test. (**B**) Compound muscle action potential (CMAP) amplitude in the gastrocnemius muscle as determined by electromyography (EMG) after stimulation of the sciatic nerve of $Fus^{ΔNLS/+}$ x $Etv5^{+/-}$ mice at 12 (left) or 40 (right) weeks of age. $n = 9$–11 mice per genotype. Data represent the mean ± SEM. $p = 0.6677$ (12 weeks) and $p = 0.3952$ (40 weeks) by one-way ANOVA. (**C**) Average number of α-motor neurons (defined as ChAT-positive motor neurons with an area size of ≥300 μm$^2$) per section in the ventral horn of the lumbar spinal cord. $n = 5$ animals per genotype. Data represent the mean ± SEM. One-way ANOVA with Sidak's multiple comparisons test. $Fus^{+/+}$ x $Etv5^{+/+}$ vs $Fus^{ΔNLS/+}$ x $Etv5^{+/+}$ * $p = 0.0418$; $Fus^{+/+}$ x $Etv5^{+/+}$ vs $Fus^{ΔNLS/+}$ x $Etv5^{+/-}$: $p = 0.0596$. (**D**) Representative ChAT immunohistochemistry images in spinal cord of the indicated genotypes. Scale bar: 100 μm. Source data are available online for this figure.

© The Author(s)

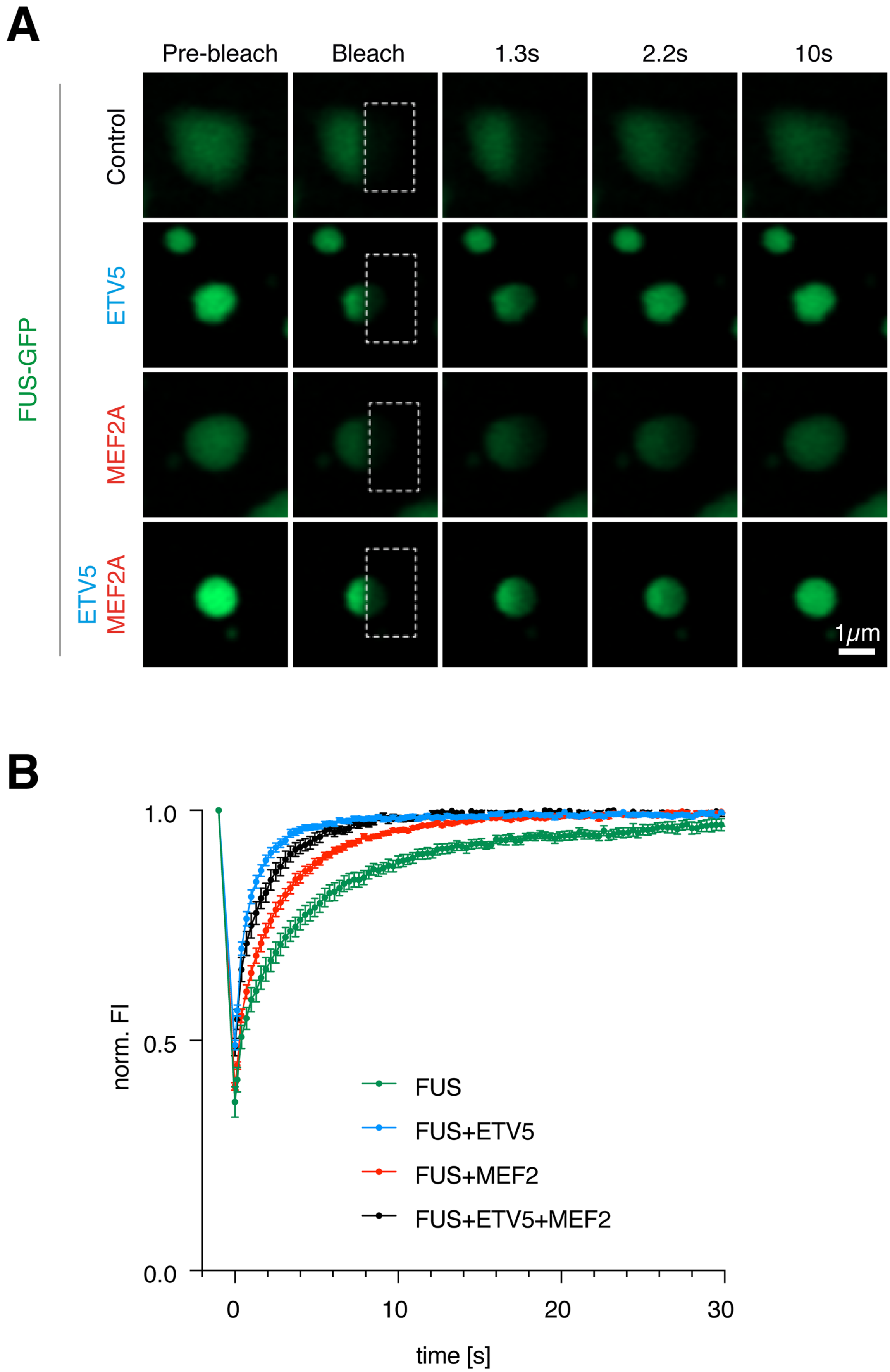
A
Pre-bleach
Bleach
1.3s
2.2s
10s
FUS-GFP
Control
ETV5
MEF2A
ETV5
MEF2A
1μm
B
norm. FI
1.0
0.5
0.0
0
10
20
30
time [s]
FUS
FUS+ETV5
FUS+MEF2
FUS+ETV5+MEF2

 © The Author(s)

**Figure EV4. effects of MEF2A and ETV5 on FUS liquidity.**

(**A**) Representative FRAP images of GFP-FUS condensates (15 µM) at the indicated time points, in the absence or presence of ETV5 and/or MEF2A. Dotted box indicates the bleached area (half of the condensate). Scale bar: 1 µm. (**B**) FRAP recovery curves following half-bleaching of GFP-FUS condensates in absence or presence of ETV5 and/or MEF2A. Shown is the mean ± SEM of 19–25 condensates out of a total of three independent experiments. Source data are available online for this figure.

© The Author(s)

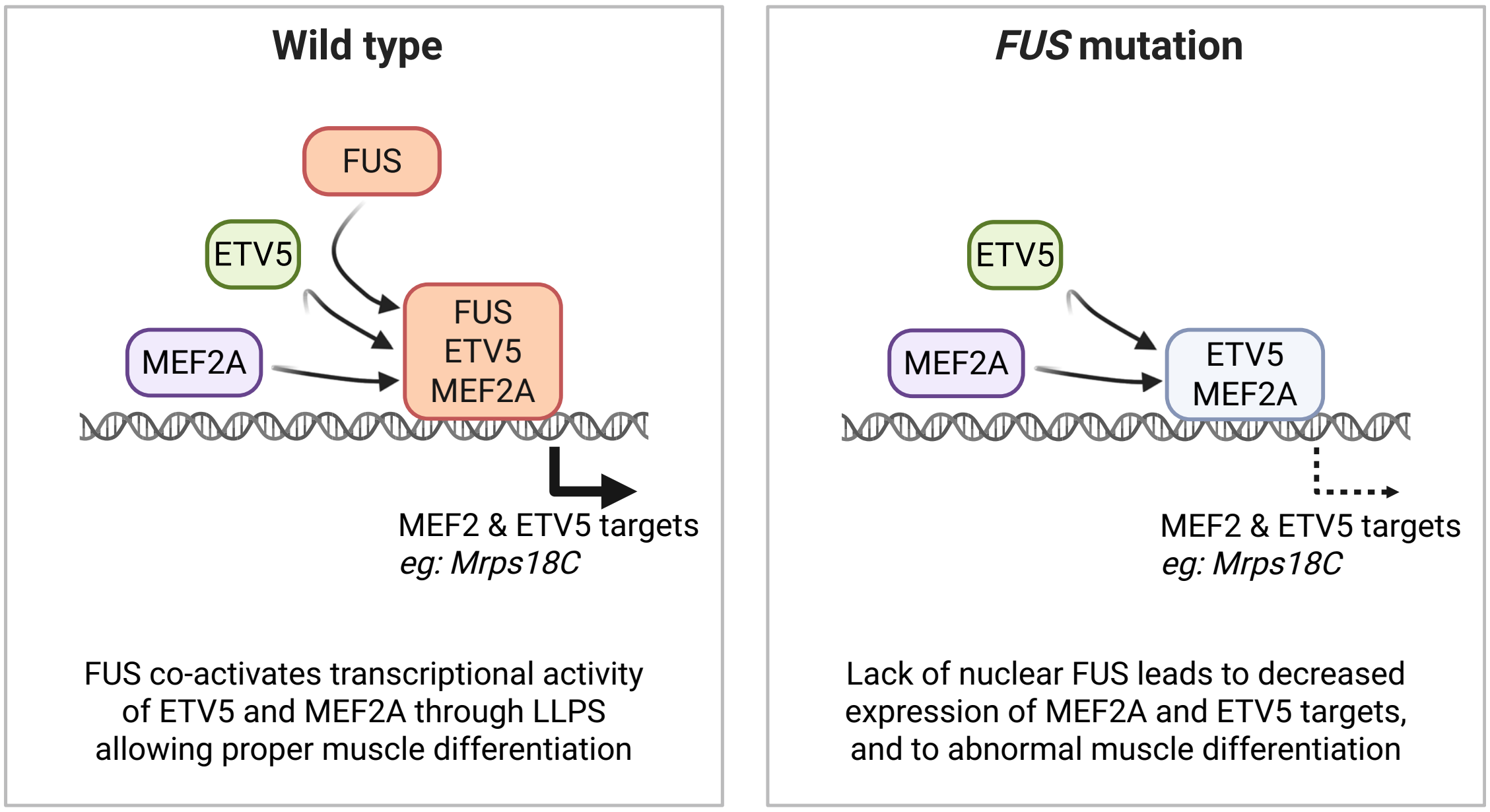


**Figure EV5. FUS phase separation properties are required for ETV5 and MEF2A to transactivate key muscle differentiation pathways.**

FUS mutations compromise muscle differentiation.

 © The Author(s)
