# Supplementary material for: FUS controls muscle differentiation through phase separation-mediated recruitment of the transcription factors MEF2 and ETV5
Source: EMBO J. 2026 Jul 22;45(17):6267–98. doi: 10.1038/s44318-026-00874-1 (PMC13534636; doi:10.1038/s44318-026-00874-1)
Supplement: Supplementary file 2 [file 44318_2026_874_MOESM1_ESM.pdf]

Appendix for "FUS controls muscle differentiation through phase separation-mediated recruitment of the transcription factors MEF2 and ETV5"

Table of contents

Appendix Figure S1: abnormal ultrastructure and mitochondrial defects in *FUS*-ALS iPSC derived myotubes. 2

Appendix Figure S2: qPCR validation of siRNA knockdown. 3

Appendix Figure S3: Expression levels and subcellular localization of FUS proteins used in this study 4

Appendix Figure S4: Normalized RNAseq counts for Etv1, 4 and 5 in various models. 5

Appendix Figure S5: Colocalization of FUS, ETV5 and MEF2A on FUS peaks. 6

Appendix Figure S6: Correlation between FUS, co-factor binding and gene expression fold change. 7

Appendix Figure S7: Additional EMSA specificity controls. 8

Appendix Figure S8: FUS, MEF2A and ETV5 LLPS predictions 9

Appendix Figure S9: subcellular localization of FUS, ETV5 and MEF2A proteins in transfected HEK293 cells 10

Appendix Figure S10: characterization of FUS, ETV5 and MEF2A condensates using STED imaging. 11

Appendix Table S1: muscle biopsies from ALS patients and controls 12

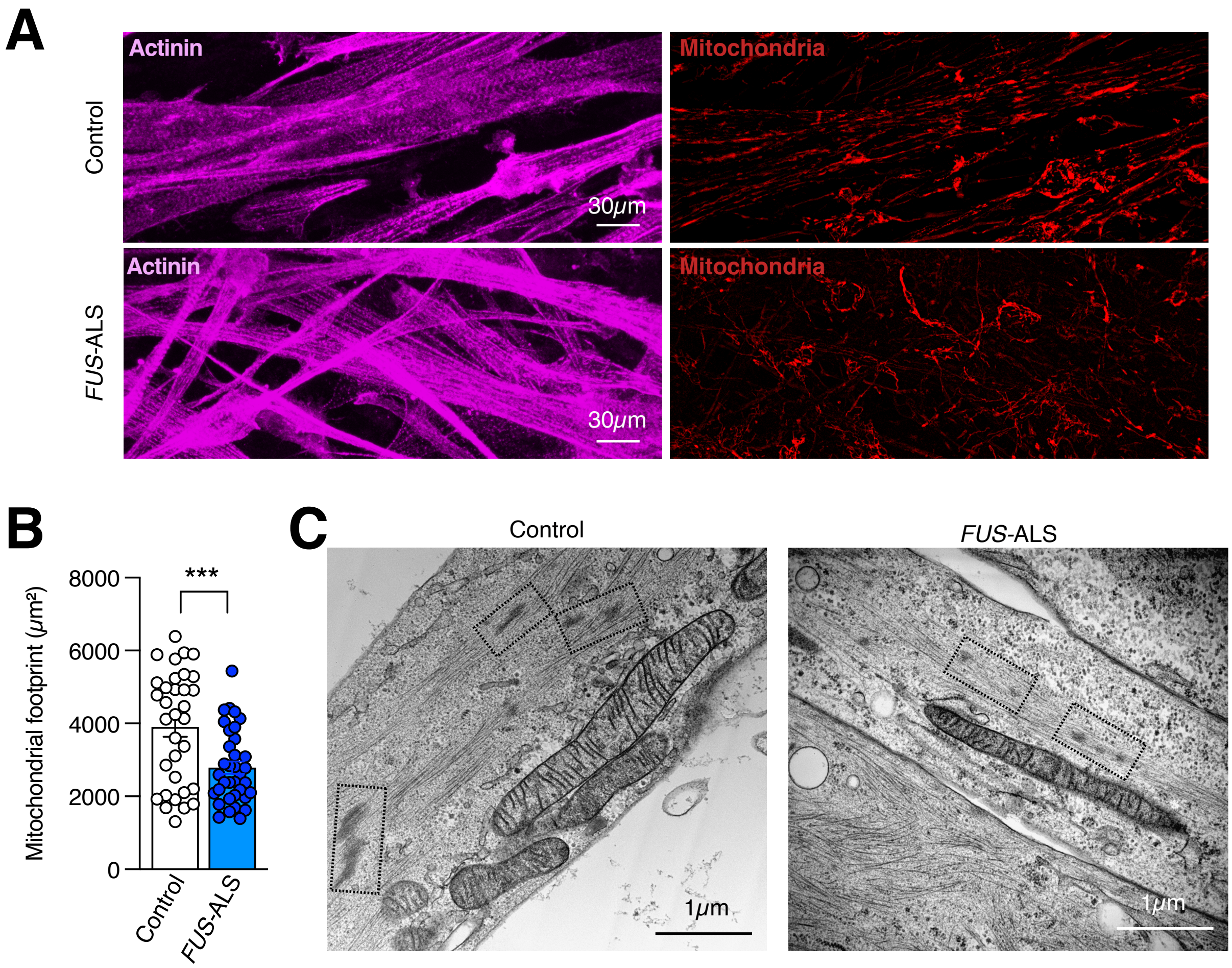

Appendix Figure S1: abnormal ultrastructure and mitochondrial defects in *FUS*-ALS iPSC derived myotubes.

A: Representative mitochondrial morphology in spheres-derived myotubes from iPSCs of a *FUS*-ALS patient (FUS1) or its isogenic control. Cells were stained with Actinin (magenta) and Mitotracker (red). Scale bars: 30 μm.

B: Quantification of mitochondrial footprint. Dots represent data from individual images obtained from 3 independent biological replicates. ***$p<0.001$, two-tailed unpaired t-test.

C: Abnormal ultrastructure in *FUS*-ALS iPSC-derived myotubes.

Ultrastructural alterations in iPSC-derived myotubes. Electron micrographs of sphere-derived myotubes from isogenic control and *FUS*-ALS patient iPSC lines, showing the mitochondria and Z-lines (dashed boxes). Z-lines in mutant myotubes appear less dense than in the control.

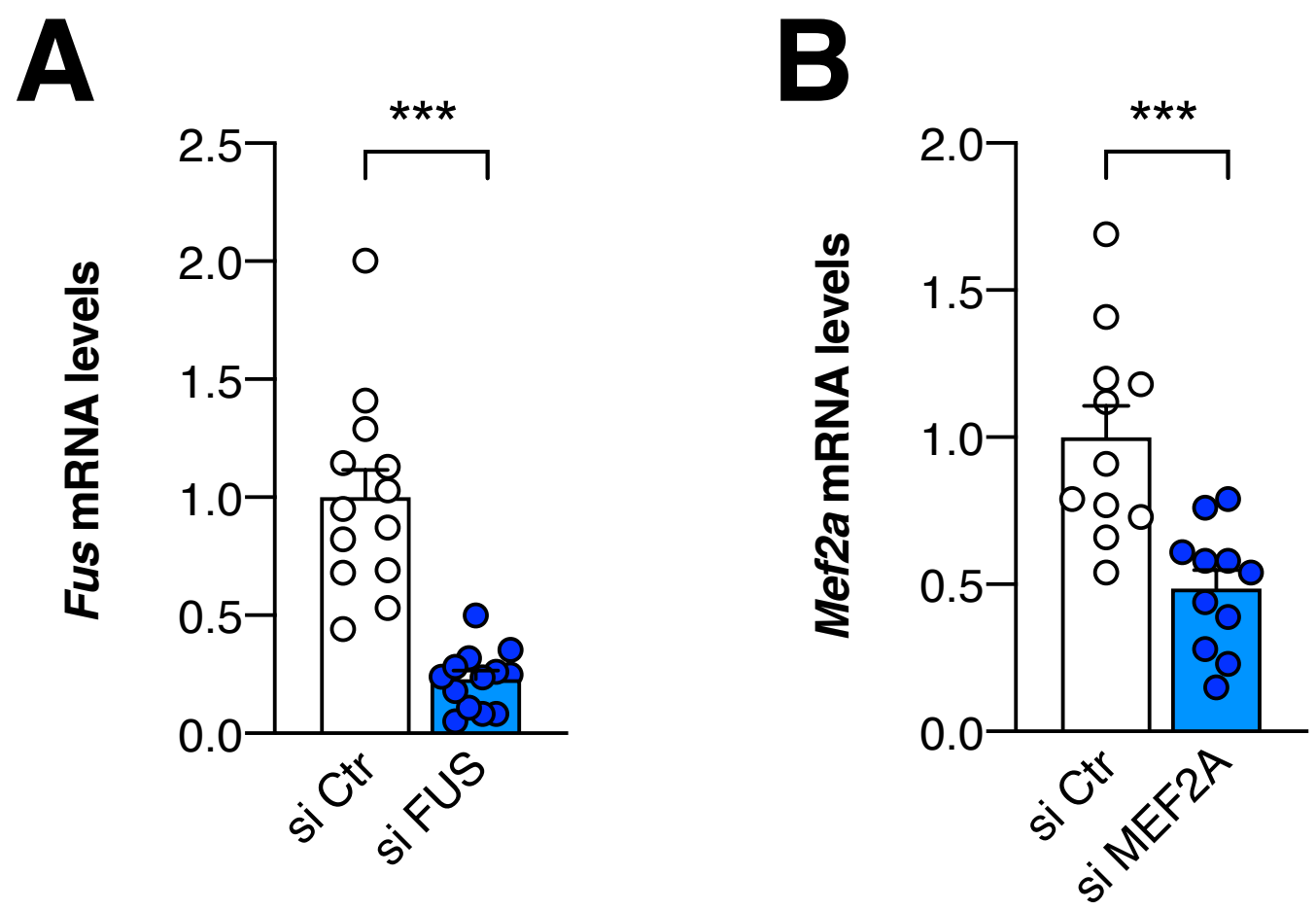

Appendix Figure S2: qPCR validation of siRNA knockdown.
a-b: C2C12 cells were transfected with siCtr, siFUS (a) or siMEF2A (b) and RNA was extracted 24h after transfection. *Fus* (a) or *Mef2a* (b) mRNA levels were measured using RT-qPCR. ***p<0.001 by Student's t-test.

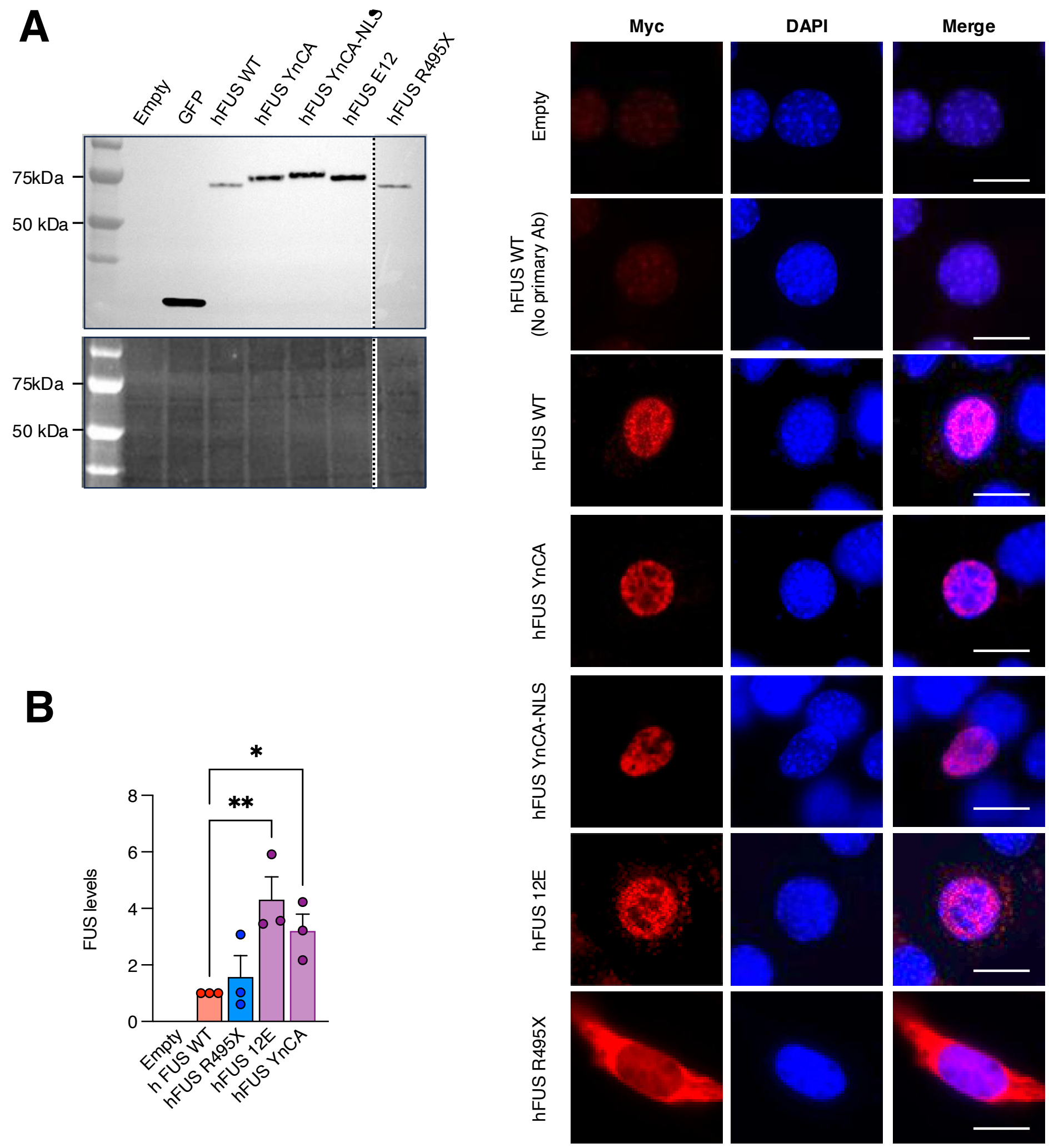

Appendix Figure S3: Expression levels and subcellular localization of FUS proteins used in this study

A-B: Representative western blotting of indicated constructs used in Figures 3, 4, 6 and 8 of the manuscript. All encoded proteins (including GFP) are N-terminally Myc-tagged, and an anti-myc antibody was used to visualize the transfected proteins. LLPS-deficient mutants included mutation of 7 tyrosine residues to alanine with (YnCA-NLS) or without an SV40 NLS (YnCA), or 12 phosphomimetic mutations (12E) that have previously been shown to lead to deficient LLPS. LLPS-deficient FUS proteins show a higher immunoreactivity and an increased apparent molecular weight in 3 independent experiments, as quantitated in panel B. Stainfree gel is shown in the bottom panel to indicate equal loading. Note that the original gel included 2 additional FUS constructs that were not used in the current study. These lanes were removed (indicated with the dashed line) to show only constructs relevant to this study. Full uncropped western blot and stainfree gel is included in expanded view data files provided.
One way anova, followed by Tukey, ** adjusted p=0.0040, *, adjusted p=0.028.
C: Representative immunofluorescence of FUS proteins used in this study. All FUS constructs except R495X, showed a nuclear localization. Note that the hFUS12E protein shows some cytoplasmic granular staining. Scale bar: 10μm.

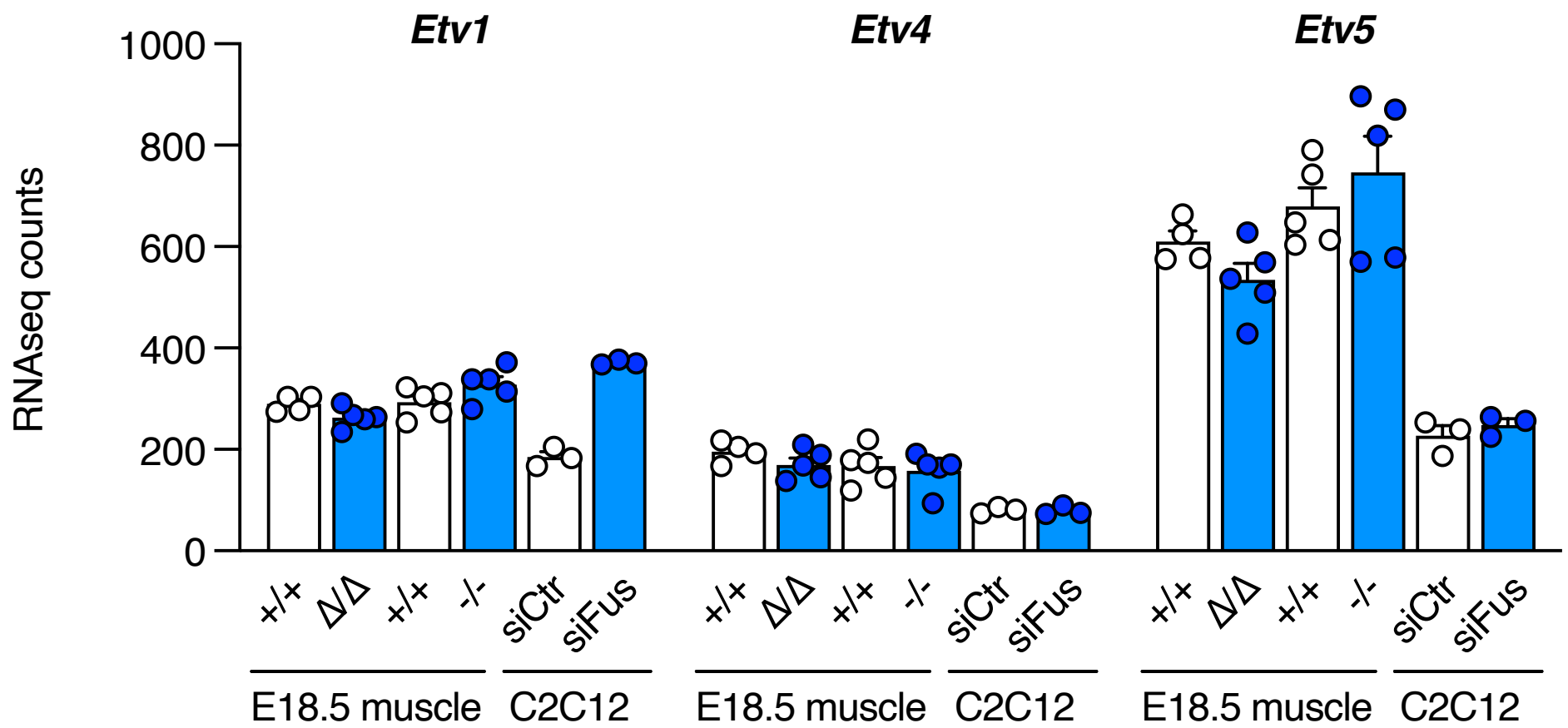

Appendix Figure S4: Normalized RNAseq counts for Etv1, 4 and 5 in various models.

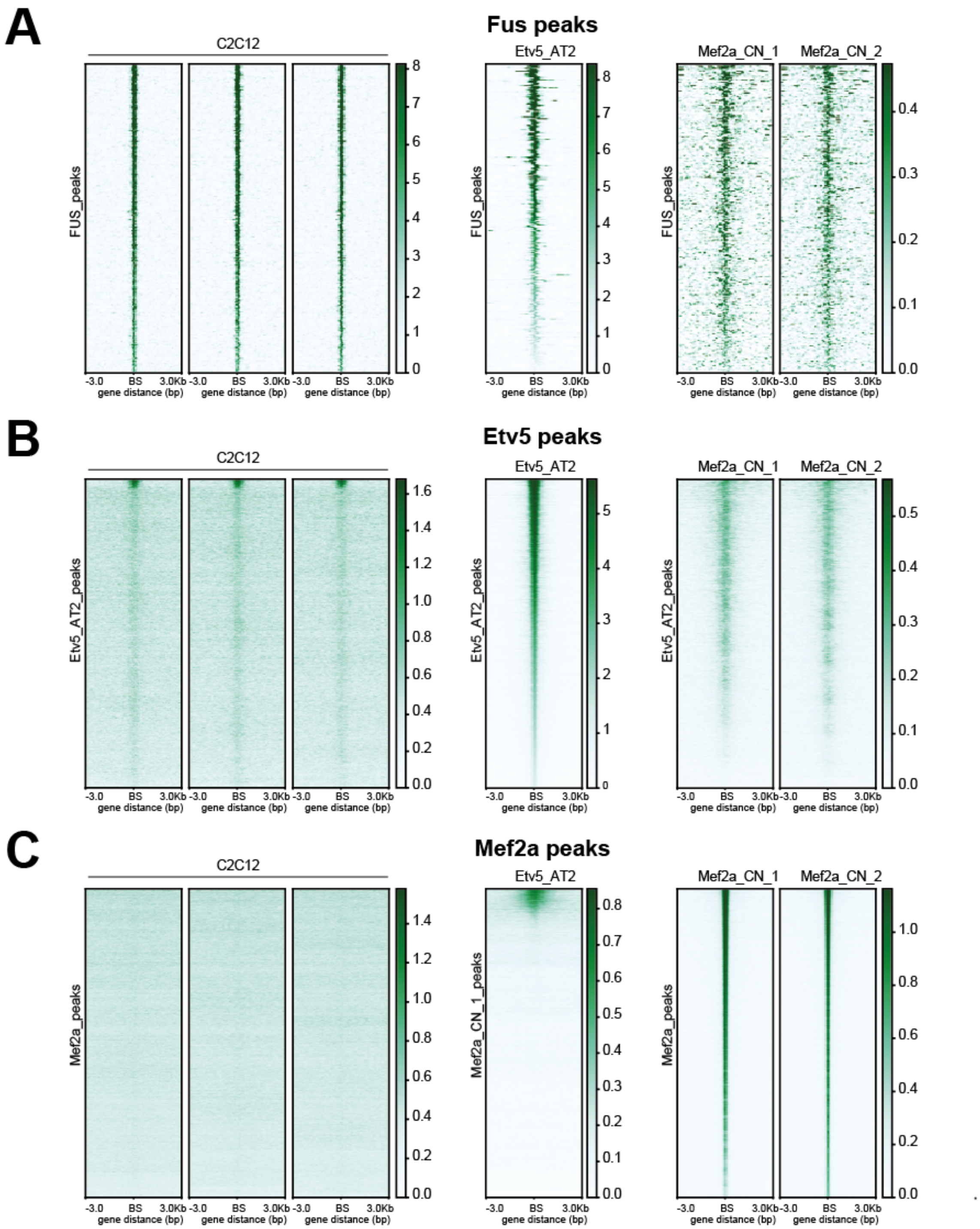

Appendix Figure S5: Colocalization of FUS, ETV5 and MEF2A on FUS peaks.
Heatmaps showing FUS (A), ETV5 (B) and MEF2A (C) peaks in the 3 replicates of C2C12 ChIP seq. Note colocalization of all three factors on FUS peaks only. ETV5 and MEF2A peaks were predicted using publicly available datasets in mouse AT2 cells and mouse cortical neurons (CN) respectively. References of the datasets are indicated in methods.

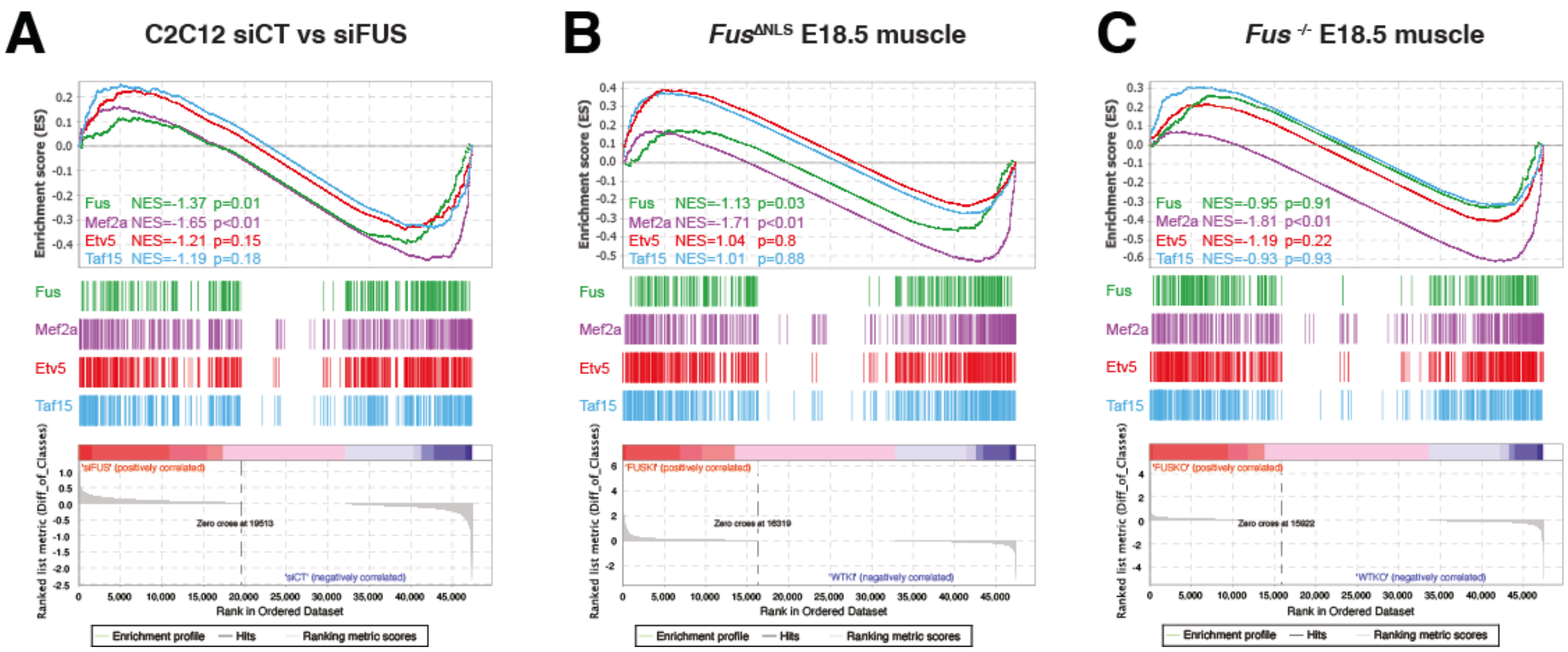

Appendix Figure S6: Correlation between FUS, co-factor binding and gene expression fold change.

Gene set enrichment analysis (GSEA) using genes closest to FUS (green), MEF2A (purple), ETV5 (red) and TAF15 (teal) peaks using gene expression data from C2C12 siFus vs siCT (A), E18.5 muscle $Fus^{\Delta NLS/\Delta NLS}$ vs $Fus^{+/+}$ (B), E18.5 muscle $Fus^{-/-}$ vs $Fus^{+/+}$ cells (C).

Note significant correlations for FUS in siFus vs siCT in C2C12 cells and E18.5 muscle from $Fus^{\Delta NLS/\Delta NLS}$ vs $Fus^{+/+}$ and for MEF2A for all three datasets.

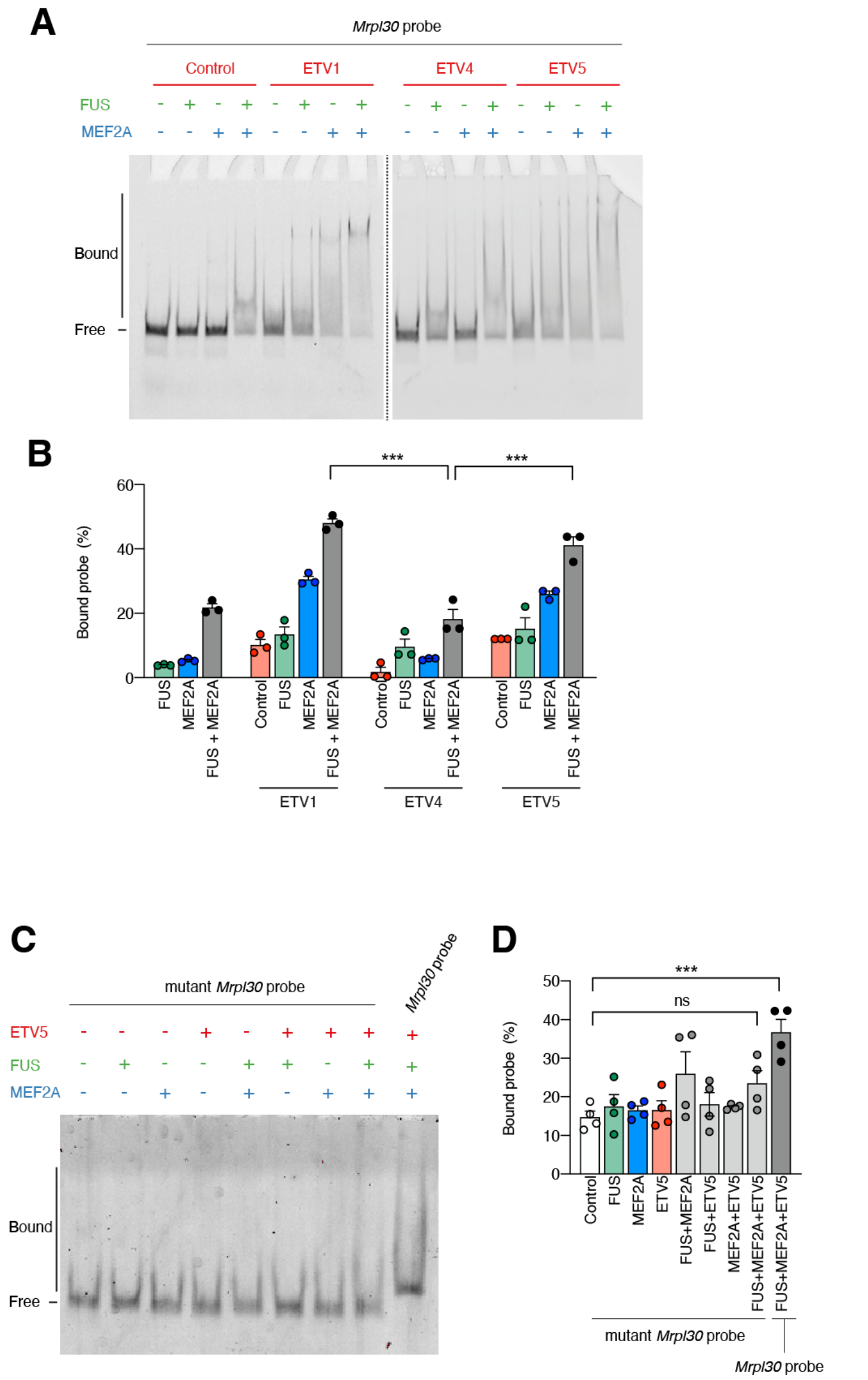

Appendix Figure S7: Additional EMSA specificity controls.

A: Cooperative binding of FUS, MEF2A and the different ETV factors (ETV1, ETV4 or ETV5) to a FAM labelled *Mrpl30* promoter-derived DNA probe. EMSA experiments were performed by using a constant concentration of FUS (300 nM), MEF2A (50 nM) and ETV (120 nM) and 5 nM of MRPL30 DNA probe.

B: Graph showing the percentage of *Mrpl30* probe bound. The intensity of the signals of the protein-DNA complex was quantified using the iBright™ Analysis Software. Error bars represent SEM of three independent experiments. ***$p<0.0001$ for ETV1+FUS+MEF2A vs ETV4+FUS+MEF2A and ETV5+FUS+MEF2A vs ETV4+FUS+MEF2A One way Anova followed by Tukey post hoc test.

C: EMSA experiments were performed with a constant concentration of FUS (300 nM), MEF2A (50 nM) and ETV5 (120 nM) and 5 nM of *Mrpl30 mutated* promoter-derived DNA probe where the ETV5 binding motifs have been mutated. As positive control, the last lane was performed with the non-mutated *Mrpl30* promoter-derived DNA probe.

D: Graph showing the percentage of *Mrpl30* mutated or control probe bound. The intensity of the signals of the protein-DNA complex was quantified using the iBright™ Analysis Software. Error bars represent SEM of four independent experiments. ***p = 0.0001 for control vs ETV5+FUS+MEF2A. One way Anova followed by Tukey post hoc test.

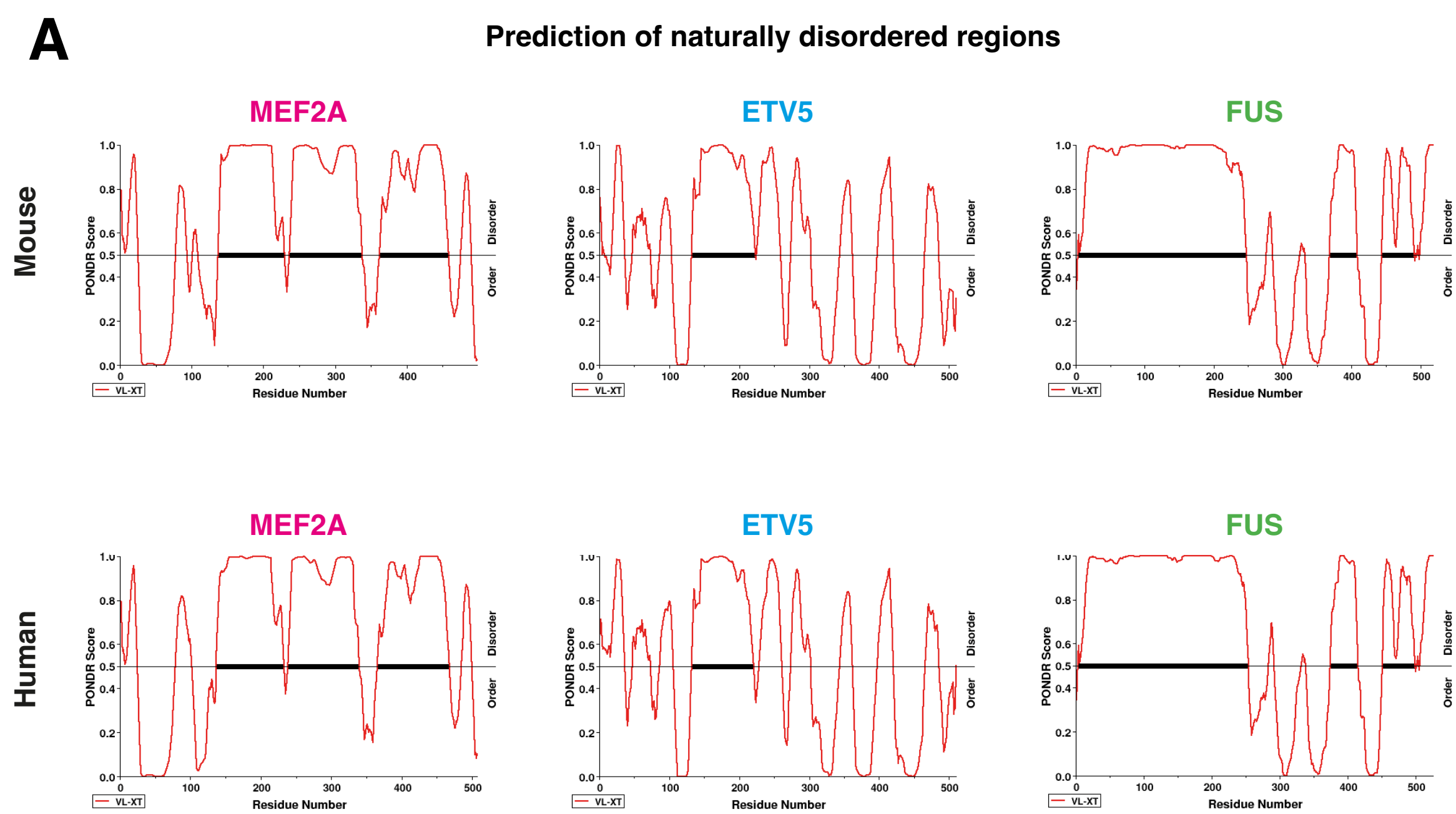

B **Prediction of phase separation behavior**

Protein	PSP Score	Phase separation prediction
Human FUS	0.9987	Yes
Mouse FUS	0.9987	Yes
Human MEF2A	0.969	Yes
Mouse MEF2A	0.9399	Yes
Human ETV5	0.8531	Yes
Mouse ETV5	0.7067	Yes

Appendix Figure S8: FUS, MEF2A and ETV5 LLPS predictions
A: Results of PONDR prediction for mouse and human MEF2A, ETV5 and FUS proteins. All 6 proteins display intrinsically disordered regions.
B: Prediction of phase separation behavior using PSP. All 6 proteins are predicted to undergo phase separation.

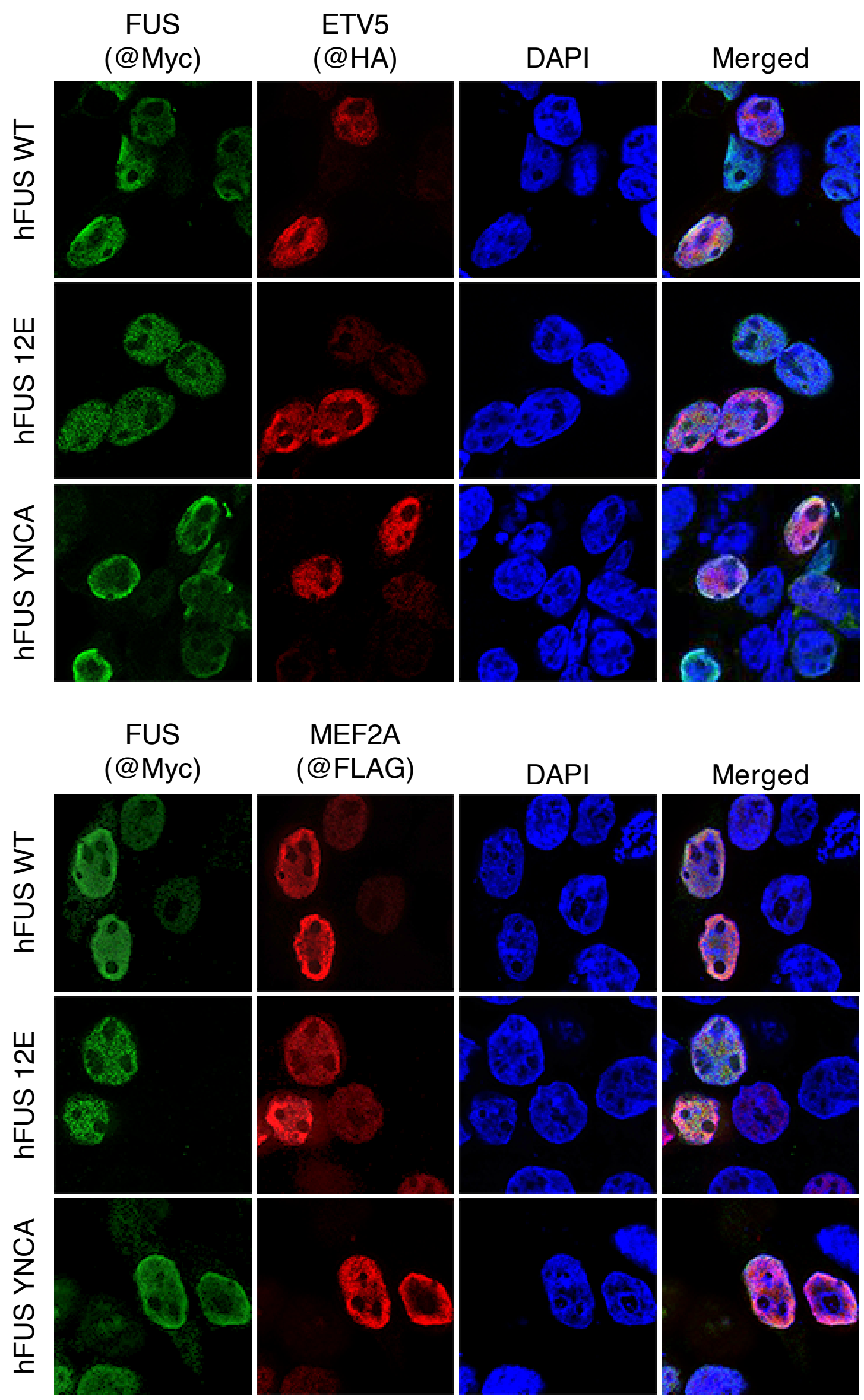

Appendix Figure S9: subcellular localization of FUS, ETV5 and MEF2A proteins in transfected HEK293 cells

HEK293 cells were transfected with indicated Myc-tagged FUS constructs and either HA-ETV5 (upper panel) or FLAG-MEF2A (lower panel). Cells were stained with anti-myc (green), anti-HA (upper panel, red) or anti-FLAG (lower panel, red). Shown are representative immunofluorescence images of the indicated proteins. Note that all constructs lead to primarily nuclear localization.

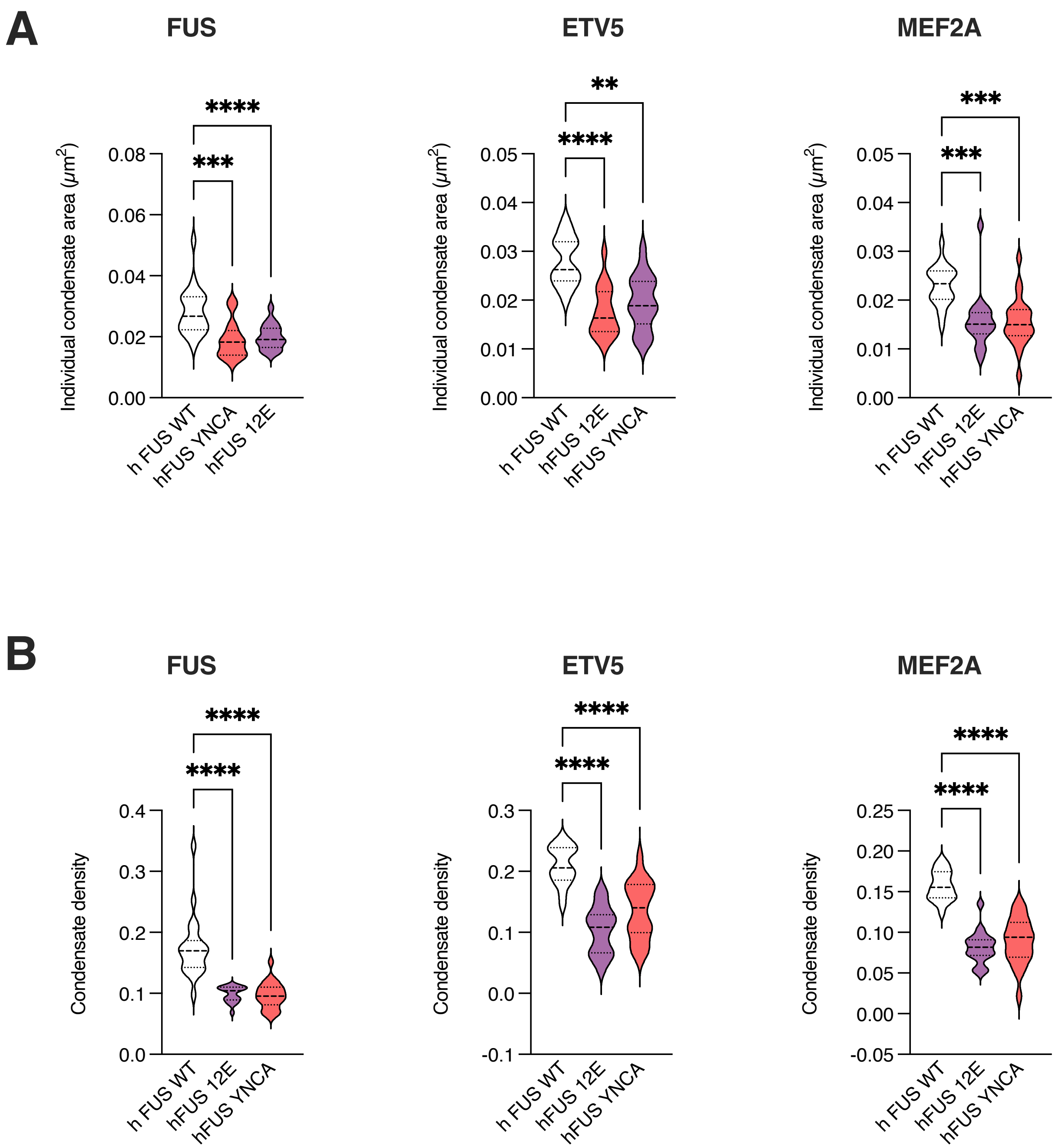

Appendix Figure S10: characterization of FUS, ETV5 and MEF2A condensates using STED imaging.

A-B: quantification of individual condensate size (μm², A) or density (fraction of occupied area, B) for the indicated condensates shown in Figure 8F-K.

Panel A: Mixed effects analysis. FUS: hFUS WT vs hFUS 12E: **** $p<0.0001$; hFUS WT vs hFUS YNCA: *** $p = 0.0001$; ETV5: hFUS WT vs hFUS 12E: **** $p<0.0001$; hFUS WT vs hFUS YNCA: ** $p = 0.0012$; MEF2A: hFUS WT vs hFUS 12E: *** $p = 0.0001$; hFUS WT vs hFUS YNCA: *** $p = 0.0001$;

Panel B: Mixed effects analysis, all comparisons indicated:****, $p<0.0001$.

Appendix Table S1: muscle biopsies from ALS patients and controls

Name	Gender, Age*	*FUS* mutation	Electromyography
CNTL1	Female, (33)	Not relevant	N/A
CNTL2	Male, (30)	Not relevant	N/A
FUS1	Male, 26 (26)	R495QfsX527	Intensive PSA, neurogenic and myogenic MUAP
FUS2	Female, 24 (27)	K510R	Intensive PSA, neurogenic MUAP
FUS3	Male, 39 (42)	K510R	Moderate PSA, myogenic and far less neurogenic MUAP
FUS4	Male, 24 (24)	G478L	Moderate PSA, neurogenic and myogenic MUAP
CNTL3	Female (65)	Not relevant	M.vast. lat. right: No pathological spontaneous activity. Small, polyphasic MUAPs. Early recruitment of MUAPs. EMG indicating myopathy.
CNTL4	Female (63)	Not relevant	M. vast. lat.left: normal.
CTNL5	Female (68)	Not relevant	M. vast. lat.left: normal.
Sporadic ALS 1	Male (57)	-	M. vastus lat. right: No pathological spontaneous activity. MUAPs normal. Reduced interference pattern. M. tib. ant. right: Pathological spontaneous activity in form of positive sharp waves (++) and fasciculations (++). Large and polyphasic MUAPs. Reduced interference pattern. EMG indicating neuropathy.
Sporadic ALS 2	Female (65)	-	M. vastus med. right: Pathological spontaneous activity in form of positive sharps waves (+), fasciculations (+) and complex repetitive discharges (+++). Large MUAPs. EMG indicating neuropathy.
Sporadic ALS 3	Male (65)	-	M. biceps brachii left: Spontaneous activity in forms of fibrillations (+), positive sharp waves (++) and fasciculations (++). Large MUAPs. EMG indicating neuropathy.

*Age of onset (age at biopsy); CNTL: Control, PSA: pathological spontaneous activity; MUAP: motor unit action potential
