# Supplementary material for: FUS controls muscle differentiation through phase separation-mediated recruitment of the transcription factors MEF2 and ETV5
Source: EMBO J. 2026 Jul 22;45(17):6267–98. doi: 10.1038/s44318-026-00874-1 (PMC13534636; doi:10.1038/s44318-026-00874-1)
Supplement: Supplementary file 3 [file 44318_2026_874_MOESM2_ESM.pdf]

THE EMBO JOURNAL

# FUS controls muscle differentiation through phase separation-mediated recruitment of the transcription factors MEF2 and ETV5

Gina Picchiarelli, Anne Wienand, Salim MEGAT, Saskia Hutten, Amr Aly, Marije Been, Isabelle Weber, Angela Bonanno, Nibha Mishra, Erin Sternburg, Pierre Cauchy, Stephane Dieterlé, Marica Cattinozzi, Javier Lobo-Mancheno, Hannah Walgrave, Valérie Demais, Pierre Hener, Pascal Kessler, Laura TZEPLAEFF, Anne Hubers, Dagmar Zeuschner, Angela ROSENBOHM, Albert Ludolph, Anne-Laurence BOUTILLIER, Tobias Boeckers, Dorothee Dormann, Maria Demestre, Chantal Sellier, Clotilde Lagier-Tourenne, Erik Storkebaum, and Luc DUPUIS

*Corresponding author(s): Luc DUPUIS (ldupuis@unistra.fr) , Erik Storkebaum (erik.storkebaum@donders.ru.nl), Clotilde Lagier-Tourenne (clagier-tourenne@mgh.harvard.edu), Chantal Sellier (chantal.sellier@inserm.fr)*

## Review Timeline:

| | |
|---|---|
| Submission Date: | 5th Nov 25 |
| Editorial Decision: | 16th Jan 26 |
| Revision Received: | 11th May 26 |
| Editorial Decision: | 22nd Jun 26 |
| Revision Received: | 25th Jun 26 |
| Accepted: | 26th Jun 26 |

*Editor: Ioannis Papaioannou*

## Transaction Report:

(Note: With the exception of the correction of typographical or spelling errors that could be a source of ambiguity, letters and reports are not edited. Depending on transfer agreements, referee reports obtained elsewhere may or may not be included in this compilation. Referee reports are anonymous unless the Referee chooses to sign their reports.)

This manuscript was transferred to The EMBO JOURNAL following peer review at another journal.

**Reviewers' comments:**

**Reviewer #1 (Remarks to the Author):**

The study by Dr Dupuis & co-workers describes experiments supporting FUS protein contribution to the normal muscle functioning and muscle development, with implication for ALS caused by FUS mutations. Strengths of the study are: i) use of multiple models of FUS pathology – iPSCs, flies, human tissue and transgenic mice; ii) use of several techniques spanning from in vitro/recombinant protein, to mouse behaviour and survival tests, to human postmortem tissue analysis; iii) novel transactivation targets for FUS important for muscle homeostasis are identified. Several experiments are nicely done, and data looks robust (e.g. transcriptomic analyses, Drosophila genetics experiments).

However I have two major concerns: i) lack of QC data for several important experiments (see below) and ii) that a central conclusion of the study (LLPS dependence of cellular mechanisms) is not backed by sufficient/substantial data. Indeed, LLPS dependence of FUS regulation of MEF2 and ETV5 referred to in the manuscript title relies on a single cellular experiment.

We thank the reviewer for their positive opinion on our study. We have substantially revised of our manuscript, with multiple additional data to strengthen our major conclusions, as explained in more detail below.

Specific comments

1. FUS KO/KD and NLS mutants are used together or interchangeably in several parts, which is difficult to reconcile with the previous papers from the same group on mutant FUS gain of function in the cytoplasm as the primary mechanism of toxicity.

We acknowledge this point. As stated by the reviewer, mutant FUS gain of function appears critical in the CNS. However, in the skeletal muscle, NLS mutation appears to lead to a partial loss of function of FUS. This can be due to the syncytial nature of muscle cells, or other cell-specific mechanisms. We made this point clearer in the introduction (lines 98-104) and added a section on this topic in the discussion section (lines 577-586).

2. Shifts between model systems and phenotypes are impacting the manuscript readability. For example, murine experiments show a defect in muscle development when FUS is mutated, and in the next figure, fly experiment are done to demonstrate a role in the adult muscle.

It is correct that in the current manuscript, we provide novel data to show that *Drosophila caz* function in myocytes is required for maintenance of adult muscle integrity. However, as mentioned in the second sentence of the relevant paragraph in the results section, we previously showed that *caz* is required for proper muscle development in *Drosophila* (Catinozzi et al, Plos Genet, 2020). This is consistent with our finding that *Fus* mutation or deletion in mice lead to muscle-autonomous defects (Figure 2).

In fact, we believe that the main added value of the new *Drosophila* data presented in Figure 3 is that they show that *caz* is not only required for proper muscle development by promoting founder myoblast selection (Catinozzi, Plos Genet, 2020), but that *caz* is also required in myocytes for maintenance of adult muscle integrity. Indeed, we show that selective inactivation of *caz* in differentiating myocytes, after myoblast selection and myoblast fusion occurred, resulted in a reduced number and occasional misorientation of adult dorsal abdominal muscles (Figure 3). This defect in adult muscle integrity had deleterious functional consequences, as illustrated by an adult eclosion defect, a substantial motor performance deficit, and a reduced life span (Figure 3).
We rephrased sentences in this section to make this point clearer (lines 247-249).

3. Muscles from ALS-FUS patients were analysed and compared to healthy, intact muscle. Striking ultrastructural abnormalities detected are likely due to the muscle severely degenerated and are therefore readily expected. Samples from other ALS subtypes, to establish FUS-specific differences, may be used, but I suspect the pattern of changes will be similar. In this instance, studies in cellular models cannot be used "to exclude that … these defects are secondary to muscle denervation".

To address this point, we performed TEM in muscles of 3 sporadic ALS patients and 3 additional age-matched controls (as sporadic ALS patients are older than *FUS*-ALS patients). As predicted by the reviewer, we observed a similar phenotype. It should be noted however that Z-disk abnormalities were more frequent in controls of sporadic ALS patients (mean age 62 years) as compared to controls of *FUS*-ALS patients (mean age 32 years), which suggests an age effect on muscle ultrastructure. The ultrastructure of *FUS*-ALS muscles appeared also qualitatively more affected than sporadic ALS patients (see representative images), which was difficult to quantify objectively. We added these novel results in a supplementary figure 2 (lines 135-142).

The reviewer argues that cellular models cannot be used to exclude that defects are secondary to muscle denervation, and they are right. However, these models show that mitochondrial defects are, at least partially, cell autonomous. This is further confirmed by our TEM analysis of NLS mutant mice with rescued FUS localization in muscle, which shows a rescue in the muscle ultrastructural defects (Figure 2), and our experiments in drosophila showing that adult muscle maintenance is dependent on Caz expression in myocytes (Figure 3).

Thus, while it is likely that the ultrastructural muscle defects in *FUS*-ALS patients is in part secondary to muscle denervation, our iPSC model as well as our genetic experiments in mouse and fly provide substantial evidence that muscle loss of FUS function (or expression of a FUS mutation) is sufficient to drive such defects. To avoid any misunderstanding, we rephrased the relevant sentences to claim a cell autonomous phenotype rather than to exclude the contribution of denervation (lines 143-144). We also cite recent large scale proteomic studies supporting the involvement of muscle damage in ALS.

4. Likewise, it is not indicated whether 22-mo old mice (Fig.2) had a significant paralysis phenotype – these findings would be expected if so. This type of analysis should be done in presympromatic animals (well before symptom onset) – if found in those, such deficiencies would be indicative of FUS as a cause.

These mice do not develop a significant paralysis phenotype (Scekic-Zahirovic et al., 2017), despite the muscle phenotype we observed. We agree that aging as well as ongoing motor neuron degeneration could be contributing to this phenotype in aged animals. Of note, even in these very old animals, the mitochondrial and ultrastructural defects induced by the heterozygous mutation in mice were not as severe as the ultrastructural muscle defects observed in *FUS*-ALS patients. This could be caused by species differences in muscle aging, and we now cite a recent review discussing this point (Dupuis and Robertson, 2025).

It should be noted however, our genetic rescue experiment in homozygous mice (Figure 2) demonstrates that mutant FUS expression in skeletal muscle causes the ultrastructural muscle defect by a cell autonomous mechanism.

To accommodate the reviewer's point, we toned down our claims in the relevant section of the manuscript.

5. Quantification is missing from Fig.1b

The aim of Figure 1b was mostly to confirm mitochondrial dysfunction suggested by decreased cytochrome c levels in Figure 1a, and imaging was not performed on sufficient tissue sections to allow for rigorous quantification. To answer the reviewer's point, we included a quantification of cytochrome c in figure 1a, for which we had sufficient imaging data. This is now presented per patient in the right panel of revised Figure 1a.

6. Figure 1e. Was the mitochondrial footprint normalised to account for differences in abundance of myotubes?

The mitochondrial footprint was not normalised for differences in myotube abundance. However, in this system, we did not observe obvious alterations of myogenic markers (eg *MYOG* mRNA levels) between control and *FUS*-ALS hiPSC-derived myotubes. This was described in Supplementary Figure 7 of our previous study (Picchiarelli et al., 2019).

7. Fig.4 and 5. QC data is missing for all luciferase reporter assays. What was used to verify equal efficiency of transfection if Renilla was not used? Further, expression level of R495Z mutant vs WT protein is not shown. How much is R495X mutant mislocalised? A gain of function may be involved, since mutant FUS is known to affect translation at several levels, therefore lack of rescue by R495X mutant could be attributed to the inhibition of luciferase expression. Many conclusions rely on the results from these reporter experiments.

We thank the reviewer for this comment.
All our reporter experiments with 3xMEF2 luciferase were normalized by protein amount. All our experiments with *Mrsp18C*-luciferase use secreted alkaline phosphatase (SEAP) as an internal control. SEAP cDNA is under the control of CMV promoter on the same plasmid (Gluc-ON kit from GeneCopoeia). Results with both normalisation methods are essentially similar, which increases confidence that our results are not confounded by improper normalisation of transfection.
In the revised manuscript, we now provide expression levels and representative immunofluorescence for all FUS mutants used in a novel supplementary Figure 6.

Regarding the possible effect of FUS on translation, we acknowledge that this is possible, however, we previously showed that FUS overexpression had minimal effect on CMV-driven expression of luciferase in our C2C12 system (figure 5h of (Picchiarelli et al., 2019)), which argues against this possibility. We further confirmed these results by re-plotting the CMV-SEAP results (normalisation of Figure 5h-k), and observed little effect of WT or mutant FUS expression on CMV driven SEAP expression (see figure below). The trend towards increased signal (10-15%) is consistent with our prior studies, and contrasts with the 3- to 10-fold increase observed when using either 3xMEF2 luciferase or Mrps18c-luciferase driven expression. Of note, FUS represses translation in the cytoplasm, which makes it very unlikely that our observed increased expression of the MEF2 reporter is due to direct translational effects of FUS (Birsa et al., 2021).

Thus, it is unlikely that our reporter experiments are flawed by an effect of FUS on translation. We modified the texts lines 300-303 to mention this possibility, and why we exclude it.

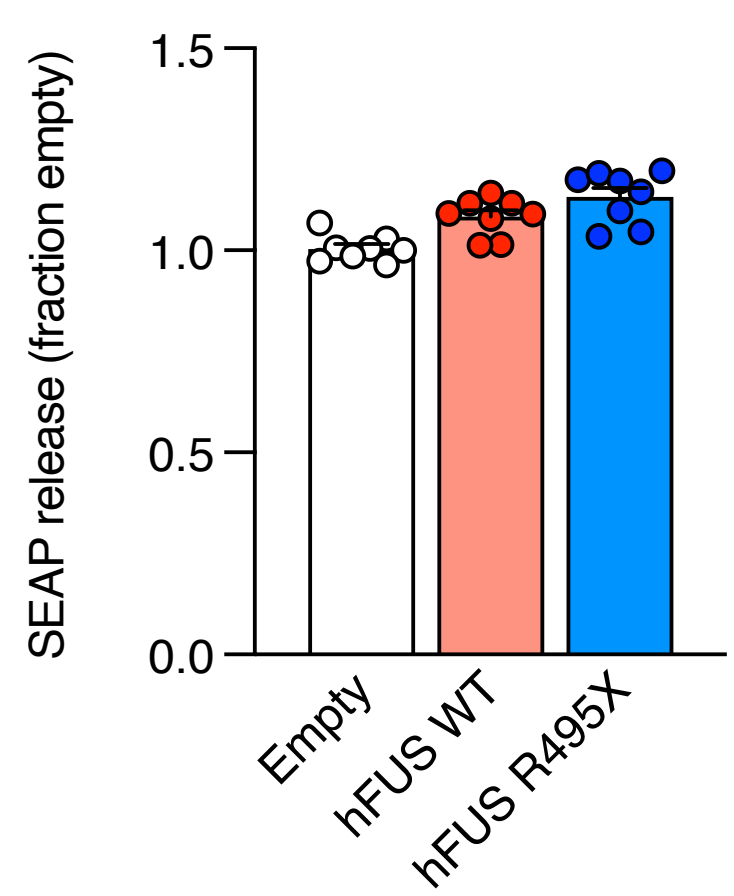


**Effect of wild type or R495X FUS on SEAP release in C2C12 cells.**

All experiments in Figure 5h-k include an internal control with secreted alkaline phosphatase, as measured in the culture medium according to the provider's instructions (GeneCopoeia). We plotted the results of SEAP release in n=8 of the experiments. p=0.135 (Empty vs hFUS WT).

8. Fig.5h-k. Why does ETV1/5 knockdown have no effect on the reporter expression, whereas ETV1 overexpression increases it?

The reviewer is right in noticing that individual knock down of PEA3 family members is not sufficient to decrease *Mrps18c* promoter activity despite the presence of several ETV binding sites in this promoter. Contrastingly, overexpression of ETV5 is sufficient to increase promoter activity. Our interpretation is that loss of function induced by siRNA knock down is either not sufficient or compensated by the other ETV members in knock down experiments, while overexpression allows to bypass such compensatory mechanisms. Of note, individual knock-down of ETV1 or ETV5 are sufficient to blunt the FUS-stimulated response, which would be in line with both ETV1 and 5 being necessary for FUS-mediated transactivation of this promoter (line 389-391).

9. Fig.8a,b. Confirmation of in situ interaction between FUS and MEF2A / ETV5 is required by PLA, including a decrease in interactions when FUS is mutated (to support R495X data).

To answer the reviewer's comment, we sought to perform PLA but this assay did not work in our hands for the constructs available. To circumvent this technical problem, we performed super-resolution imaging and advanced quantification methods to show the colocalization of FUS with either MEF2A or ETV5 in transfected cells. Most interestingly, this colocalisation was mostly abolished with LLPS-deficient FUS. These results support both the existence of interaction or very close proximity between these three proteins and the dependency of this interaction to LLPS properties of FUS. These results have been added as novel figure 9f-k (lines 487-508).

10. LLPS dependency of FUS effect in transactivation of these TFs is one of the main conclusions of the study (appearing in the title). Cellular experiments are required to prove this point. Localization of the proteins with high-resolution imaging should be shown – presence in the same puncta. Does FUS YnCA significantly mislocalise requiring SV40 NLS attachment and why is this the case if its NLS is not affected? FUS YnCA can have other functions compromised – other than ability to undergo LLPS, including PPI, etc. LLPS-deficient FUS with phosphomimetic substitutions should be tested as well (PMID 28790177).

We agree with the reviewer that one of our main conclusions in the initial manuscript was only relying on a FUS mutant with multiple mutations abolishing LLPS properties. As requested by the reviewer, we now tested the 12E mutant from the group of Pr Fawzi, described in the referenced study. We obtained identical result with reporter assays using this 12E mutant (novel figure 9e, lines 487-489) and also observed that this mutation strongly decreased colocalization between FUS and either ETV5 or MEF2A using STED imaging (novel figures 9f-k, lines 490-508). This provides two orthogonal proofs as requested by the reviewer and is now included in the revised manuscript. We further added a sentence on this topic in the discussion section (lines 633-635).

11. Fig.8E. MEF2A-mCherry enrichment in condensates looks inconclusive – the signal needed significant enhancement, generating high background.

We agree with the reviewer that the enrichment of MEF2A-mCherry seems low. This is due to the use of a red image and a low mCherry signal of the recombinant protein. To better highlight this enrichment, we converted the image in grey scales which better shows the existing enrichment. This is now included in the revised Figure 9.

Minor

1. Muscle type used is not indicated in the text for Fig.2.

We apologize that the muscle used (gastrocnemius) was only indicated in the methods section. It is now clearly stated in the results and figure legend.

2. Is there anything special about the datapoint in Fig4k that does not have black border?

We apologize for not noticing this presentation error due to the transfer between Graphpad Prism and Illustrator. This has been fixed in revised Figure 4.

3. Fig.5h-k. Some error bars are both directions and others are up only. In Figure legend, concentration of 25 nM could be indicated once for all siRNAs.
We apologize for not noticing this presentation error due to formatting in Graphpad Prism. This has been fixed in revised Figure 5, as well as in Figures 6 and 8 in which the same formatting issue was present.
The concentration has been indicated only once in the Figure legend, for this figure but also other figures which had similar presentation.

4. A control that does not partition into FUS condensates is required in Fig.8e.
This point is important yet is not easily addressed. Indeed, it is trivial that most proteins do not phase separate but experimental conditions and physiological conditions are important modifiers that significantly alter the LLPS properties of proteins.
To address the reviewer's question, we formed FUS-GFP condensates in the presence of recombinant mCherry. As shown below, the mCherry protein was not able to phase separate on its own or in FUS condensates.

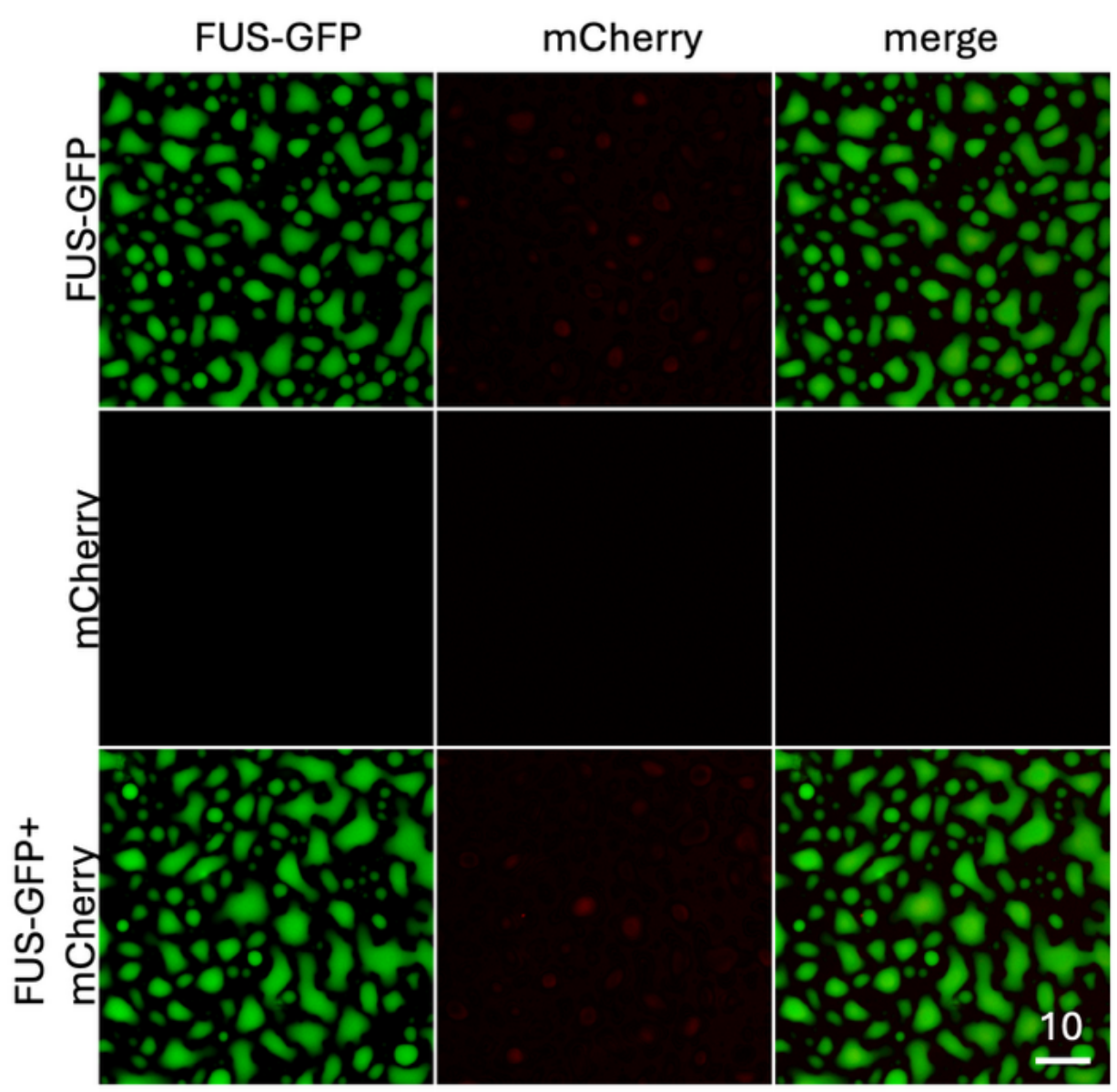


**Negative control for FUS co-partitioning**
Representative confocal images of FUS-EGFP condensates (5 $\mu$M) in absence or presence of mCherry upon protease-mediated cleavage of the MBP-solubility tags. No condensates were observed for mCherry alone and no enrichment in FUS condensates. Bar, 10 $\mu$m.

**Reviewer #2 (Remarks to the Author):**

I co-reviewed this manuscript with one of the reviewers who provided the listed reports.

**Reviewer #3 (Remarks to the Author):**

In this manuscript, PICCHIARELLI et al. report the role of FUS in muscle differentiation, a nuclear RNA-binding protein whose mutations are associated with amyotrophic lateral sclerosis (ALS). Previous studies reveal that knock-in mice of ΔNLS-FUS expressing a truncated form of FUS show cytoplasmic accumulation in the central nervous system and develop mild muscle weakness, and FUS is enriched in subsynaptic nuclei in skeletal muscles. How FUS expression contributes to neuromuscular demise in ALS remains poorly understood. In this study, the authors found that ALS-associated FUS mutations lead to ultrastructural defects in muscles of FUS-ALS patients, with disruption of sarcomeres and mitochondria. In mouse models along with RNA-seq analysis, loss or introduction of mutant FUS in myocytes leads to pronounced transcriptional defects, with a converging signature positioning MEF2 as a key disrupted transcription factor. Using ChIP-seq and luciferase reporter assay, they found FUS binds to the promoters of genes bound by ETS transcription factors in particular ETV5, and co-activates transcription of MEF2-dependent genes with ETV5. The authors hypothesize and demonstrate that FUS LLPS plays a critical role in recruiting MEF2A and ETV5 and is required for FUS-mediated activation of these transcription factors. Taken together, the authors propose that FUS is an essential protein for skeletal muscle structure through its phase separation-dependent recruitment of ETV5 and MEF2, defining a novel pathway compromised in FUS-ALS.

The data in this study are obtained mostly with quantitative and statistical evaluations and are generally supportive of the conclusions. The findings are overall informative to the field, and potentially very interesting, which may provide new insights into the pathogenesis of FUS-mediated ALS. However, the authors have only examined FUS knock-out or DNLS knock-in mice but not the real ALS mutation located in the NLS region of FUS, which may not recapitulate the real situation in FUS-ALS cases. Therefore, the current manuscript would be better suited to a more specialized journal.

We are very surprised with this reviewer's comment, as it seems to result from a misunderstanding of ΔNLS mouse model.
*Fus*$^{\Delta NLS/+}$ mice have been described in 2016 and 2017, and express a mutation truncating the nuclear localization sequence. Genetically, these mice express a G507X mutation, leading to truncation of all C-terminal amino-acids, including the whole NLS.
Importantly, this truncation is very similar to multiple ALS mutations found in FUS-ALS families, many of them being shown to be pathogenic. Illustrating this, we provide to the reviewers the figure of a recent review in Lancet neurology by Van den Bosch and colleagues (Moens et al., 2025), showing all FUS mutations identified to date (in 2025) in *FUS*-ALS families. We have indicated the truncating mutations identical or similar to the mutation in our FusΔNLS/+ mice with a red square. These authors indicate the existence of frequent *FUS* «mutations including premature stop codon variant Arg495*, which truncates the FUS protein after amino acid 494; Gln519Ilefs*9, a 4 bp deletion that results in frameshift and incorrect translation of the PY-NLS of the FUS protein; and Gly466Valfs*14, a splice-site mutation that prevents the correct splicing of *FUS* exon 14 and results in a frameshift».
Of note, multiple other mouse models generated by the groups of Pr Fratta or Pr Shneider have been generated with the same strategy of frameshifting the NLS of FUS (Devoy et al., 2017; Korobeynikov et al., 2022).
All these mutations are similar to our ΔNLS mutation, that leads to frameshift and no NLS translated. Thus, the FusΔNLS/+ mice express a «real ALS mutation», as far as can be done in a mouse model. In addition, we also studied extensively in our study the R495X (aka Arg495*)mutation in the human FUS cDNA, and found similar results, and three out of the four *FUS* -ALS patients studied in Figures 1 and S1 carry a point mutation (K510R and G478L).
To take into account this reviewer's comment, we rewrote introduction and presentation of the mouse models to take into account the similarity of the ΔNLS mutation to many pathogenic mutations found in patients. We used the same color code throughout the manuscript to indicate when we are studying a frameshift mutation (blue, either in patients or in mouse) or a point mutation (green in patients in Figure 1 and S1). We hope this clarifies the relevance of our work for this reviewer.

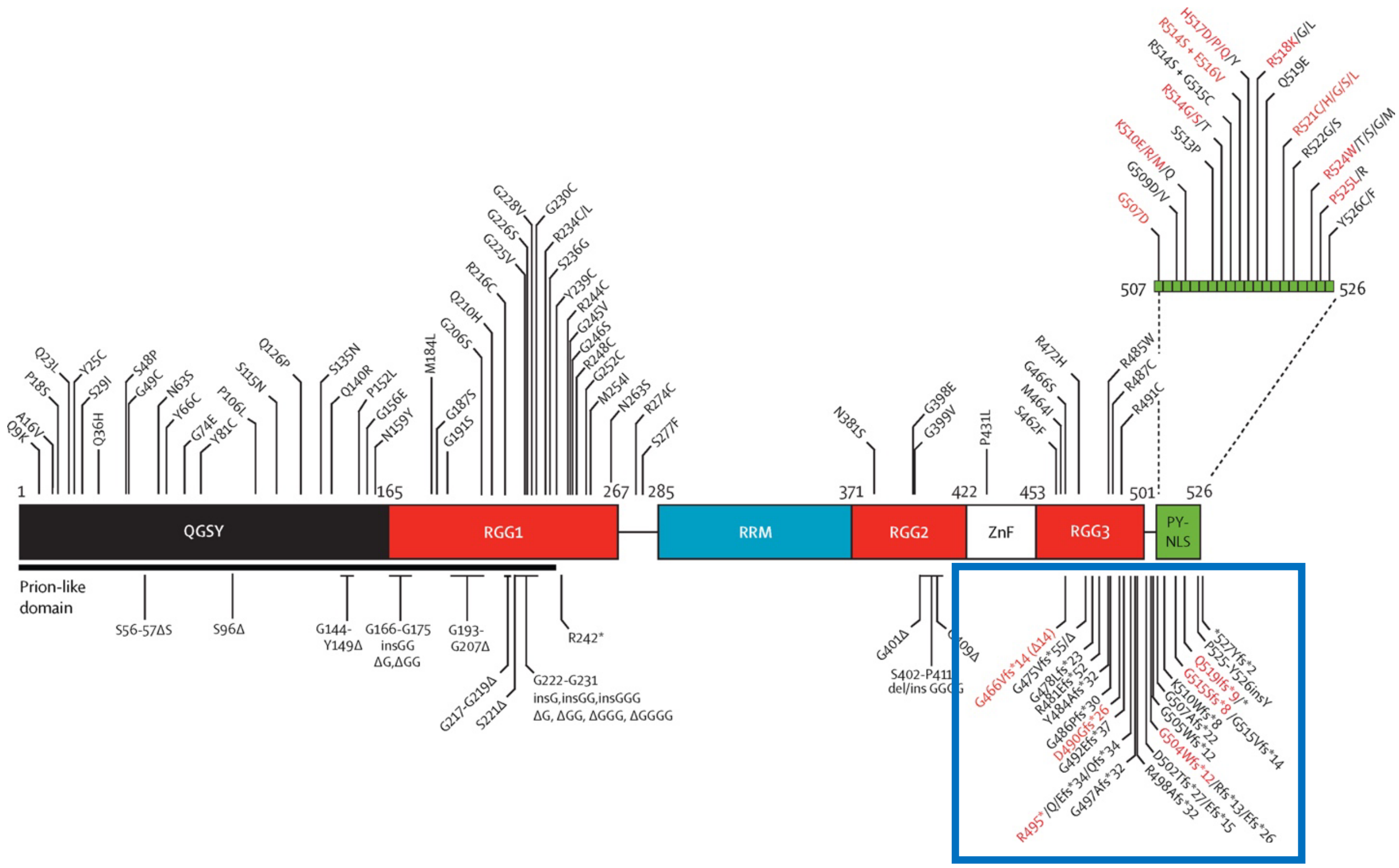


[Figure modified from Moens et al, Lancet Neurology 2025.](#)
All the mutations in the blue rectangle are frameshift mutations, deleting the C-terminal NLS of FUS. These frameshift are located between amino-acids 466 and 526 in ALS families, and our ΔNLS mouse model mutation is G507X, in the exact same region of the hotspot of frameshift mutations.

Major points

1. Many genetic mutations of FUS have been identified in ALS cases, especially in the NLS region (a.a. 510-526), such as K510R, R524S and P525L. In this study, the authors have examined FUS knock-out and ΔNLS knock-in mice, caz (i.e. FUS and 2 FET family proteins) knock-out flies, and ALS-linked R495X mutant FUS in C2C12 cells which is not located in NLS (a.a. 510-526). Non ALS-associated mutation in the NLS region has been evaluated in this manuscript. To make the result consistent, it would be better to select an ALS mutation in the NLS region and examine its effect on the role of FUS in muscle differentiation.

We agree with the reviewer that a fraction of ALS cases caused by FUS mutations are not caused by frameshifting or deleting the PY-NLS of FUS. However, as detailed above, a substantial fraction of the FUS mutations are indeed truncating the NLS through frameshifting (see above figure, from (Moens et al., 2025)). Moreover, these mutations are more severe (eg the R495X), and have been substantially documented as pathogenic through either disease co-segregation or trio analysis. Our analysis of the ΔNLS mice is also substantiated by similar results obtained in two K510R patients and 1 G478L patient (ALS cases 2, 3 and 4 analyzed in Figure 1), as well as our cell biology results obtained with the FUS(R495X) mutation (reporter assays on figure 4, 5 and 8). We thus have already selected several ALS mutations affecting the NLS (either through mutations or through truncation).

We acknowedge that more detailed explanations would have eased the reading of the manuscript and better highlighted the relevance of our experimental choices. To address this point, we rephrased our introduction section in lines 87-90. To make this point clearer, we used a color code in Figure 1 to indicate frameshift

mutations (ΔNLS, in blue) or point mutations (in green), and indicated the exact mutations studied in these patients in each of the panels and figure legends. Please note that this information was previously only provided in Table S1, which might have been missed by readers.

2. In RNA-seq analysis, the authors used gastrocnemius muscles of E18.5 FUS-/- and FUSΔNLS/ΔNLS mice and C2C12 cells with si-RNA mediated KD of FUS. It would be better to try the iPSC line from at least a FUS-ALS patient used in Figure 1 or C2C12 cells with FUS KO by CRISPR/Cas 9. We agree with the reviewer that adding another layer of transcriptomics could provide additional information. In the current manuscript, we already generated multiple datasets of RNAseq and Chip-seq in two knock-out and knock-in mouse models and one knock down cell model. The generation of a transcriptome of FUS iPS derived myotubes and Crispr/Cas9 C2C12 model could be of interest, but, in our opinion, goes beyond the current study, and add multiple layers of complexity in bioinformatic analyses while our current study is already using these approaches extensively.

3. In the fly experiment described in Figure 3, the authors should examine whether the expression of Wt-FUS or mutant FUS on the background of caz KO can rescue the phenotype of muscle defect caused by KO.

This is in principle an excellent suggestion, because this experiment would establish (i) whether hFUS and Caz are functional homologs in their role in adult muscle maintenance, and (ii) whether ALS-mutant FUS variants have LOF characteristics with respect to adult muscle maintenance.
However, many papers have shown that expression of either WT or ALS-mutant FUS in Drosophila induces dramatic phenotypes by itself (Chen et al., 2011; Jackel et al., 2015; Lanson et al., 2011; Machamer et al., 2014; Matsumoto et al., 2018; Miguel et al., 2012; Steyaert et al., 2018; Xia et al., 2012), including in muscle (Altanbyek et al., 2016). Consistent with these findings, we found that expression of WT or ALS-mutant hFUS in Drosophila myocytes (MHC-GAL4) induces developmental lethality. Because these muscle phenotypes induced by expression of hFUS alone would confound the interpretations of the results of the proposed experiment, we decided not to do this experiment.

4. The pathological relevance of LLPS on FUS-mediated activation of the transcription factors is worth evaluating since the LLPS properties of FUS have been extensively studied. Thus, the authors should try to select an ALS mutation that is easier to LLPS and examine whether this mutation can promote FUS-mediated activation of 3xMEF2-luc reporter activity.

We are not entirely sure of what was meant by the reviewer with this last major point. In the original manuscript, we used two LLPS deficient FUS variants based on mutations of tyrosine to alanine in the LLPS core to show that LLPS properties were required for FUS to mediate transactivation of MEF2 and ETV5 target genes.

To our knowledge, LLPS properties of FUS ALS mutants are either unchanged or decreased. Birsa et al showed a slightly decreased LLPS property of their Δ14 mutant (similar to our ΔNLS mutant), and unchanged properties of P525L mutant (Birsa et al., 2021). Rhine and collaborators (Mol Cell 2021) showed heterogenous variations in LLPS properties of mutant FUS according to the mutation, and generally less dynamic RNA interaction and decreased fluidity of mutants.(Rhine et al., 2020). Finally Murakami et al showed that FUS mutants in the low complexity domains led to less reversible FUS hydrogels, suggesting decreased liquid properties (Murakami et al., 2015). To our knowledge, there is no widely used FUS mutation « easier to LLPS » than the wild type protein, and the mutations generally alter the liquid behavior of the protein.

In response to this comment, we sought to strengthen our conclusion on LLPS dependency of FUS transcriptional activation of MEF2A/ETV5 pathway. To this aim, we used an orthogonal strategy to abolish LLPS in an otherwise wild type FUS. We tested the 12E mutant from the group of Pr Fawzi (Monahan et al.,

2017). This (non-ALS linked) mutant is a phosphomimetic variant of wild type FUS that virtually abolishes its LLPS proterties. We obtained identical result with reporter assays using this 12E mutant (novel figure 9e), and also observed that this mutation abolished colocalization between FUS and either ETV5 or MEF2A using superresolution imaging (novel figure 9 f-k) . This provides two independent evidence as requested by the reviewer. We further added a sentence on this topic in the discussion section. We hope that these novel results will convince the reviewer that LLPS properties of FUS are required for transactivation of MEF2 target genes.

Minor point
1. In the first paragraph, the authors claimed “analysis of mitochondrial footprint indicated that FUS mutation leads to a significant decrease in mitochondrial network area in the FUS-ALS myotubes relative to control (Figure 1e)”. The authors should point out the exact mutations in the manuscript.

The details of the mutations were provided in Supplementary table 1 and in the corresponding figure legends.

2. Because the deletion of NLS on FUS has not been identified in the ALS cases, the authors should discuss why they examine the role of ΔNLS but not the ALS mutation in the NLS region.
As stated earlier, we do not understand why the reviewer states that no deletion of FUS NLS has been identified in ALS patients while this is a very well established cause of FUS-ALS (see figure above), and likely among the causes of the most severe forms of ALS. We made this point clearer in the manuscript, citing the relevant litterature on this topic.

**References:**


Altanbyek, V., Cha, S.J., Kang, G.U., Im, D.S., Lee, S., Kim, H.J., and Kim, K. (2016). Imbalance of mitochondrial dynamics in Drosophila models of amyotrophic lateral sclerosis. Biochem Biophys Res Commun *481*, 259-264.

Birsa, N., Ule, A.M., Garone, M.G., Tsang, B., Mattedi, F., Chong, P.A., Humphrey, J., Jarvis, S., Pisiren, M., Wilkins, O.G.*, et al.* (2021). FUS-ALS mutants alter FMRP phase separation equilibrium and impair protein translation. Sci Adv *7*.

Chen, Y., Yang, M., Deng, J., Chen, X., Ye, Y., Zhu, L., Liu, J., Ye, H., Shen, Y., Li, Y.*, et al.* (2011). Expression of human FUS protein in Drosophila leads to progressive neurodegeneration. Protein Cell *2*, 477-486.

Devoy, A., Kalmar, B., Stewart, M., Park, H., Burke, B., Noy, S.J., Redhead, Y., Humphrey, J., Lo, K., Jaeger, J.*, et al.* (2017). Humanized mutant FUS drives progressive motor neuron degeneration without aggregation in 'FUSDelta14' knockin mice. Brain *140*, 2797-2805.

Dupuis, L., and Robertson, J. (2025). Is amyotrophic lateral sclerosis less severe in mice than in humans? Curr Opin Neurol.

Jackel, S., Summerer, A.K., Thommes, C.M., Pan, X., Voigt, A., Schulz, J.B., Rasse, T.M., Dormann, D., Haass, C., and Kahle, P.J. (2015). Nuclear import factor transportin and arginine methyltransferase 1 modify FUS neurotoxicity in Drosophila. Neurobiol Dis *74*, 76-88.

Korobeynikov, V.A., Lyashchenko, A.K., Blanco-Redondo, B., Jafar-Nejad, P., and Shneider, N.A. (2022). Antisense oligonucleotide silencing of FUS expression as a therapeutic approach in amyotrophic lateral sclerosis. Nat Med *28*, 104-116.

Lanson, N.A., Jr., Maltare, A., King, H., Smith, R., Kim, J.H., Taylor, J.P., Lloyd, T.E., and Pandey, U.B. (2011). A Drosophila model of FUS-related neurodegeneration reveals genetic interaction between FUS and TDP-43. Hum Mol Genet *20*, 2510-2523.

Machamer, J.B., Collins, S.E., and Lloyd, T.E. (2014). The ALS gene FUS regulates synaptic transmission at the Drosophila neuromuscular junction. Hum Mol Genet *23*, 3810-3822.

Matsumoto, T., Matsukawa, K., Watanabe, N., Kishino, Y., Kunugi, H., Ihara, R., Wakabayashi, T., Hashimoto, T., and Iwatsubo, T. (2018). Self-assembly of FUS through its low-complexity domain contributes to neurodegeneration. Hum Mol Genet *27*, 1353-1365.

Miguel, L., Avequin, T., Delarue, M., Feuillette, S., Frebourg, T., Campion, D., and Lecourtois, M. (2012). Accumulation of insoluble forms of FUS protein correlates with toxicity in Drosophila. Neurobiol Aging *33*, 1008 e1001-1015.

Moens, T.G., Da Cruz, S., Neumann, M., Shelkovnikova, T.A., Shneider, N.A., and Van Den Bosch, L. (2025). Amyotrophic lateral sclerosis caused by FUS mutations: advances with broad implications. Lancet Neurol *24*, 166-178.

Monahan, Z., Ryan, V.H., Janke, A.M., Burke, K.A., Rhoads, S.N., Zerze, G.H., O'Meally, R., Dignon, G.L., Conicella, A.E., Zheng, W.*, et al.* (2017). Phosphorylation of the FUS low-complexity domain disrupts phase separation, aggregation, and toxicity. EMBO J *36*, 2951-2967.

Murakami, T., Qamar, S., Lin, J.Q., Schierle, G.S., Rees, E., Miyashita, A., Costa, A.R., Dodd, R.B., Chan, F.T., Michel, C.H.*, et al.* (2015). ALS/FTD Mutation-Induced Phase Transition of FUS Liquid Droplets and Reversible Hydrogels into Irreversible Hydrogels Impairs RNP Granule Function. Neuron *88*, 678-690.

Picchiarelli, G., Demestre, M., Zuko, A., Been, M., Higelin, J., Dieterle, S., Goy, M.A., Mallik, M., Sellier, C., Scekic-Zahirovic, J.*, et al.* (2019). FUS-mediated regulation of acetylcholine receptor transcription at neuromuscular junctions is compromised in amyotrophic lateral sclerosis. Nat Neurosci.

Rhine, K., Makurath, M.A., Liu, J., Skanchy, S., Lopez, C., Catalan, K.F., Ma, Y., Fare, C.M., Shorter, J., Ha, T.*, et al.* (2020). ALS/FTLD-Linked Mutations in FUS Glycine Residues Cause Accelerated Gelation and Reduced Interactions with Wild-Type FUS. Mol Cell *80*, 666-681 e668.

Scekic-Zahirovic, J., El Oussini, H., Mersmann, S., Drenner, K., Wagner, M., Sun, Y., Allmeroth, K., Dieterle, S., Sinniger, J., Dirrig-Grosch, S.*, et al.* (2017). Motor neuron intrinsic and extrinsic mechanisms contribute to the pathogenesis of FUS-associated amyotrophic lateral sclerosis. Acta Neuropathol (Berl) *133*, 887-906.

Steyaert, J., Scheveneels, W., Vanneste, J., Van Damme, P., Robberecht, W., Callaerts, P., Bogaert, E., and Van Den Bosch, L. (2018). FUS-induced neurotoxicity in Drosophila is prevented by downregulating nucleocytoplasmic transport proteins. Hum Mol Genet *27*, 4103-4116.

Xia, R., Liu, Y., Yang, L., Gal, J., Zhu, H., and Jia, J. (2012). Motor neuron apoptosis and neuromuscular junction perturbation are prominent features in a Drosophila model of Fus-mediated ALS. Mol Neurodegener *7*, 10.

## 1st Editorial Decision 16th Jan 2026

Dear Luc,

Thank you again for transferring your revised manuscript EMBOJ-2025-122972-T for consideration by The EMBO Journal, along with the reports of the referees who had previously reviewed the original version at another journal and your point-by-point response to them, and for your patience during peer review. As I have already informed you, your revised manuscript has now been seen by the original referees #1 and #3 (renumbered as referees #1 and #2 in the new referee panel, respectively), as well as a new referee (#3). I have already shared with you a copy of their comments, which are appended again below.

Referee #1 acknowledges that most of their previous concerns have been addressed in the revised manuscript, but has a number of technical and other comments on the new data, which should be addressed. Referee #2 points out that the results described in the new version of the manuscript are novel, relevant, and compelling, but still has a number of suggestions for additional experimental data, including the need for proteome-level validation (major point 1), and the need for further investigation of the transcriptional targets that are involved in muscle development. While we agree that such further insights would be desirable and increase the impact of the study on the field, they would not be strictly required for further consideration of the manuscript here. Finally, referee #3 also recognizes that the topic of the manuscript is relevant and the study well-performed, but emphasizes that the manuscript suffers from over-interpretation, which must be addressed, and provides detailed suggestions for further improvement.

On balance, in light of the referees' comments and recommendations, I would like to invite you to submit a thoroughly revised version of your manuscript taking the referees' suggestions on board, along with a detailed point-by-point response addressing all referees' comments. Please note that it is The EMBO Journal policy to allow only a single round of major revision, and acceptance of your manuscript will therefore depend on the completeness of your responses in this revised version. Given the nature of the raised concerns, I think that it would be useful to discuss your revision plan already during the initial phase of your revision. You are very welcome to share with me a draft point-by-point response letter/revision plan explaining if there are any points you do not agree with or cannot address, and we could then arrange a video call to discuss further, if necessary.

We generally allow three months as standard revision time (15 April, 2026). As a matter of policy, competing manuscripts published during this period will not negatively impact our assessment of the conceptual advance presented by your study. However, we request that you contact us as soon as possible upon publication of any related work, to discuss how to proceed. Should you foresee a problem in meeting this three-month deadline, please let us know in advance and we will be able to grant an extension.

Please let me know if you have any questions or comments that you would like to discuss with me. Thank you for the opportunity to consider your work for publication in The EMBO Journal. I look forward to your revision.

Best regards,

Ioannis

Ioannis Papaioannou, PhD
Editor, The EMBO Journal
i.papaioannou@embojournal.org

*****
Instructions for preparing your revised manuscript
*****

1. When you are ready to submit the revision, please upload:

- A Word file of the manuscript text (including legends of main Figures, EV Figures and Tables). Please make sure that changes are highlighted (or "tracked") to be clearly visible.

- Individual production-quality figure files (one file per figure). When assembling your figures, please refer to our figure preparation guidelines in order to ensure proper formatting and readability in print as well as on screen:
https://bit.ly/EMBOPressFigurePreparationGuideline
If the data shown in a figure are obtained from n {less than or equal to} 2, please use scatter plots showing the individual data points.
Please also include scale bars in all microscopy images.
The following points must be specified in each figure legend:

i. the name of the statistical test used to generate error bars and P values
ii. the number (n) of independent experiments (please specify technical or biological replicates) underlying each data point (discussion of statistical methodology can be reported in the Materials and Methods section, but figure legends should contain a basic description of n, P, and the test applied)
iii. the nature of the bars and error bars (s.d., s.e.m.).
See also guidelines for figure legends: https://www.embopress.org/page/journal/14602075/authorguide#figureformat

- A point-by-point response to the referees' comments, with a detailed description of the changes made (as a word file). All referees' concerns must be fully addressed and their suggestions taken on board. When preparing your letter of response to the referees' comments, please bear in mind that this will form part of the Review Process File and will therefore be available online to the community. Please note that you have the possibility to opt out of the transparent process at any stage prior to publication by letting the editorial office know (contact@embojournal.org); if you do opt out, the Review Process File link will point to the following statement: "No Review Process File is available with this article, as the authors have chosen not to make the review process public in this case.". For more details on our Transparent Editorial Process, please visit our website: https://www.embopress.org/page/journal/14602075/authorguide#transparentprocess

- Expanded View (EV) files (replacing Supplementary Information) that are collapsible/expandable online. A maximum of 5 EV Figures can be typeset. EV Figures should be cited as "Figure EV1, Figure EV2" etc. in the text, and their respective legends should be included in the manuscript file after the legends of regular figures. See detailed instructions regarding Expanded View files here:
https://www.embopress.org/page/journal/14602075/authorguide#expandedview

- For the figures that you do NOT wish to display as Expanded View figures, they should be bundled together with their legends in a single PDF file called "Appendix", which should start with a short Table of Contents (including page numbers). Appendix figures should be referred to in the main text as: "Appendix Figure S1, Appendix Figure S2" etc. Please see detailed instructions here: https://www.embopress.org/page/journal/14602075/authorguide#expandedview

- A complete author checklist, which you can download from our author guidelines (https://www.embopress.org/page/journal/14602075/authorguide). Please note that the checklist will also be part of the Review Process File.

2. Please note that no statistics should be calculated and shown in Figures if n=2. Please also note that each p value should be reported as an exact value.

3. Before submitting your revision, primary datasets (and computer code, where appropriate) produced in this study need to be deposited in appropriate public databases (see https://www.embopress.org/page/journal/14602075/authorguide#dataavailability). In particular, all datasets such as DNA or RNA sequencing data, or mass spectrometry data generated in the study must be deposited in appropriate repositories. The accession numbers, database, and the specific URLs (links) should be listed in a formal "Data availability" section (placed after Methods), following the example below:

"The RNA-seq datasets produced in this study are available in the following database:
Gene Expression Omnibus GSE46843 (https://www.ncbi.nlm.nih.gov/geo/query/acc.cgi?acc=GSE46843)"

*** All links should resolve to a page where the data can be accessed. ***

*** Please remember to provide in the Data availability section of your revised manuscript reviewer passwords if the datasets are not yet public. ***

*** The Data Availability Section is restricted to new primary data that are part of this study. In case you have no data that require deposition in a public database, please state so instead of referring to the database: "Our study includes no data deposited in public repositories." under the heading "Data availability". ***

4. The materials and methods need to be described in the manuscript using our structured methods format, which is now required for all research articles. According to this format, the Methods section includes a single "Reagents and Tools Table" - listing key reagents, experimental models, software and relevant equipment including their sources and relevant identifiers- followed by a "Methods and Protocols" section describing the methods. Please download and fill our Reagents and Tools Table template (.docx), which you can find in our author guide:
https://www.embopress.org/page/journal/14602075/authorguide#structuredmethods. When submitting your revised manuscript, please do not include the Reagents and Tools Table in the Methods section of the manuscript but instead upload it as a separate file choosing the file type "Reagent Table".

5. Please check that the title and the abstract of the manuscript are brief, yet explicit, even to non-specialists. The length of the title should not exceed 100 characters, and the abstract should be a single paragraph not exceeding 175 words.

6. Please also note our reference format: https://www.embopress.org/page/journal/14602075/authorguide#referencesformat.

7. At EMBO Press we ask authors to provide source data for the main manuscript figures. You will receive a separate email with instructions for providing source data with your revised manuscript, including how to upload and organize the files.

8. Please remember: digital image enhancement is acceptable practice, as long as it accurately represents the original data and conforms to community standards. If a figure has been subjected to significant electronic manipulation, this must be noted in the figure legend or in the "Materials and Methods" section. The editors reserve the right to request original versions of figures and the original images that were used to assemble the figure.

9. Our journal encourages inclusion of data citations in the reference list to directly cite datasets that were obtained from public databases. Data citations in the article text are distinct from normal bibliographical citations and should directly link to the database records from which the data can be accessed. In the main text, data citations are formatted as follows: "Data ref: Smith et al, 2001" or "Data ref: NCBI Sequence Read Archive PRJNA342805, 2017". In the Reference list, data citations must be labeled with "[DATASET]". A data reference must provide the database name, accession number/identifiers, and a resolvable link to the landing page from which the data can be accessed at the end of the reference. Further instructions are available at: https://www.embopress.org/page/journal/14602075/authorguide#referencesformat.

10. We request authors to consider both actual and perceived competing interests. Please review our policy (https://www.embopress.org/page/journal/14602075/authorguide#conflictsofinterest) and update your competing interests statement if necessary. Please name this section 'Disclosure and competing interests statement' and place it after the Acknowledgements section.

11. Please note that all corresponding authors are required to provide an ORCID ID upon submission of a revised manuscript (https://orcid.org/). Please find instructions on how to link your ORCID ID to your account in our manuscript tracking system in our Author guidelines (https://www.embopress.org/page/journal/14602075/authorguide#authorshipguidelines).

12. We use CRediT to specify the contributions of each author in the journal submission system. CRediT replaces the author contribution section, which should be removed from the manuscript. Please use the free text box to provide more detailed descriptions. See also guide to authors: https://www.embopress.org/page/journal/14602075/authorguide#authorshipguidelines.

13. Further information is available in our Guide For Authors: https://www.embopress.org/page/journal/14602075/authorguide

14. We would also welcome the submission of cover suggestions or motifs to be used by our Graphics Illustrator in designing a cover.

15. Please use the link below to submit your revision:
https://emboj.msubmit.net/cgi-bin/main.plex

------------------------------------------------

Referee #1:

The authors have addressed most of my comments.
I have questions on the new cellular data added - Fig.8f,g (images) and h-k (quantification). In order to justify LLPS mention in the manuscript title, these experiments require different presentation, inclusion of QC data and clarifications.
1. These STED data in Hek cells as require general plane images showing (nuclear?) localisation of these condensates.
2. Scale bar is 400 nm. Meaning that they are quite large condensates. How was OE controlled for these experiments and was the condensate size distribution? What was the population of cells utilised for quantification purposes - with regards to protein expression and condensate size? Or rather, all cells were included in the quantification, regardless condensate size?
3. Most critically, the images suggest that the condensation propensity of WT FUS and its mutants is similar (similar numbers of condensates in the fields of view shown). Is this the case and why if so - when mutations are expected to affect condensation? Has the condensation propensity of mutants been quantified (condensate area/number).
4. Reduced ETV5 partitioning into FUS condensates (or their reduced co-condensation) could be caused by altered properties of these condensates - e.g. they might not be LLPS anymore and become more solid. Given that LLPS-dependent ETV5 co-condensation with FUS is one of central claims in the manuscript, the LLPS nature of these assemblies in cells should be addressed - e.g. by FRAP at least.
5. WB data showing similar expression of these FUS mutants is required.

Referee #2:

As a DNA/RNA-binding protein, FUS has been extensively studied in ALS pathogenesis primarily through the lens of gain-of-toxic-function mechanisms. However, whether disease-associated mutations cause loss-of-function effects remains insufficiently investigated. In this manuscript, PICCHIARELLI et al. address this critical gap by employing a comprehensive multi-model approach, including FUS-ALS patient muscle biopsies, iPSC-derived myotubes, FUS knockout and mutant mouse models, and the Drosophila model, to demonstrate that both FUS mutation and deletion lead to convergent phenotypes of disrupted sarcomeres and aberrant mitochondrial morphology. The authors further perform RNA-seq on embryonic cells from FUS KO and mutant mice, as well as siFUS-treated C2C12 cells, revealing coordinated downregulation of multiple muscle mitochondria-associated transcripts. Through rigorous transcription factor analysis, they identify MEF2 and the PEA3-family factor ETV as key downstream effectors, thereby elucidating a transcriptional regulatory axis through which FUS modulates muscle mitochondrial protein expression. Mechanistically, the study demonstrates that FUS interacts with MEF2 and ETV5 via liquid-liquid phase separation (LLPS), and that ALS-linked mutations disrupt this functional complex, resulting in loss of transcriptional regulation.

Overall, this work establishes a previously underappreciated role for FUS in muscle development and structural maintenance, and provides compelling mechanistic insights into how FUS mutations precipitate muscle pathology in ALS. The integration of human samples, multiple in vivo and in vitro models, and orthogonal experimental strategies substantially strengthens the conclusions and represents an advance in understanding the multifaceted nature of FUS-associated neurodegeneration.

However, there are a couple of major concerns that need to be addressed to support the conclusions.

major concerns：

1) The authors investigate muscle pathology, examining ultrastructure of muscle and mitochondria of FUS-ALS patient samples and iPSC-derived myotubes (Fig. 1), FUS knockout and mutant mouse models (Fig. 2), and Drosophila model (Figs. 3 & 6). RNA sequencing of FUS-deficient and mutant mouse embryonic cells and siFUS-treated C2C12 cells reveals downregulation of muscle mitochondria-related transcripts (Figs. 4, 5, and 7), which the authors mechanistically link to MEF2 and ETV transcription factors through luciferase assays and EMSA (Figs. 8 and 9). They performed co-IP (Fig. 8) and LLPS analysis with recombinant proteins (Fig. 9), and STED microscopy in HEK293 cells by overexpressing FUS and ETV or MEF2. However, the study lacks protein-level validation of FUS, MEF2A, and ETV5, and crucially, their mitochondrial protein targets, in the patient, mouse, and fly models. This represents a critical gap, as RNA changes do not necessarily predict protein alterations. To address this, proteomic profiling of FUS knockout/mutant mouse embryonic cells and siFUS-treated C2C12 cells would be invaluable to definitively identify which muscle development-associated mitochondrial proteins are directly impacted by FUS dysfunction. Such orthogonal validation would substantially strengthen the mechanistic conclusions.
2) The study identified FUS-mediated regulation of transcription factors MEF2A and ETV5, yet provides insufficient detail regarding which specific mitochondrial proteins are controlled during muscle development. The data presented in Figures 4d and f are limited to RNA-level changes and lack the necessary granularity: which mitochondrial proteins are direct transcriptional targets? How do these proteins mechanistically regulate muscle development? Are their expression and function altered in ALS patients or disease models? These questions require more precise investigation.
3) In RNA-seq analysis, the authors used gastrocnemius muscles of E18.5 FUS-/- and FUS NLS/ NLS mice and C2C12 cells with siRNA-mediated KD of FUS. It would be more reasonable to try the iPSC line from at least a FUS-ALS patient used in Figure 1 and C2C12 cells with FUS KO by CRISPR/Cas9.

Minor:
1) The Discussion would be substantially strengthened by the inclusion of a schematic model diagram illustrating: (1) the relationships and molecular mechanisms among FUS, MEF2A, ETV5, and their target genes Mrps18C and/or Mrpl30; (2) the regulatory connections between these transcription factors and broader muscle mitochondrial protein networks. Such a figure should employ solid lines to denote established interactions/mechanisms and dashed lines to indicate speculative or unclear relationships, thereby providing readers with a clear visualization of the proposed pathway and highlighting current gaps in understanding.
2) Page 19, lines 464-465: This appears to contain an error. The text should presumably state that addition of ETV5 promoted the affinity between FUS and MEF2A, consistent with the earlier description that "MEF2A/FUS interaction was increased by addition of ETV5" (Fig. 8a-b). Please verify and correct as necessary.

Referee #3:

The study by Picchiarelli and Wienand et al. aims to gain mechanistic insight into cell-autonomous muscle dysfunction due to NLS-deficient mutant FUS, causative of an early onset genetic form of ALS. This is a critically important question and this

manuscript is very nicely written. The authors employ a remarkable breadth of models and approaches, and this work is truly a tour de force. The authors present evidence that FUS potentiates MEF2 in muscle in a ETV5-dependent manner, and mutations that truncate the NLS disrupt this coordination.

In summary, this is excellent work but the manuscript as it stands suffers from over-interpretation and may be more digestible if reframed into a muscle differentiation story that takes place prior to the specialization of subsynaptic nuclei that restricts ETV5 expression and saves any mention of ALS for the Discussion section. From a disease perspective, the notion that mutant FUS toxicity in muscle is a loss of function phenomenon (as compared to a gain of function in the CNS) is quite intriguing, but changing a paradigm requires multiple lines of strong supporting evidence; while this study indeed has multiple lines of evidence, they are not sufficiently strong to support the conclusions as they stand. Hence, while this study is generally elegant, there are major concerns with its framing and over-interpretation of data.

Key issues that need to be addressed are as follows:

1) The tie to ALS is extremely weak and somewhat distracting. As another reviewer from the previous round pointed out, the cause of muscle ultrastructure disruption in ALS is impossible to delineate from TEM of patient muscle (indeed, it was also observed in sporadic ALS), thus Figure 1 does not add to the story. If the authors examined muscle from patients with FUS ALS prior to symptom onset and motor neuron degeneration (likely unfeasible), and/or myotubes differentiated from iPSCs of multiple FUS-ALS donors, and/or FusΔNLS/+ mice crossed with a CNS Cre for CNS rescue, the premise that there is cell autonomous muscle dysfunction in FUS ALS would be much stronger. Given that FusΔNLS/+ mice have a profound reduction in hang time at 12 months, TEM of 22 month FusΔNLS/+ muscle seems very late stage and somewhat misleading. The TEM in Fig 2 simply provides evidence that nuclear FUS is necessary for muscle development/differentiation; FusΔNLS/+ mice crossed with a line expressing Cre in post-mitotic muscle would have supported the authors' premise much stronger. The Drosophila model in Fig 3 is more informative as it ablated Fus/caz in post-fusion muscle, but the decrease in muscle number suggests loss of caz causes myocyte death. Hence, the authors need to relegate Fig 1 to supplementary, and if none of the above experiments can be performed, then the results must not be interpreted as evidence of ALS relevant muscle pathology.

2) The mechanistic studies described in the rest of the manuscript are excellent and provide critical insight into Fus potentiation of MEF2 transcriptional activity. While this work is expertly done, this interpretation should be restructured, as extrapolation to muscle maintenance is poorly supported. All three models used for RNA-seq are differentiation/development models, and defects in differentiation would explain the observed transcriptional changes. As a primary role for Fus in muscle differentiation was clearly supported by the embryonic mouse models, it raises the question of why pathway analysis of Fus ChIP-Seq in C2C12 did not point toward any muscle-specific pathway.

3) It is also unclear from the methods if ChIP-seq was performed on differentiating C2C12 cells or proliferating; the section on C2C12 culture states they were differentiated but the section on chromatin immunoprecipitation does not. If the authors indeed performed ChIP-seq on C2C12 cells in their proliferating myoblast stage, this was an unfortunately major oversight and the experiment should be performed in differentiated cells (or even better, in mature muscle from adult mice) to determine if Fus truly regulates muscle genes in mammals.

4) Why was Mrps18c chosen as a Fus-responsive gene instead of a muscle-specific gene (such as one of the genes highlighted in Fig 4f)? Increase in Mef2 activity is ETV5-dependent, yet the authors state ETV5 is only expressed in subsynaptic nuclei (as is ETV4, and ETV1 does not seem to be expressed in muscle at appreciable levels - https://research.cchmc.org/myoatlas/), thus it is unclear how mutations in Fus could lead to muscle ultrastructural changes outside of development.

5) A more thorough characterization of the Fus/Etv5 mouse model presented in Fig 6c is warranted: what was their body weight, muscle weight, muscle fiber cross sectional area, fiber type composition (since mitochondria was repeatedly implicated and discussed), NMJ morphology, spinal cord motor neuron size and number, etc.; there seems to be a lot of informative data that can be gleamed from this model other than simply their hang time.

**Referee #1:**

The authors have address most of my comments.
I have questions on the new cellular data added - Fig.8f,g (images) and h-k (quantification). In order to justify LLPS mention in the manuscript title, these experiments require different presentation, inclusion of QC data and clarifications.

1. These STED data in Hek cells as require general plane images showing (nuclear?) localisation of these condensates.

We now provide widefield images showing these condensates are nuclear (**Appendix Figure 9**). All quantifications were performed on condensates localized in nuclei.

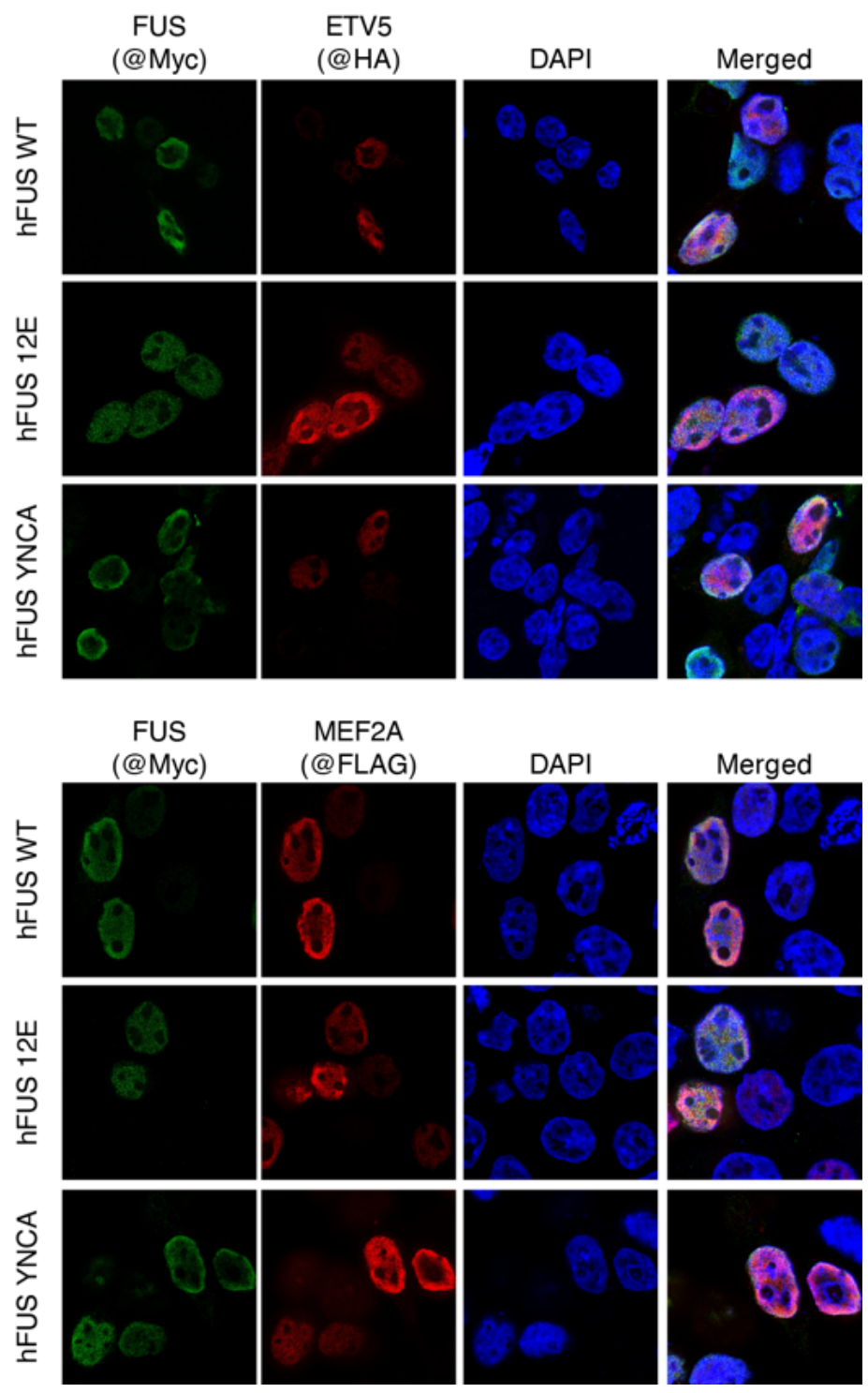


**Appendix Figure 9: subcellular localization of FUS, ETV5 and MEF2A proteins in transfected HEK293 cells**
HEK293 cells were transfected with indicated Myc-tagged FUS constructs and either HA-ETV5 (upper panel) or FLAG-MEF2A (lower panel). Cells were stained with anti-myc (green), anti-HA (upper panel, red) or anti-FLAG (lower panel, red). Shown are representative immunofluorescence images of the indicated proteins. Note that all constructs lead to primarily nuclear localization.

This is now reported as such in the results section (lines 345-347): « *As expected, FUS condensates were nuclear (..) (**Appendix Figure 9-10**).”*

2. Scale bar is 400 nm. Meaning that they are quite large condensates. How was OE controlled for these experiments and was the condensate size distribution? What was the population of cells utilised for quantification purposes - with regards to protein expression and condensate size? Or rather, all cells were included in the quantification, regardless condensate size?

We performed the quantification on double positive cells (all cells being transfected with FUS expression vectors and either ETV5 or MEF2A expression vector). We did not select cells based on overexpression levels or condensate size to avoid observer-induced bias.
The condensate size distribution for all three proteins is indicated below in the different experimental conditions.

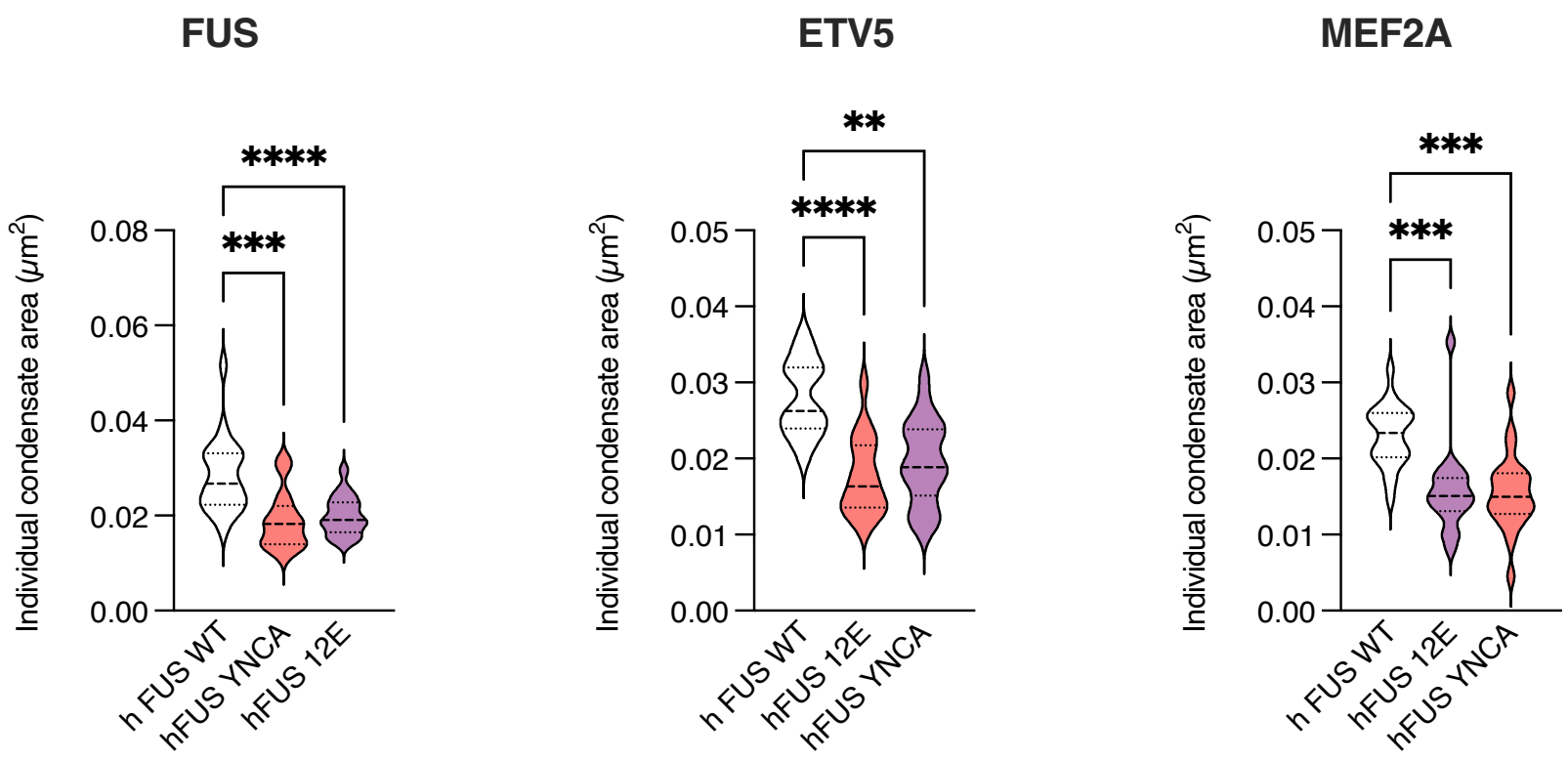


Condensates of FUS, ETV5 and MEF2A appear to have a median area between 0.02 and 0.03$\mu m^2$, and thus median diameter in the range of 200nm. This is consistent with what observed for most nuclear bodies, including FUS condensates (Banani *et al*, 2017; Patel *et al*, 2015). Interestingly, condensate sizes of FUS, ETV5 and MEF2A are significantly decreased by expression of LLPS-deficient FUS.
These data are now included in ***Appendix Figure 10***, and reported as follows in the results section:
lines 345-347 « *As expected, FUS condensates were nuclear and smaller in size and decreased in density when FUS carried LLPS deficient mutations (**Appendix Figure 9-10**).”*
lines 358-360: *“ETV5 and MEF2A condensates were smaller when expressed with LLPS-deficient FUS as compared to expression with wild type FUS (**Appendix Figure 10**).”*

3. Most critically, the images suggest that the condensation propensity of WT FUS and its mutants is similar (similar numbers of condensates in the fields of view shown). Is this the case and why if so - when mutations are expected to affect condensation? Has the condensation propensity of mutants been quantified (condensate area/number).

We thank the reviewer for this comment. To answer rigorously, we measured the individual condensate area and the surface covered by these condensates per surface area. Results are shown below, and in novel ***Appendix Figure 10***.

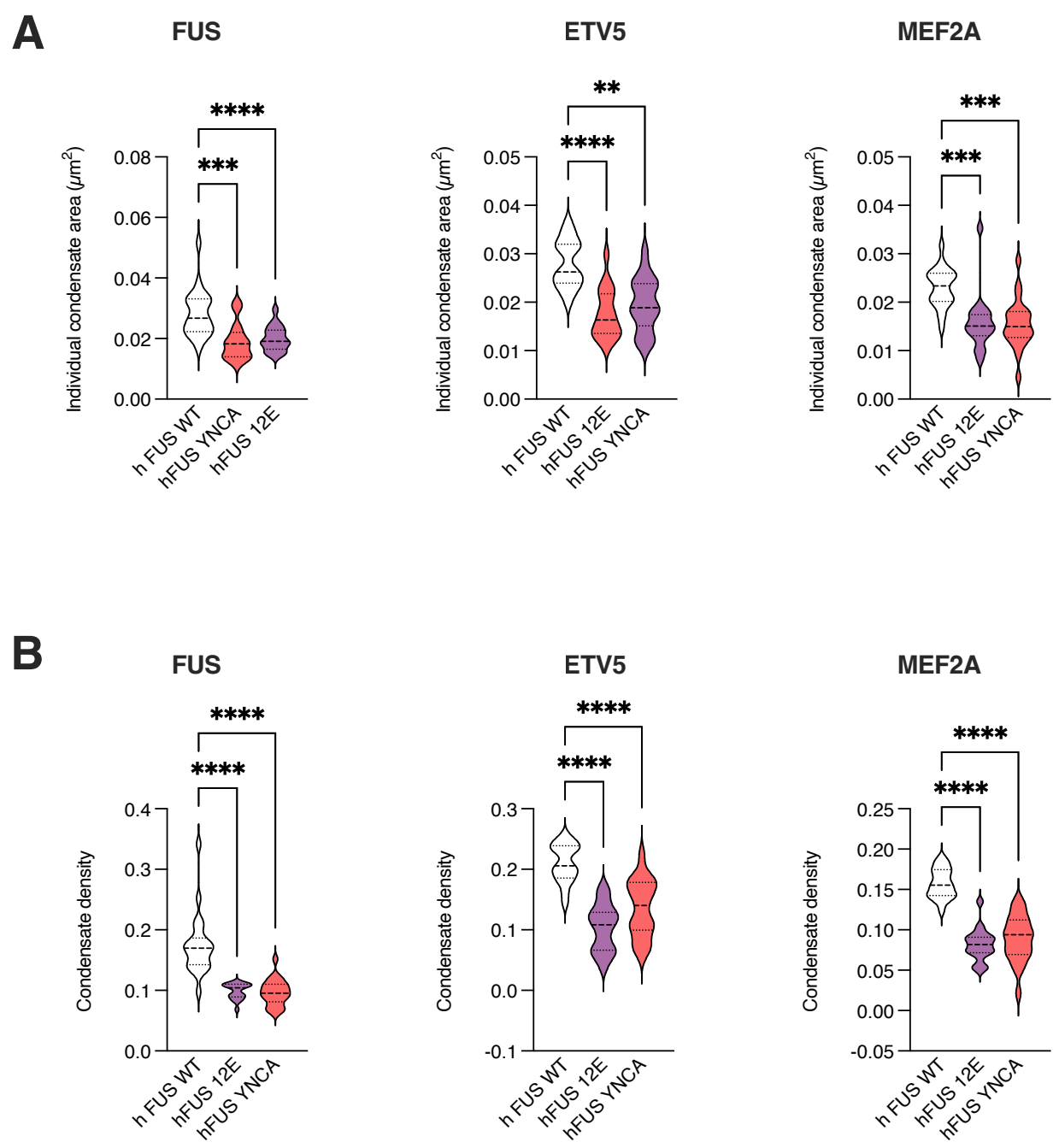


**Appendix Figure 10: characterization of FUS, ETV5 and MEF2A condensates using STED imaging.**
A-B: quantification of individual condensate size ($\mu m^2$, A) or density (fraction of occupied area, B) for the indicated condensates shown in Figure 8F-K.
***, p<0.001, ****, p<0.0001 One way ANOVA followed by Tukey.

As expected, the expression of LLPS deficient FUS is sufficient to decrease density of FUS condensates, as well as the surface area covered and individual condensate size for this protein. Consistent with our hypothesis, these parameters were also decreased for ETV5 and MEF2A upon expression of LLPS deficient FUS rather than wild type FUS. Thus, condensation propensity of LLPS deficient FUS is decreased, and this impacts condensates of co-expressed ETV5 or MEF2A. These results have been included in ***Appendix Figure 10*** and cited in the text as such:
lines 345-347 *« As expected, FUS condensates were nuclear and smaller in size and decreased in density when FUS carried LLPS deficient mutations (**Appendix Figure 9-10**)."*
lines 358-360: *"ETV5 and MEF2A condensates were smaller when expressed with LLPS-deficient FUS as compared to expression with wild type FUS (**Appendix Figure 10**)."*

4. Reduced ETV5 partitioning into FUS condensates (or their reduced co-condensation) could be caused by altered properties of these condensates - e.g. they might not be LLPS anymore and become more solid. Given that LLPS-dependent ETV5 co-condensation with FUS is one of central claims in the manuscript, the LLPS nature of these assemblies in cells should be addressed - e.g. by FRAP at least.

We agree with the reviewer that it is a of key importance to determine how ETV5 and/or MEF2A might affect liquid properties of FUS condensates.
We addressed this point by performing fluorescence recovery after photobleaching (FRAP) following "half-bleach" of FUS-EGFP condensates. Our data clearly show that in presence of ETV5 and/or MEF2A, FUS shows increased internal mobility within the condensates indicating the presence of those factors does not result in enhanced solidification of FUS (see below).

These results have been included in **Fig EV4** and the results section, and also the methods section has been modified accordingly. The results section now includes the following sentence (lines 325-330) : *« To address properties of FUS condensates in absence or presence of ETV5 and/or MEF2A, we subsequently performed fluorescence recovery after photobleaching (FRAP) following "half-bleach" of FUS-EGFP condensates. In the presence of ETV5 and/or MEF2A, FUS shows increased internal mobility within the condensates indicating that the presence of those factors does not result in enhanced solidification of FUS (**Fig EV4**)."*

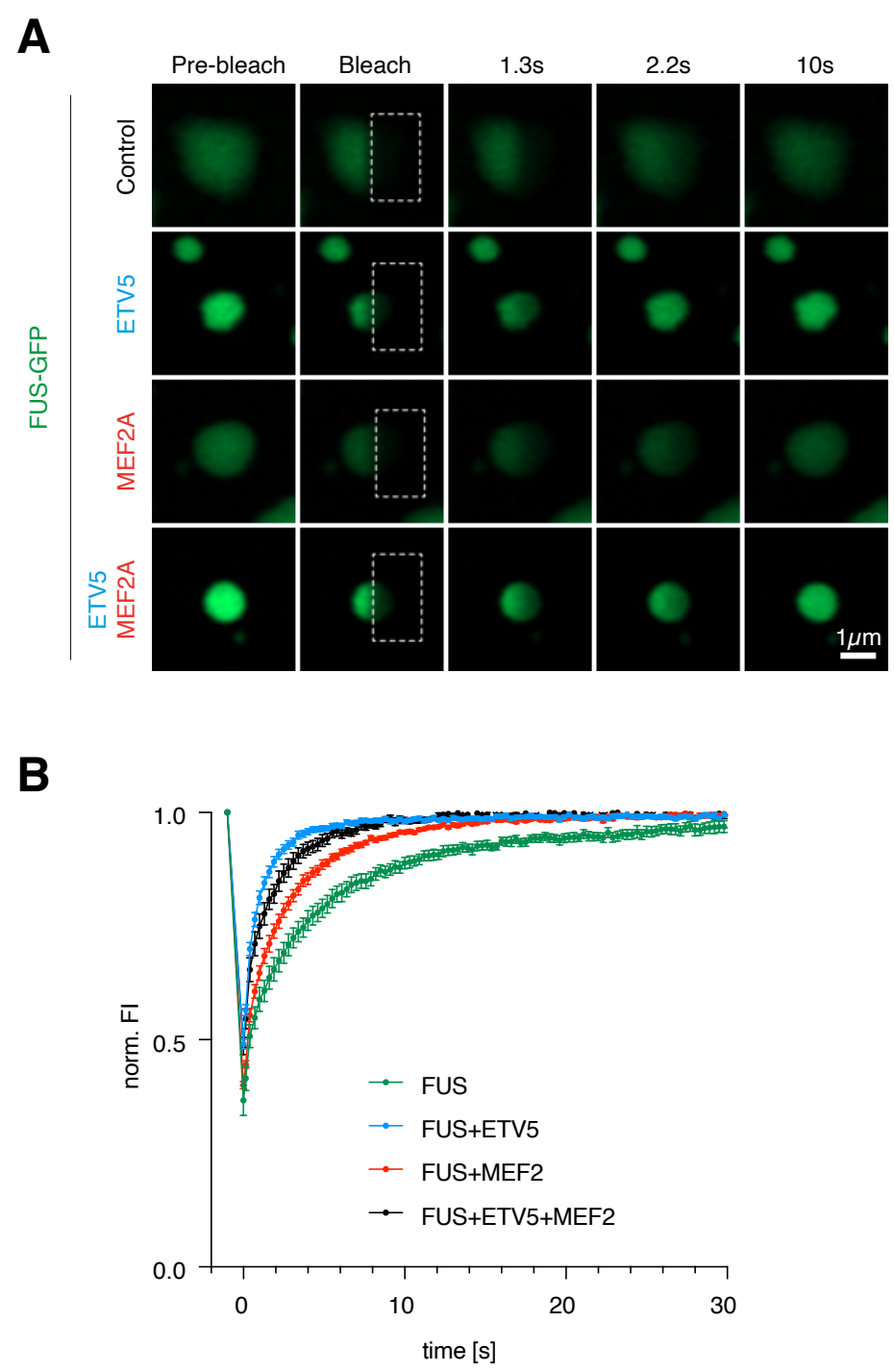

**Figure EV4: effects of MEF2A and ETV5 on FUS liquidity**
A : Representative FRAP images of GFP-FUS condensates (15 $\mu$M) at the indicated time points, in the absence or presence of ETV5 and/or MEF2A. Dotted box indicates the bleached area (half of the condensate). Scale bar: 1 $\mu$m.
B : FRAP recovery curves following half-bleaching of GFP-FUS condensates in absence or presence of ETV5 and/or MEF2A. Shown is the mean ± SEM of 19-25 condensates out of a total of three independent experiments.

5. WB data showing similar expression of these FUS mutants is required.

These data were already present in the manuscript in Figure EV5. We have now added the quantifications of 3 experiments. In our hands, the LLPS deficient FUS mutants showed the expected increase in electrophorectic mobility (Monahan *et al*, 2017). We also observed higher levels of protein in western blotting, which could be due to their higher solubility (Monahan *et al.*, 2017; Qamar *et al*, 2018) but also their lack of autoregulatory properties (Reber *et al*, 2021).

In any case, these increased FUS levels rule out that the lack of transcriptional co-activation by these LLPS-deficient mutants is due to protein instability or lack of nuclear import. This is now reported in the revised manuscript in ***Appendix Figure 3*** as such : « *Importantly, this lack of activation of MEF2-driven transcription was not due to lack of protein expression, as LLPS-deficient mutants increased FUS protein levels in transfected cells (**Appendix Figure 3**), or to lack of nuclear import as these mutants properly localized to the cell nucleus (**Appendix Figure 3**).”*

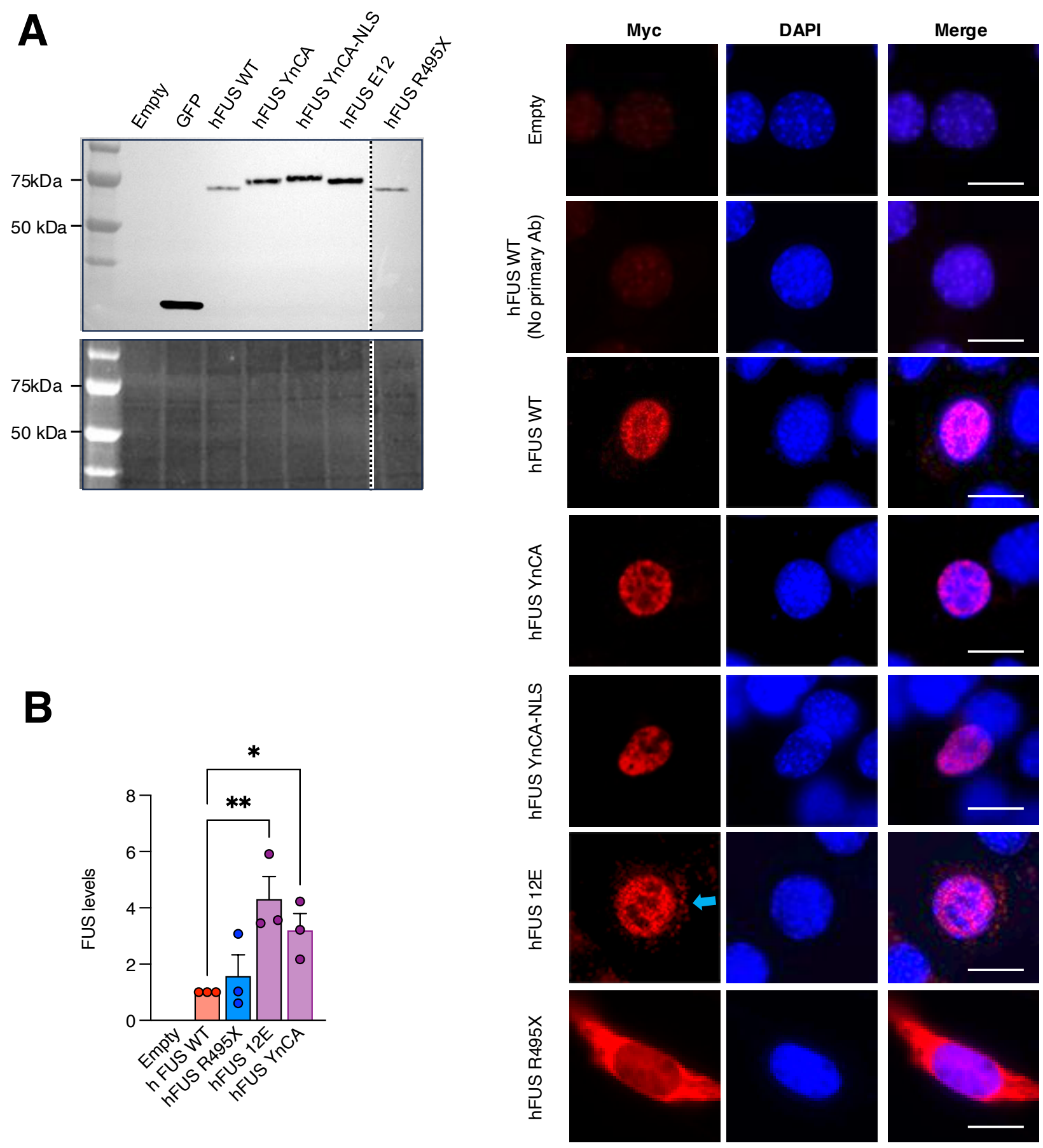


**Appendix Figure 3: Expression levels and subcellular localization of FUS proteins used in this study**
A-B: Representative western blotting of indicated constructs used in Figures 3, 4, 6 and 8 of the manuscript. All encoded proteins (including GFP) are N-terminally Myc-tagged, and an anti-myc antibody was used to visualize the transfected proteins. LLPS-deficient mutants included mutation of 7

tyrosine residues to alanine with (YnCA-NLS) or without an SV40 NLS (YnCA), or 12 phosphomimetic mutations (12E) that have previously been shown to lead to deficient LLPS. LLPS-deficient FUS proteins show a higher immunoreactivity and an increased apparent molecular weight in 3 independent experiments, as quantitated in panel B. Stainfree gel is shown in the bottom panel to indicate equal loading. Note that the original gel included 2 additional FUS constructs that were not used in the current study. These lanes were removed (indicated with the dashed line) to show only constructs relevant to this study. Full uncropped western blot and stainfree gel is included in expanded view data files provided.
One way anova, followed by Tukey, ** adjusted p=0.0040, *, adjusted p=0.028.
C: Representative immunofluorescence of FUS proteins used in this study. All FUS constructs except R495X, showed a nuclear localization. Note that the hFUS12E protein shows some cytoplasmic granular staining. Scale bar: 10$\mu$m.

**Referee #2:**

As a DNA/RNA-binding protein, FUS has been extensively studied in ALS pathogenesis primarily through the lens of gain-of-toxic-function mechanisms. However, whether disease-associated mutations cause loss-of-function effects remains insufficiently investigated. In this manuscript, PICCHIARELLI et al. address this critical gap by employing a comprehensive multi-model approach, including FUS-ALS patient muscle biopsies, iPSC-derived myotubes, FUS knockout and mutant mouse models, and the Drosophila model, to demonstrate that both FUS mutation and deletion lead to convergent phenotypes of disrupted sarcomeres and aberrant mitochondrial morphology. The authors further perform RNA-seq on embryonic cells from FUS KO and mutant mice, as well as siFUS-treated C2C12 cells, revealing coordinated downregulation of multiple muscle mitochondria-associated transcripts. Through rigorous transcription factor analysis, they identify MEF2 and the PEA3-family factor ETV as key downstream effectors, thereby elucidating a transcriptional regulatory axis through which FUS modulates muscle mitochondrial protein expression. Mechanistically, the study demonstrates that FUS interacts with MEF2 and ETV5 via liquid-liquid phase separation (LLPS), and that ALS-linked mutations disrupt this functional complex, resulting in loss of transcriptional regulation.

Overall, this work establishes a previously underappreciated role for FUS in muscle development and structural maintenance, and provides compelling mechanistic insights into how FUS mutations precipitate muscle pathology in ALS. The integration of human samples, multiple in vivo and in vitro models, and orthogonal experimental strategies substantially strengthens the conclusions and represents an advance in understanding the multifaceted nature of FUS-associated neurodegeneration.

However, there are a couple of major concerns that need to be addressed to support the conclusions.

major concerns :

1) The authors investigate muscle pathology, examining ultrastructure of muscle and mitochondria of FUS-ALS patient samples and iPSC-derived myotubes (Fig. 1), FUS knockout and mutant mouse models (Fig. 2), and Drosophila model (Figs. 3 & 6). RNA sequencing of FUS-deficient and mutant mouse embryonic cells and siFUS-treated C2C12 cells reveals downregulation of muscle mitochondria-related transcripts (Figs. 4, 5, and 7), which the authors mechanistically link to MEF2 and ETV transcription factors through luciferase assays and EMSA (Figs. 8 and 9). They performed co-IP (Fig. 8) and LLPS analysis with recombinant proteins (Fig. 9), and STED microscopy in HEK293 cells by overexpressing FUS and ETV or MEF2. However, the study lacks protein-level validation of FUS, MEF2A, and ETV5, and crucially, their mitochondrial protein targets, in the patient, mouse, and fly models. This represents a critical gap, as RNA changes do not necessarily predict protein alterations. To address this, proteomic profiling of FUS knockout/mutant mouse embryonic cells and siFUS-treated C2C12 cells would be invaluable to definitively identify which muscle development-associated mitochondrial proteins are directly impacted by FUS dysfunction. Such orthogonal validation would substantially strengthen the mechanistic conclusions.

While we generally agree that the study would be strengthened by the suggested experiments, we consider these experiments to go beyond the current study.

We would like to highlight that another collaborative study with Dr Kabashi (Mingaj and collaborators, in consideration elsewhere, and attached to the submission) provides many of these orthogonal validations in zebrafish, mouse and iPSCs. In particular, similar results on muscle structure in zebrafish with Fus mutations are observed, and Dr Kabashi performed metabolomic characterization along with mitochondrial function assays in different models, that all confirm extensively our current study and provide deeper mechanistic insights into metabolic dysfunction and molecular players downstream MEF2A and ETV5. We hope that this second study provides answers to the points raised by this reviewer.

2) The study identified FUS-mediated regulation of transcription factors MEF2A and ETV5, yet provides insufficient detail regarding which specific mitochondrial proteins are controlled during muscle development. The data presented in Figures 4d and f are limited to RNA-level changes and lack the necessary granularity: which mitochondrial proteins are direct transcriptional targets? How do these proteins mechanistically regulate muscle development? Are their expression and function altered in ALS patients or disease models? These questions require more precise investigation.

Regarding the roles of MEF2A and ETV5 in muscle development and neurons, this is discussed in the discussion section, and will require additional studies that go beyond the current manuscript.

Lines 430-440 : « *FUS, MEF2 and PEA3 transcription factors are broadly expressed, and our findings could thus have consequences beyond muscle development. In adult muscle, both Fus and Mef2a are expressed in myonuclei, with an enrichment in subsynaptic nuclei (Huang et al, 2020; Petrany et al, 2020; Picchiarelli et al, 2019), while Etv5 expression is restricted to subsynaptic nuclei (Hippenmeyer et al, 2007). It is thus likely that the FUS/MEF2A/ETV5 pathway is more relevant for regulating gene expression in subsynaptic than extrasynaptic nuclei in adults, consistent with our previous study (Picchiarelli et al., 2019). Beyond skeletal muscle, both MEF2 and PEA3 transcription factors are expressed in the central nervous system and have been involved in synaptic plasticity (Assali et al, 2019) and neuronal specification (Dalla Torre di Sanguinetto et al, 2008). It will be important to determine whether FUS also coactivates MEF2 and PEA3 transcription factors in neurons."*

We also agree that additional studies in ALS patients and models are required, and, as requested also by reviewer 3, we toned down significantly our claims on ALS and focused the manuscript on the major finding of a role of FUS in muscle development. The requirement for further studies in the field of ALS is discussed in the revised manuscript:
Lines 441-446: *"We also observed that mutation of one allele of FUS, either in FUS-ALS patients or in Fus$^{\Delta NLS/+}$ mice, significantly compromised adult muscle ultrastructure. However, our observation of similarly damaged muscle ultrastructure in sporadic ALS patients suggests that these alterations could be either primary or secondary to denervation (Shefner et al, 2023). Future studies should determine the relevance of these developmentally relevant pathways to late-onset muscle weakness and motor neuron degeneration."*

3) In RNA-seq analysis, the authors used gastrocnemius muscles of E18.5 FUS-/- and FUSDNLS/DNLS mice and C2C12 cells with siRNA-mediated KD of FUS. It would be more reasonable to try the iPSC line from at least a FUS-ALS patient used in Figure 1 and C2C12 cells with FUS KO by CRISPR/Cas9.

We understand the point of the reviewer of requirement of orthogonal validation and highlight that such approaches are provided by the collaborative study with Dr Kabashi (Mingaj and collaborators, in consideration elsewhere, and attached to the submission).

Minor:
1) The Discussion would be substantially strengthened by the inclusion of a schematic model diagram illustrating: (1) the relationships and molecular mechanisms among FUS, MEF2A, ETV5, and their target genes Mrps18C and/or Mrpl30; (2) the regulatory connections between these transcription factors and broader muscle mitochondrial protein networks. Such a figure should employ solid lines to denote established interactions/mechanisms and dashed lines to indicate speculative or unclear relationships, thereby providing readers with a clear visualization of the proposed pathway and highlighting current gaps in understanding.

We now provide this schematic model as **Fig EV5.**
Because the main point of this manuscript is not on mitochondrial function, we rather pointed to transcriptional pathways that we elucidate rather than on the downstream consequences.
The new figure is provided below, and is cited in several places in the discussion section.

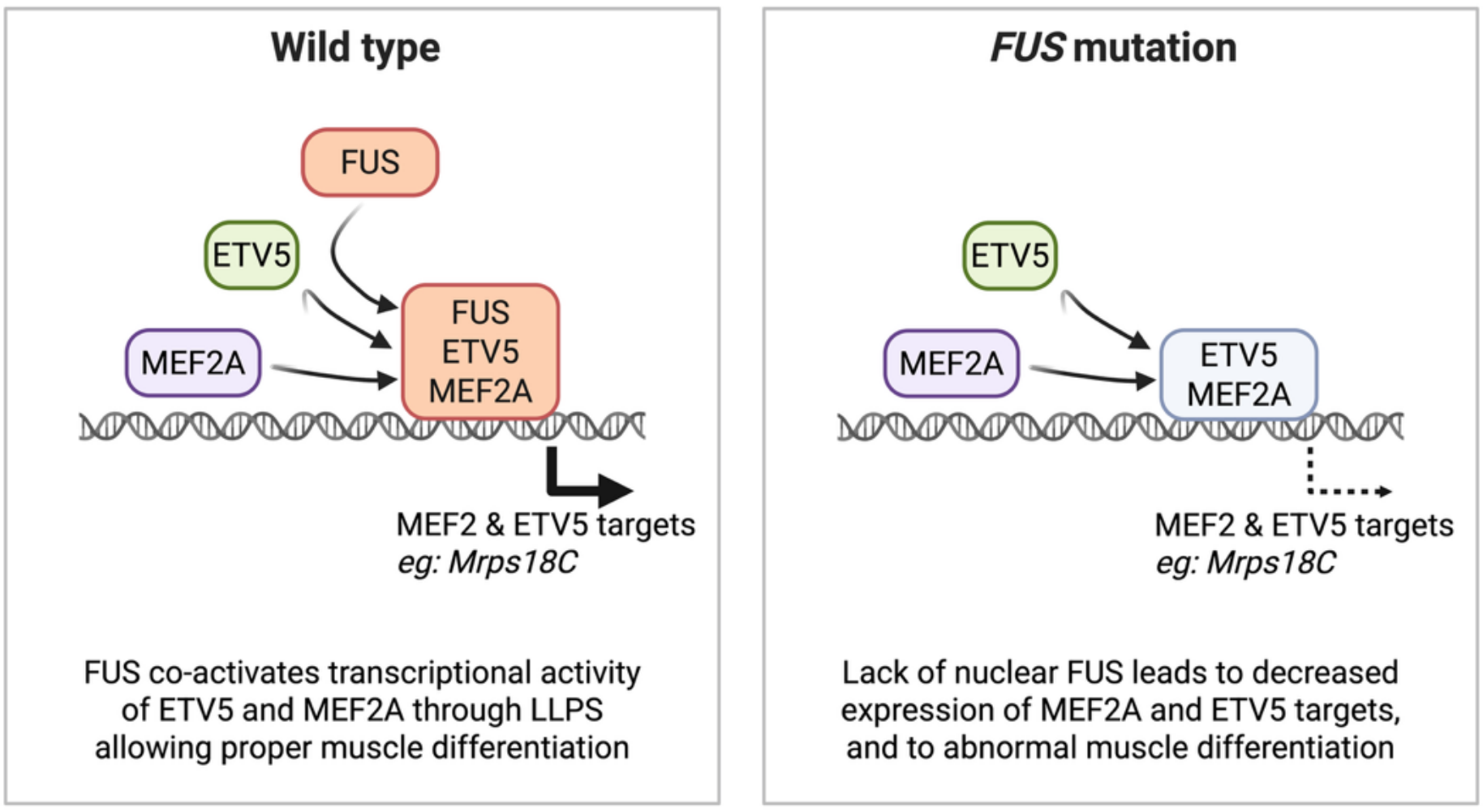


**Figure EV5: working model of FUS, TEV5 and MEF2A interactions on transcription during muscle development.**

2) Page 19, lines 464-465: This appears to contain an error. The text should presumably state that addition of ETV5 promoted the affinity between FUS and MEF2A, consistent with the earlier description that "MEF2A/FUS interaction was increased by addition of ETV5" (Fig. 8a-b). Please verify and correct as necessary.
We reworded the sentence as follows:
Lines 302-306: "*Electrophoretic mobility shift assays (EMSA) using a Mrpl30-derived DNA probe encompassing the FUS binding motif at this gene showed that addition of ETV5 resulted in a diffuse high-molecular-weight smear, consistent with higher order assembly of ETV5 and the probe, and this smear appeared at lower ETV5 concentrations in the presence of FUS and MEF2A*"

**Referee #3:**

The study by Picchiarelli and Wienand et al. aims to gain mechanistic insight into cell-autonomous muscle dysfunction due to NLS-deficient mutant FUS, causative of an early onset genetic form of ALS. This is a critically important question and this manuscript is very nicely written. The authors employ a remarkable breadth of models and approaches, and this work is truly a tour de force. The authors present evidence that FUS potentiates MEF2 in muscle in a ETV5-dependent manner, and mutations that truncate the NLS disrupt this coordination.

In summary, this is excellent work but the manuscript as it stands suffers from over-interpretation and may be more digestible if reframed into a muscle differentiation story that takes place prior to the specialization of subsynaptic nuclei that restricts ETV5 expression and saves any mention of ALS for the Discussion section. From a disease perspective, the notion that mutant FUS toxicity in muscle is a loss of function phenomenon (as compared to a gain of function in the CNS) is quite intriguing, but changing a paradigm requires multiple lines of strong supporting evidence; while this study indeed has multiple lines of evidence, they are not sufficiently strong to support the conclusions as they stand. Hence, while this study is generally elegant, there are major concerns with its framing and over-interpretation of data.
Key issues that need to be addressed are as follows:

1) The tie to ALS is extremely weak and somewhat distracting. As another reviewer from the previous round pointed out, the cause of muscle ultrastructure disruption in ALS is impossible to delineate from TEM of patient muscle (indeed, it was also observed in sporadic ALS), thus Figure 1 does not add to the story. If the authors examined muscle from patients with FUS ALS prior to symptom onset and motor neuron degeneration (likely unfeasible), and/or myotubes differentiated from iPSCs of multiple FUS-ALS donors, and/or FusΔNLS/+ mice crossed with a CNS Cre for CNS rescue, the premise that there is cell autonomous muscle dysfunction in FUS ALS would be much stronger. Given that FusΔNLS/+ mice have a profound reduction in hang time at 12 months, TEM of 22 month FusΔNLS/+ muscle seems very late stage and somewhat misleading. The TEM in Fig 2 simply provides evidence that nuclear FUS is necessary for muscle development/differentiation; FusΔNLS/+ mice crossed with a line expressing Cre in post-mitotic muscle would have supported the authors' premise much stronger. The Drosophila model in Fig 3 is more informative as it ablated Fus/caz in post-fusion muscle, but the decrease in muscle number suggests loss of caz causes myocyte death. Hence, the authors need to relegate Fig 1 to supplementary, and if none of the above experiments can be performed, then the results must not be interpreted as evidence of ALS relevant muscle pathology.

We understand this comment by reviewer 3 and appreciate it.

To take it into account, we removed Figure 1 and relegated it to supplementary figures. The manuscript now begins with previous figure 2, showing that muscle ultrastructure is cell autonomously dependent upon FUS expression in development. We toned down the claims on ALS, in both the results and discussion section, shortened the discussion section to refocus on muscle differentiation, rather than maintenance. All changes introduced in the manuscript are highlighted in yellow.

The most significant adaptations are:

- The results section now starts with former figure 2, and results on ALS patients and iPSCs are only cited as Figures EV2 and Appendix Figure 1.
- Our discussion section now begins with the following statement : lines 368-377 : « *Our study identifies a critical function of FUS in muscle development and differentiation through co-activation of key transcription factors involved in muscle differentiation. Specifically, FUS-mediated phase separation of ETV5 and MEF2 promotes the transcription of their target genes. These findings have major implications for our understanding of physiological functions of FUS and could have consequences for FUS-ALS. Our results demonstrate that FUS is critical in muscle development in multiple species. In mice, ablation of Fus or deletion of its nuclear localization sequence results in structural muscle defects including defects in sarcomeric and mitochondrial ultrastructure."*
- The discussion on ALS relevance, that was the first section of the discussion, has been deleted and replaced by this short section later in the discussion: lines 441-446: "*We also observed that mutation of one allele of FUS, either in FUS-ALS patients or in Fus$^{\Delta NLS/+}$ mice, significantly compromised adult muscle ultrastructure. However, our observation of similarly damaged muscle ultrastructure in sporadic ALS patients suggests that these alterations could be either primary or secondary to denervation (Shefner et al., 2023). Future studies should determine the relevance of these developmentally relevant pathways to late-onset muscle weakness and motor neuron degeneration.*

2) The mechanistic studies described in the rest of the manuscript are excellent and provide critical insight into Fus potentiation of MEF2 transcriptional activity. While this work is expertly done, this interpretation should be restructured, as extrapolation to muscle maintenance is poorly supported. All three models used for RNA-seq are differentiation/development models, and defects in differentiation would explain the observed transcriptional changes. As a primary role for Fus in muscle differentiation was clearly supported by the embryonic mouse models, it raises the question of why pathway analysis of Fus ChIP-Seq in C2C12 did not point toward any muscle-specific pathway.

We agree that the interpretation of the RNA-seq results should be more carefully contextualized according to the experimental systems used. All three RNA-seq models employed in this study represent differentiation or developmental contexts, and the observed transcriptional changes could, at

least in part, reflect alterations in differentiation programs rather than direct effects on muscle maintenance per se. We have therefore revised the manuscript to more clearly emphasize this point and to avoid over-extrapolation beyond the supported data. Referring to muscle maintenance has been deleted throughout the manuscript.

Our embryonic mouse data do however support a primary role for FUS in regulating muscle development/differentiation, and we now explicitly align the interpretation of the RNA-seq datasets with this developmental context, including in the title of the manuscript.

Regarding the absence of muscle-specific pathway enrichment in the FUS ChIP-seq data from C2C12 cells, we agree this is an important point. One possible explanation is that FUS occupancy may be highly context-dependent, and muscle-specific regulatory binding events may be more prominent in vivo or at distinct differentiation stages not fully captured in the C2C12 ChIP-seq conditions.

3) It is also unclear from the methods if ChIP-seq was performed on differentiating C2C12 cells or proliferating; the section on C2C12 culture states they were differentiated but the section on chromatin immunoprecipitation does not. If the authors indeed performed ChIP-seq on C2C12 cells in their proliferating myoblast stage, this was an unfortunately major oversight and the experiment should be performed in differentiated cells (or even better, in mature muscle from adult mice) to determine if Fus truly regulates muscle genes in mammals.

We apologize for overlooking to specify the experimental conditions of Chip-seq in the methods. The FUS chip-seq was performed on C2C12 cells differentiated for 2 days (now explicitly stated in line 766 of the revised manuscript).

4) Why was Mrps18c chosen as a Fus-responsive gene instead of a muscle-specific gene (such as one of the genes highlighted in Fig 4f)?
Our choice of Mrps18c as a Fus responsive gene is based on Chip-seq results. To illustrate MEF2 targets, we turned to MEF2-RE luciferase constructs, as illustrated in Figure 3.
This is now explicitly stated in lines 243-245 : « *To determine whether FUS could co-activate genes with ETS transcription factors, we generated a luciferase reporter under the control of the Mrps18c promoter that includes three ETS transcription factor motifs at the FUS binding site (**Fig 4D**).”*

Increase in Mef2 activity is ETV5-dependent, yet the authors state ETV5 is only expressed in subsynaptic nuclei (as is ETV4, and ETV1 does not seem to be expressed in muscle at appreciable levels - https://research.cchmc.org/myoatlas/), thus it is unclear how mutations in Fus could lead to muscle ultrastructural changes outside of development.

We agree with the point that ETV transcription factors are only expressed in embryonic muscles in significant amounts:
Lines 245-247: *“Notably, all three PEA3 ETS transcription factors were expressed at significant levels in embryonic muscle and C2C12 cells (**Appendix Figure 4**)”*

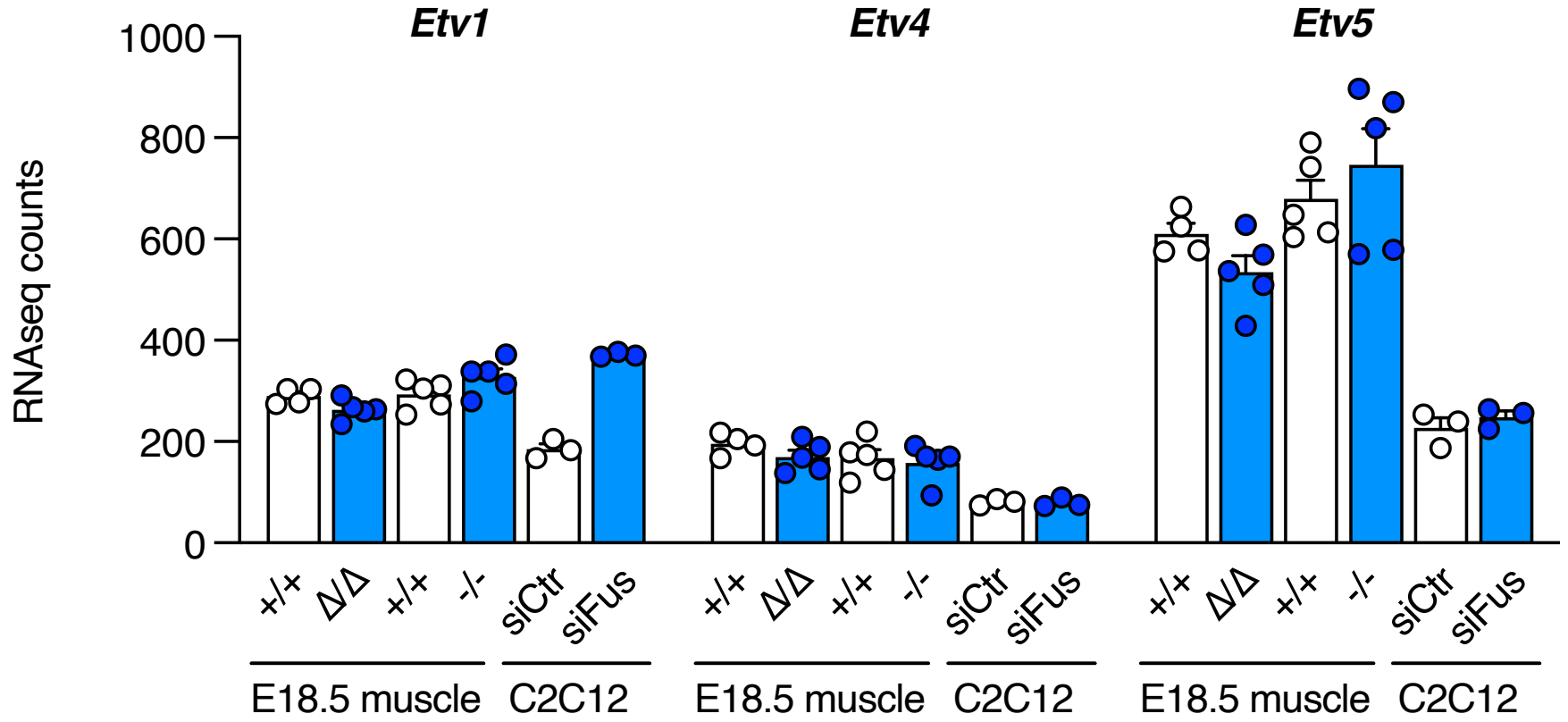


**Appendix Figure 4**

In addition, exploration of datasets from single cell RNAseq studies (Myoatlas (Petrany *et al.*, 2020)) as suggested by the reviewer) shows that ETV4 and ETV5 are significantly expressed in adult subsynaptic nuclei, consistent with their known function in NMJ gene expression. It is therefore possible that the molecular pathways identified here play a functional role in adult muscle, in particular in subsynaptic gene expression, in a manner consistent with our previous study (Picchiarelli *et al.*, 2019).

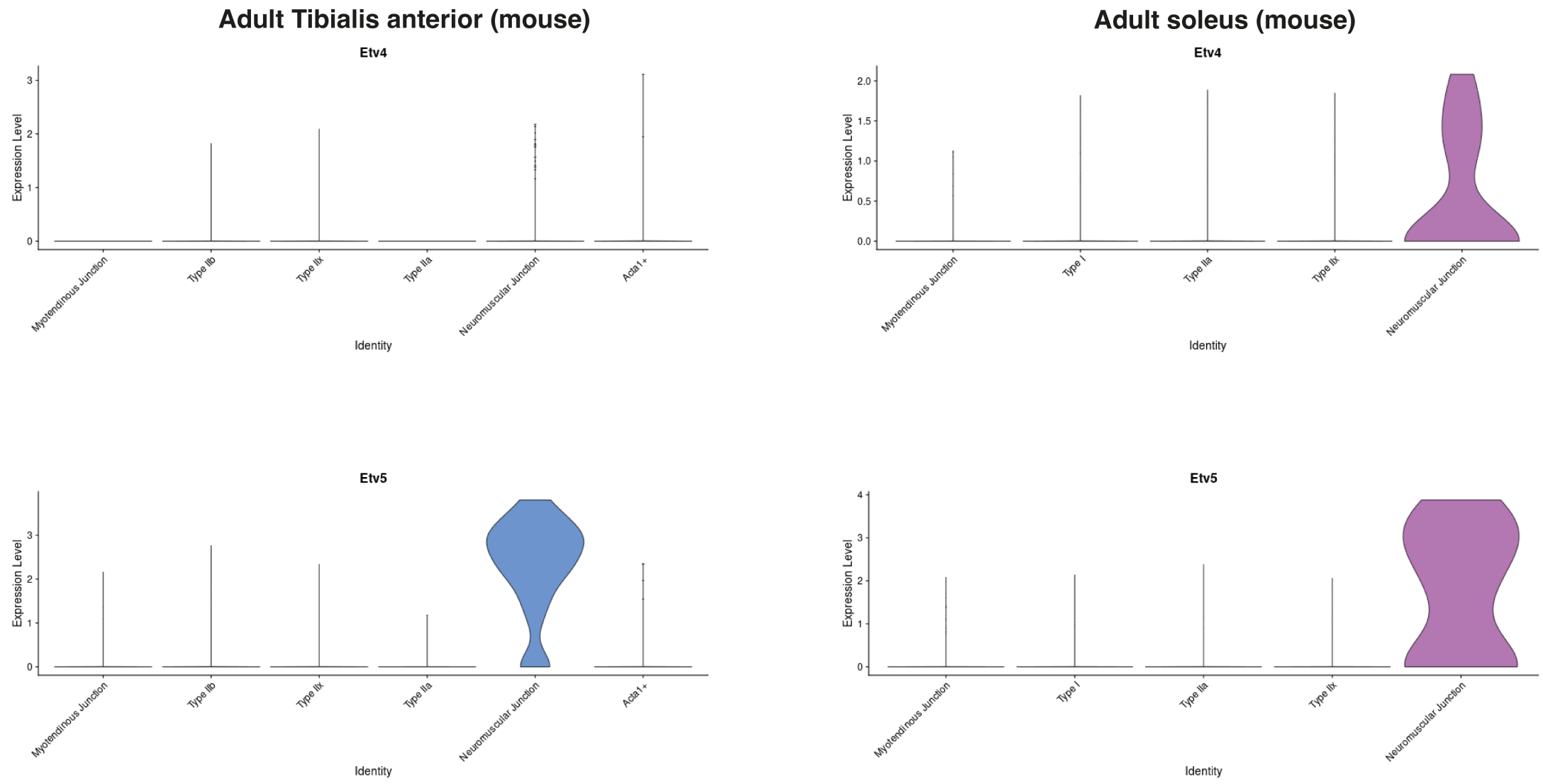


To address the reviewer's comment, we thus focused the manuscript on muscle development.

5) A more thorough characterization of the Fus/Etv5 mouse model presented in Fig 6c is warranted: what was their body weight, muscle weight, muscle fiber cross sectional area, fiber type composition (since mitochondria was repeatedly implicated and discussed), NMJ morphology, spinal cord motor neuron size and number, etc.; there seems to be a lot of informative data that can be gleamed from this model other than simply their hang time.

Beyond the hanging time in the inverted grid test, we had also collected the body weight of the *Fus*$^{\Delta NLS/+}$ x *Etv5*$^{+/-}$ mice, and we have performed electromyography to determine the compound muscle action potential (CMAP). Body weight was evaluated monthly from 16 to 52 weeks of age, and no significant differences were found between the four experimental genotypes at any time point (**Fig EV3A**). The CMAP amplitude was evaluated at 12 and 40 weeks of age, and again no significant differences were found between the genotypes at either age (**Fig EV3B**). These data are included in the revised manuscript in a supplemental figure EV7 and the following sentence was included in the results section: lines 270-273 : « *Interestingly, Etv5 haploinsufficiency did not affect body weight (**Fig EV3A**) or compound muscle action potential amplitude at 12 and 40 weeks of age (**Fig EV3B**)"*

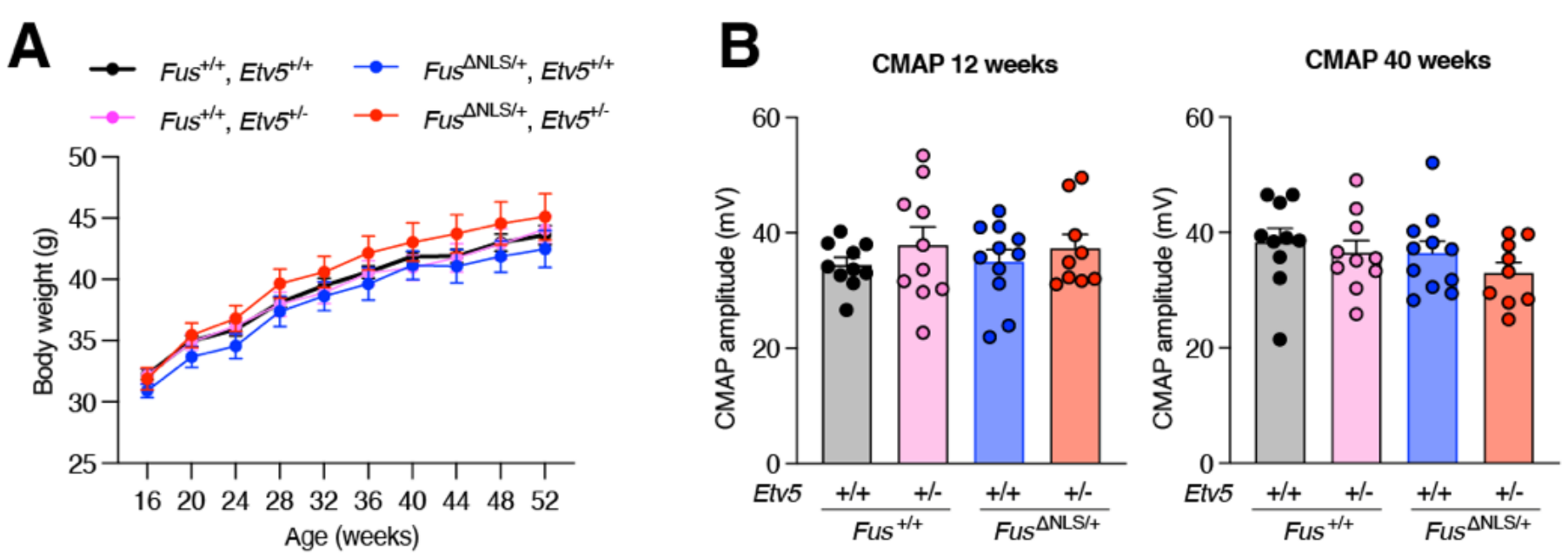


**Figure EV3: Additional characterization of *Etv5* haploinsufficiency in *Fus*$^{\Delta NLS/+}$ mice**

A : Body weight of *Fus*$^{+/+}$; *Etv5*$^{+/+}$, *Fus*$^{+/+}$; *Etv5*$^{+/-}$, *Fus*$^{\Delta NLS/+}$; *Etv5*$^{+/+}$ and *Fus*$^{\Delta NLS/+}$; *Etv5*$^{+/-}$ mice determined every 4 weeks from 16 to 52 weeks of age. n=9-10 mice per genotype; p=not significant by mixed effect analysis with Tukey's multiple comparisons test.
B : Compound muscle action potential (CMAP) amplitude in the gastrocnemius muscle as determined by electromyography (EMG) after stimulation of the sciatic nerve of *Fus*$^{\Delta NLS/+}$ x *Etv5*$^{+/-}$ mice at 12 (left) or 40 (right) weeks of age. n=9-11 mice per genotype; p=not significant by one-way ANOVA.

To collect the inverted grid data shown in the Figure 5C, we have followed 7 cohorts of *Fus*$^{\Delta NLS/+}$ x *Etv5*$^{+/-}$ mice in total. The first cohorts were followed beyond 52 weeks, but because at 52 weeks the *Fus*$^{\Delta NLS/+}$; *Etv5*$^{+/-}$ mice already performed very poorly in the inverted grid test, and they continued to do so beyond 52 weeks of age, we realized that it was not possible to detect further progression of the motor deficit using this test. Therefore, we decided to stop evaluating the motor performance of the subsequent cohorts beyond 52 weeks, but we nevertheless aged the mice until 96 weeks (22 months) of age, because at this age we have previously identified a reduction in motor neuron number in the lumber spinal cord of *Fus*$^{\Delta NLS/+}$ mice, whereas no motor neuron loss was detected at 10 months of age in *Fus*$^{\Delta NLS/+}$ mice (Scekic-Zahirovic *et al*, 2017).

We therefore collected tissues for histological analysis at 96 weeks of age, and determined the weight of the dissected gastrocnemius, extensor digitorum longus (EDL) and soleus muscles. Interestingly, the weight of the gastrocnemius muscle was significantly reduced for *Fus*$^{\Delta NLS/+}$; *Etv5*$^{+/-}$ mice (both as compared to *Fus*$^{+/+}$; *Etv5*$^{+/+}$ and *Fus*$^{\Delta NLS/+}$; *Etv5*$^{+/+}$ mice), but not for any of the other genotypes (Figure 5D). Similarly, the weight of the EDL muscle was significantly reduced for *Fus*$^{\Delta NLS/+}$; *Etv5*$^{+/-}$ mice as compared to *Fus*$^{+/+}$; *Etv5*$^{+/+}$ mice, but not for any of the other genotypes (Figure 5E). In contrast, no differences were detected between genotypes in soleus muscle weight (Figure 5F). Because the soleus muscle is a slow-twitch, oxidative, fatigue-resistant muscle composed primarily of type I (slow-oxidative) and type IIA (fast-oxidative) fibers, this finding is consistent with slow muscle fiber types (and their innervating motor neurons) being spared in ALS (Pun *et al*, 2006). These data are shown in Figure 5D-F in the revised version of the paper and the following sentence was added to the results section: lines 266-270 : « *However, heterozygosity for Etv5 triggered earlier onset and more severe muscle weakness in Fus*$^{\Delta NLS/+}$ *mice (**Fig 5C**), as well as decreased muscle mass in gastrocnemius (**Fig 5D**) and EDL (**Fig 5E**), but not soleus muscle (**Fig 5F**).*

To further address the reviewer's request, we sectioned skeletal muscles and spinal cords of 22-month-old *Fus*$^{\Delta NLS/+}$ x *Etv5*$^{+/-}$ mice and evaluated muscle fiber cross sectional area and motor neuron number. We evaluated the muscle fiber cross sectional area in gastrocnemius muscle, because the weight reduction of this muscle in *Fus*$^{\Delta NLS/+}$; *Etv5*$^{+/-}$ mice is most pronounced. This analysis revealed that the muscle fiber cross sectional area was significantly reduced in *Fus*$^{\Delta NLS/+}$ x *Etv5*$^{+/-}$ mice as compared to *Fus*$^{+/+}$ x *Etv5*$^{+/+}$ mice (Figure 5G-H).
lines 269-270: « *This was accompanied by decreased cross sectional area of muscle fibers in gastrocnemius muscle of Fus*$^{\Delta NLS/+}$ *mice with Etv5 haploinsufficiency (**Fig 5G-H**).*"

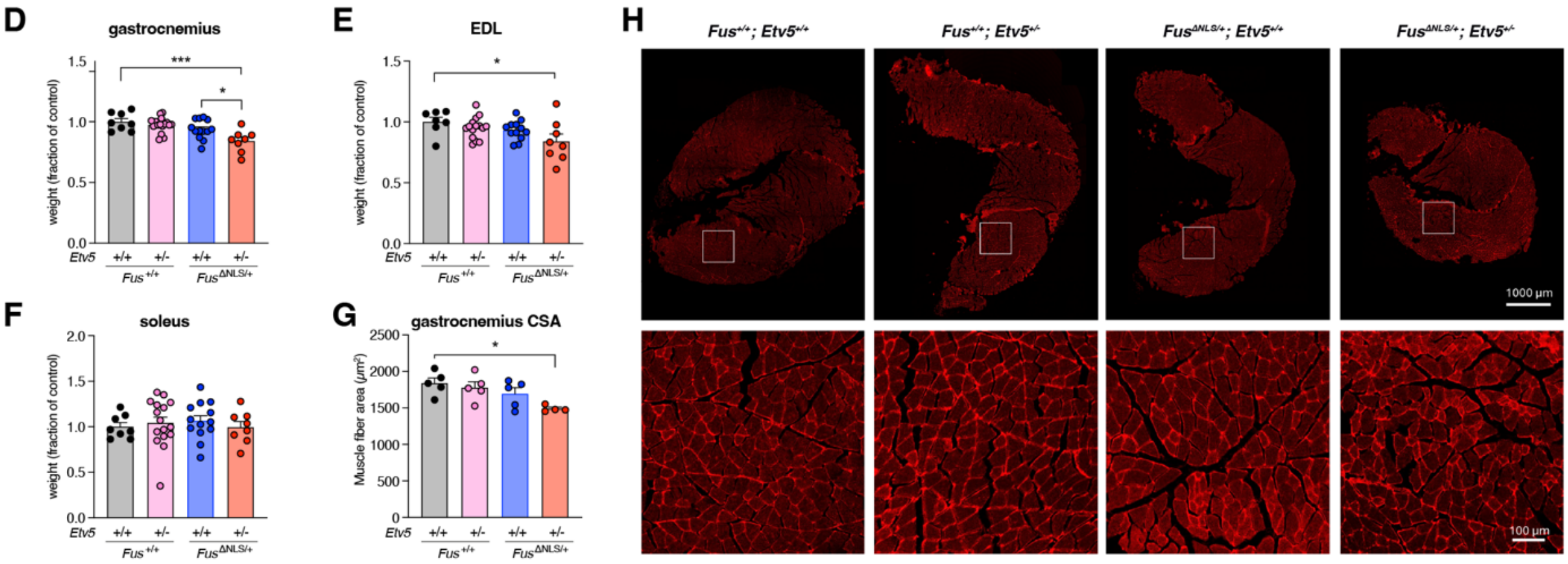

**Figure 5: Reduced expression of *ETV5* orthologues induces muscle defects in *Drosophila* and precipitates muscle deficits of *Fus*$^{\Delta NLS/+}$ mice.**
D-F: Weight of the gastrocnemius (D), extensor digitorum longus (EDL) (E) and soleus (F) muscles of *Fus*$^{\Delta NLS/+}$ x *Etv5*$^{+/-}$ mice at 96 weeks of age, shown as percentage (%) of *Fus*$^{+/+}$; *Etv5*$^{+/+}$. n=7-16 animals per genotype; *p<0.05, ***p<0.001 by one-way ANOVA with Sidak's multiple comparisons test.
G: Average muscle fiber area size in the gastrocnemius muscle of 96-week-old *Fus*$^{\Delta NLS/+}$ x *Etv5*$^{+/-}$ mice. n=4-5 animals per genotype; *p<0.05 by one-way ANOVA with Sidak's multiple comparisons test.
H: Representative images of dystrophin-stained gastrocnemius muscle of the indicated genotypes. Scale bar: 1000$\mu$m (upper row), 100$\mu$m (lower row).

The average number of $\alpha$-motor neurons (defined as ChAT-positive motor neurons with an area size of ≥300 $\mu m^2$) per section in the ventral horn of the lumbar spinal cord was significantly reduced in *Fus*$^{\Delta NLS/+}$; *Etv5*$^{+/+}$ mice at 96 weeks of age, but heterozygosity for *Etv5* did not further reduce the number of $\alpha$-motor neurons (Figure EV3C-D). Thus, while heterozygosity for *Etv5* reduced the muscle fiber cross sectional area in *Fus*$^{\Delta NLS/+}$ mice at 96 weeks of age, which is a proxy for muscle atrophy, it did not affect the progressive motor neuron degeneration in *Fus*$^{\Delta NLS/+}$ mice. This is now stated in lines 271-273 : « *Interestingly, Etv5 haploinsufficiency did not (…) exacerbate late-onset motor neuron loss in Fus*$^{\Delta NLS/+}$ *mice* (***Fig EV3C-D***)."

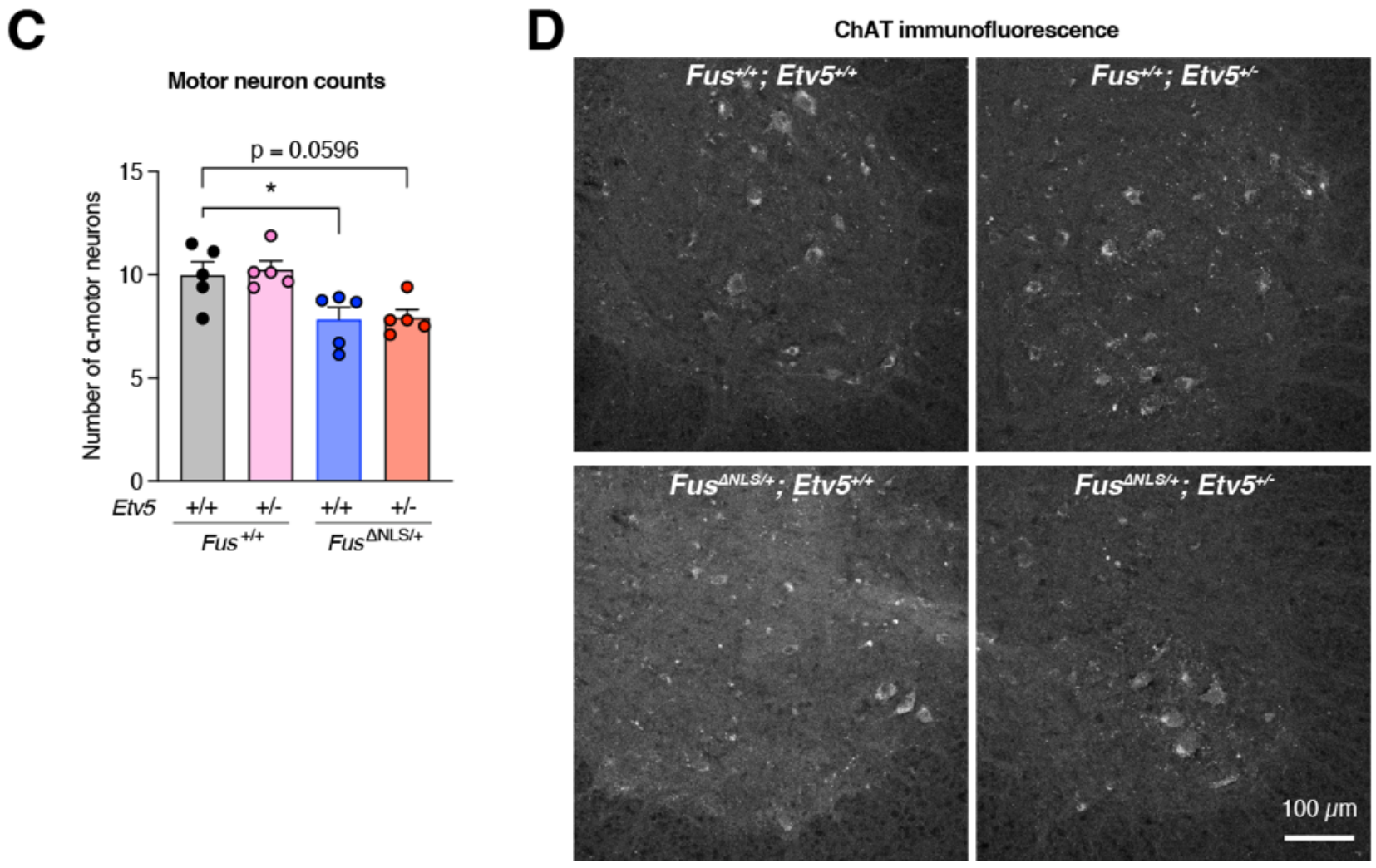


**Figure EV3: Additional characterization of *Etv5* haploinsufficiency in *Fus*$^{\Delta NLS/+}$ mice**
C : Average number of $\alpha$-motor neurons (defined as ChAT-positive motor neurons with an area size of ≥300 $\mu m^2$) per section in the ventral horn of the lumbar spinal cord. n=5 animals per genotype; *p<0.05 by one-way ANOVA with Sidak's multiple comparisons test.
D : Representative ChAT immunohistochemistry images in spinal cord of the indicated genotypes. Scale bar : 100$\mu$m.

Analysis of NMJ morphology and innervation status is very labour intensive and time consuming (4 hours of confocal scanning per muscle needed, plus analysis of 50 NMJs per muscle), and it was therefore not possible to perform this analysis within the allowed 3 months of revision time. Finally, analysis of muscle fiber type distribution was unfortunately not possible, because we only had fixed muscle tissues available, and the antibodies for fiber typing do not work on fixed tissue.

**References:**


Assali A, Harrington AJ, Cowan CW (2019) Emerging roles for MEF2 in brain development and mental disorders. *Curr Opin Neurobiol* 59: 49-58

Banani SF, Lee HO, Hyman AA, Rosen MK (2017) Biomolecular condensates: organizers of cellular biochemistry. *Nat Rev Mol Cell Biol* 18: 285-298

Dalla Torre di Sanguinetto SA, Dasen JS, Arber S (2008) Transcriptional mechanisms controlling motor neuron diversity and connectivity. *Curr Opin Neurobiol* 18: 36-43

Hippenmeyer S, Huber RM, Ladle DR, Murphy K, Arber S (2007) ETS transcription factor Erm controls subsynaptic gene expression in skeletal muscles. *Neuron* 55: 726-740

Huang K, Li J, Ito M, Takeda JI, Ohkawara B, Ogi T, Masuda A, Ohno K (2020) Gene Expression Profile at the Motor Endplate of the Neuromuscular Junction of Fast-Twitch Muscle. *Front Mol Neurosci* 13: 154

Monahan Z, Ryan VH, Janke AM, Burke KA, Rhoads SN, Zerze GH, O'Meally R, Dignon GL, Conicella AE, Zheng W *et al* (2017) Phosphorylation of the FUS low-complexity domain disrupts phase separation, aggregation, and toxicity. *EMBO J* 36: 2951-2967

Patel A, Lee HO, Jawerth L, Maharana S, Jahnel M, Hein MY, Stoynov S, Mahamid J, Saha S, Franzmann TM *et al* (2015) A Liquid-to-Solid Phase Transition of the ALS Protein FUS Accelerated by Disease Mutation. *Cell* 162: 1066-1077

Petrany MJ, Swoboda CO, Sun C, Chetal K, Chen X, Weirauch MT, Salomonis N, Millay DP (2020) Single-nucleus RNA-seq identifies transcriptional heterogeneity in multinucleated skeletal myofibers. *Nat Commun* 11: 6374

Picchiarelli G, Demestre M, Zuko A, Been M, Higelin J, Dieterle S, Goy MA, Mallik M, Sellier C, Scekic-Zahirovic J *et al* (2019) FUS-mediated regulation of acetylcholine receptor transcription at neuromuscular junctions is compromised in amyotrophic lateral sclerosis. *Nat Neurosci*

Pun S, Santos AF, Saxena S, Xu L, Caroni P (2006) Selective vulnerability and pruning of phasic motoneuron axons in motoneuron disease alleviated by CNTF. *Nat Neurosci* 9: 408-419

Qamar S, Wang G, Randle SJ, Ruggeri FS, Varela JA, Lin JQ, Phillips EC, Miyashita A, Williams D, Strohl F *et al* (2018) FUS Phase Separation Is Modulated by a Molecular Chaperone and Methylation of Arginine Cation-pi Interactions. *Cell* 173: 720-734 e715

Reber S, Jutzi D, Lindsay H, Devoy A, Mechtersheimer J, Levone BR, Domanski M, Bentmann E, Dormann D, Muhlemann O *et al* (2021) The phase separation-dependent FUS interactome reveals nuclear and cytoplasmic function of liquid-liquid phase separation. *Nucleic Acids Res* 49: 7713-7731

Scekic-Zahirovic J, Oussini HE, Mersmann S, Drenner K, Wagner M, Sun Y, Allmeroth K, Dieterle S, Sinniger J, Dirrig-Grosch S *et al* (2017) Motor neuron intrinsic and extrinsic mechanisms contribute to the pathogenesis of FUS-associated amyotrophic lateral sclerosis. *Acta Neuropathol* 133: 887-906

Shefner JM, Musaro A, Ngo ST, Lunetta C, Steyn FJ, Robitaille R, De Carvalho M, Rutkove S, Ludolph AC, Dupuis L (2023) Skeletal muscle in amyotrophic lateral sclerosis. *Brain* 146: 4425-4436

## 1st Revision - Editorial Decision 22nd Jun 2026

Dear Luc,

Thanks again for the submission of your revised manuscript (EMBOJ-2025-122972R) to The EMBO Journal for our consideration, and for your patience during re-review. Your manuscript and your point-by-point responses to the previous comments of the referees have now been seen by them, and we have received their new reports, which you can find appended below. I would also like to thank you for your feedback on the remaining point of referee #2, which I have already shared with you.

I am pleased to say that the referees are now, overall, largely supportive. Referees #1 and #3 mention that the initially raised concerns have now been adequately addressed, and referee #2 also finds the conceptual advance and mechanistic insights provided by the revised manuscript substantial. This referee makes one remaining point regarding the previously requested validation of the mitochondrial protein targets of MEF2A and ETV5. The referee recommends further experiments to investigate the endogenous expression levels and co-localization of FUS and its binding partners in appropriate genetic backgrounds. Your response to this comment was that you are currently unable to perform these experiments at endogenous expression levels, as there are no available antibodies validated for super-resolution analysis for endogenous FUS, MEF2A, and ETV5. Taking this input into consideration, our decision is that we will not request such experiments for the publication of this manuscript, but we kindly request that this limitation be transparently discussed in a final version of the manuscript, as well as in a point-by-point response to the referees' comments. In addition, there are two suggestions for minor improvements in the text offered by referee #3 (please see below), which should also be addressed in the final revision and point-by-point response.

From the editorial side, there are also a few changes and corrections we need you to make in the final version of the manuscript, before we can move forward with the next steps towards its publication:

- We noticed that four co-authors are marked as co-corresponding authors of the manuscript, which is unusual for our journal. We kindly request that you review carefully again the contributions of each co-author and consider limiting the corresponding author status to no more than three co-authors. Please also note that we use the CRediT author contributions taxonomy system for specifying the contributions of each co-author, and detailed descriptions can also be provided if deemed useful (please see also below for more information).

- Please make sure that an ORCID identifier is linked to the profile in our system of each co-corresponding author of the final version of the manuscript (no ORCID ID is currently linked to the profile of co-author Chantal Sellier); an institutional e-mail address must also be provided on the title page of the manuscript for each one of them.

- Please note that funding information currently provided in the Comments box in our online system must be entered using the "More Funders" list, so that it can be properly extracted by our production team. This information must be identical to the funding information provided in the Acknowledgements section of the manuscript.

- Please list in the Data Availability section of your revised manuscript the database, permanent identifier, and specific URL for each deposited dataset (in particular, for all sequencing datasets generated in this study). Please also make sure that all data will be publicly available at the time of publication. The reviewer token is no longer necessary and can now be removed from the Data Availability section.

- The Author Contributions statement should be removed from the manuscript file. Instead, we use CRediT to specify the contributions of each author in the journal submission system. Please feel free to use the free text box to provide more detailed descriptions during submission. See also our guide to authors for more information: https://link.springer.com/partners/embo-press/editorial-policies#Authorship.

- We noticed that callouts in the main text for panels of EV and Appendix Figures are missing. Please make sure that each panel of each EV and Appendix Figure is called out, at least once, in the main text, as appropriate.

- EV Figures should be uploaded to our online system as individual, high-resolution Figure files with their legends listed below those of the regular Figures, in the main manuscript file.

- The title page of the Appendix PDF file should contain heading "Appendix for", followed by the manuscript's title (author and affiliation lists are not necessary) and a Table of Contents including page numbers for all listed items; nomenclature should be "Appendix Figure S#" and "Appendix Table S#" throughout the manuscript and the Appendix file.

- Please move the References that are currently listed in your Reagents and Tools Table file to the main list of References in the manuscript.

- Please note that EMBO press papers are accompanied online by:
A) a short (2 sentences) summary of the findings and their significance,

B) 2-5 short bullet points highlighting the key results, and
C) a synopsis image in .jpg or .png format that is exactly 550 pixels wide and 300 pixels high. Please note that all text needs to be legible at the final size.
Please upload this information along with your revised manuscript (the text for A and B should be provided in a separate Word file).

- Our data editors have checked your Figures and their legends, and raised the following queries. Please address all points below completely in your revised manuscript (all changes should be highlighted or "tracked"):
1. Please define the annotated p-values ****/** as well as provide the exact p-values for the same in the legend of Figure 7B, as appropriate.
2. Please provide the exact p-values in the legends of Figures 1E-G; 2C-F; 3F,I,J,K; 4H-K; 5B-G; 6C-E; 8B-E,H-K; EV-1B-E; EV-2C,D,E; EV-3C.
3. Please define the error bars in the legends of Figures 5B-G; EV-1B-E; EV-2A,C,D,E; EV-3A,B,C.

- The manuscript sections need to be named and ordered as follows: Title page - Abstract - Introduction - Results - Discussion - Methods - Data Availability - Acknowledgements - Disclosure and Competing Interests Statement - References - Figure Legends - main Tables (if there are any) - Expanded View Figure Legends (if there are any).

- Please also note that as part of the EMBO Press transparent editorial process, The EMBO Journal publishes online a Peer Review File along with each accepted manuscript. This File will be published in conjunction with your paper and will include the referee reports, your point-by-point responses and all pertinent correspondence relating to the manuscript. Your Author's Checklist will also be published at the end of the Peer Review File. Please let us know in case you want to remove any data or figures from your point-by-point responses before they are published as part of the Peer Review File. Retaining unpublished data in the Peer Review File means that these count as published and that the Peer Review File would need to be referenced in future publications. Please let the editorial office know in case you want to remove any data from this file (contact@embojournal.org).

I would like to thank you for informing us that you are interested in preparing an image/illustration for the front cover of our journal once a final decision has been made on the manuscript. We would be very happy to consider your candidate image(s)/illustration(s). Please feel free to contact my colleague Cornelius Schneider (c.schneider@embojournal.org), who currently coordinates the front cover pipeline for our journal, for more information.

We look forward to seeing a final version of your manuscript as soon as possible. Please let us know if you have any questions and use this link to submit your revision: https://emboj.msubmit.net/cgi-bin/main.plex.

Best regards,

Ioannis

Ioannis Papaioannou, PhD
Editor, The EMBO Journal
i.papaioannou@embojournal.org

------------------------------------------------

Referee #1:

The authors have sufficiently addressed my additional comments (and as far as I can see those of other reviewers).

Referee #2:

FUS is an RNA-binding protein, and mutations in its gene cause early-onset ALS, characterized by motor neuron degeneration and progressive muscle atrophy with rapid progression. However, whether ALS disease mutations expressed in muscle lead to loss of function or gain of toxic function remains to be further investigated. This revised manuscript utilized FUS KO and ΔNLS

mutant mouse models, the Drosophila model, and iPSC-derived myotubes to reveal that both FUS knockout and mutations result in muscle developmental defects and abnormal mitochondrial morphology. Through RNA-seq of E18.5 embryonic cells from FUS KO and mutant mice, as well as C2C12 cells after siFUS treatment, the authors found that the RNA levels of a series of muscle mitochondria-related proteins were downregulated. They identified transcription factors MEF2 and ETV from the perspective of transcription factors and their binding sites, revealing that FUS participates in the regulation of the transcription of muscle development-related mitochondrial proteins. Mechanistically, they demonstrate that FUS recruits MEF2A and ETV5 via LLPS and activates the transcription of their target genes, while ALS-linked mutations cause the loss of this regulatory function.

Overall, this study reveals the role of FUS in muscle development and maintenance of muscle structure, and provides compelling mechanistic insights into how FUS mutations precipitate muscle pathology in ALS.

I still have one big question about the authors' responses regarding my first major concern about the mitochondrial protein targets of MEF2A and ETV5. The authors posted a manuscript for consideration elsewhere, which highlights metabolic dysfunction by FUS deficiency and possible molecular players downstream of MEF2A and ETV5. However, as I mentioned in the previous review, the study lacks protein-level validation of FUS, MEF2A, and ETV5 in FUS KO and mutant mice, iPSC, and siFUS-treated C2C12 cells. In Fig 8A, the authors used purified protein to examine the LLPS of FUS in the absence or the presence of ETV5 and/or MEF2A. In Fig 8F and G, along with Appendix Figs 3 and 9, HEK293 cells were transfected with Myc-tagged variants of FUS and HA-tagged variants of either ETV5 or MEF2A, and the expression level of FUS and the localization of three proteins were examined. These data are all from exogenous expression of FUS. Therefore, the endogenous expression levels and co-localization of FUS and its binding partners, MEF2A and ETV5, in FUS KO and mutant mice, and siFUS-treated C2C12 cells, such as WB and immunofluorescence/immune-EM, are required.

Referee #3:

All of my concerns were excellently addressed, and I really appreciate tuning the presentation of these data to muscle development while somewhat sidelining the ALS aspect. The authors should be commended on the large amount of revisions completed in a timely manner. Future studies examining whether this FUS-MEF2-ETV5 mechanism is involved in maintaining subsynaptic muscle nuclei (and perhaps if FUS mutations affect muscle-nerve communication building on the authors' previous 2019 findings) would be of great interest, as the authors alluded to in their response.

Minor comments:

1) Line 427 "..that was disrupted when LLPS-deficient FUS was expressed (Fig EV5)." Perhaps citing Fig 8 where the FUS mutants are used instead of the schematic would be more accurate.

2) A brief comment in the Discussion on why FusΔNLS/+ Etv5+/- mice do not have progressively reduced body weight (and thus, progressive muscle atrophy) or CMAP deficiencies may be warranted. From the inverted grid test, it seems like they may have a deficit in muscle endurance, not so much strength; if appropriate, a comment on this would tie in nicely with the other metabolic manuscript the authors are submitting.

**Referee #1:**

The authors have sufficiently addressed my additional comments (and as far as I can see those of other reviewers).

We thank referee 1 for their positive review of our work and constructive suggestions throughout the review process.

**Referee #2:**

FUS is an RNA-binding protein, and mutations in its gene cause early-onset ALS, characterized by motor neuron degeneration and progressive muscle atrophy with rapid progression. However, whether ALS disease mutations expressed in muscle lead to loss of function or gain of toxic function remains to be further investigated. This revised manuscript utilized FUS KO and ΔNLS mutant mouse models, the Drosophila model, and iPSC-derived myotubes to reveal that both FUS knockout and mutations result in muscle developmental defects and abnormal mitochondrial morphology. Through RNA-seq of E18.5 embryonic cells from FUS KO and mutant mice, as well as C2C12 cells after siFUS treatment, the authors found that the RNA levels of a series of muscle mitochondria-related proteins were downregulated. They identified transcription factors MEF2 and ETV from the perspective of transcription factors and their binding sites, revealing that FUS participates in the regulation of the transcription of muscle development-related mitochondrial proteins. Mechanistically, they demonstrate that FUS recruits MEF2A and ETV5 via LLPS and activates the transcription of their target genes, while ALS-linked mutations cause the loss of this regulatory function.

Overall, this study reveals the role of FUS in muscle development and maintenance of muscle structure, and provides compelling mechanistic insights into how FUS mutations precipitate muscle pathology in ALS.

We thank referee 2 for their positive review of our work.

I still have one big question about the authors' responses regarding my first major concern about the mitochondrial protein targets of MEF2A and ETV5. The authors posted a manuscript for consideration elsewhere, which highlights metabolic dysfunction by FUS deficiency and possible molecular players downstream of MEF2A and ETV5. However, as I mentioned in the previous review, the study lacks protein-level validation of FUS, MEF2A, and ETV5 in FUS KO and mutant mice, iPSC, and siFUS-treated C2C12 cells. In Fig 8A, the authors used purified protein to examine the LLPS of FUS in the absence or the presence of ETV5 and/or MEF2A. In Fig 8F and G, along with Appendix Figs 3 and 9, HEK293 cells were transfected with Myc-tagged variants of FUS and HA-tagged variants of either ETV5 or MEF2A, and the expression level of FUS and the localization of three proteins were examined. These data are all from exogenous expression of FUS. Therefore, the endogenous expression levels and co-localization of FUS and its binding partners, MEF2A and ETV5, in FUS KO and mutant mice, and siFUS-treated C2C12 cells, such as WB and immunofluorescence/immune-EM, are required.

We acknowledge that super-resolution imaging was performed in overepression models, and that detection of MEF2A and ETV5 was mostly performed using tagged epitopes. Unfortunately, we were not able to obtain commercial antibodies for ETV5 and MEF2A leading to reliable signals in endogenous cell extracts (although the antibodies were working on purified proteins as illustrated in Fig 7A). This precluded us to perform either co-IPs or super-resolution imaging of endogenous MEF2 and ETV5.

To acknowledge this limitation, we state in the discussion (lines 433-436) : « *It is important to note that our results were mostly obtained upon overexpression of FUS, MEF2A and ETV5, and that it will be critical to demonstrate these interactions at native endogenous expression levels."*

**Referee #3:**

All of my concerns were excellently addressed, and I really appreciate tuning the presentation of these data to muscle development while somewhat sidelining the ALS aspect. The authors should be commended on the large amount of revisions completed in a timely manner. Future studies examining whether this FUS-MEF2-ETV5 mechanism is involved in maintaining subsynaptic muscle nuclei (and perhaps if FUS mutations affect muscle-nerve communication building on the authors' previous 2019 findings) would be of great interest, as the authors alluded to in their response.

We thank referee 3 for their positive review of our work. We particularly appreciated the suggestion to focus on muscle development which led to a more focused manuscript.

Minor comments:

1) Line 427 "..that was disrupted when LLPS-deficient FUS was expressed (Fig EV5)." Perhaps citing Fig 8 where the FUS mutants are used instead of the schematic would be more accurate.

This has been done.

2) A brief comment in the Discussion on why FusΔNLS/+ Etv5+/- mice do not have progressively reduced body weight (and thus, progressive muscle atrophy) or CMAP deficiencies may be warranted. From the inverted grid test, it seems like they may have a deficit in muscle endurance, not so much strength; if appropriate, a comment on this would tie in nicely with the other metabolic manuscript the authors are submitting.

We thank the reviewer for this suggestion, and have included one sentence in the discussion as such (line 442) : « This restricted pattern of expression in adult muscle might explain why haploinsufficiency of *Etv5* in *Fus*$^{\Delta NLS/+}$ mice leads to defect in muscle endurance rather than muscle strength."

## 2nd Revision - Editorial Decision 26th Jun 2026

Dear Luc,

Congratulations on an excellent manuscript! I am very pleased to inform you that it has been accepted for publication in The EMBO Journal. Thank you for comprehensively addressing the initially raised referees' concerns and our editorial requests for changes and corrections.

Your manuscript will now be processed for publication by EMBO Press. Please note that we will only be able to proceed with the next steps towards the publication of your article once ORCID IDs have been correctly linked to the profiles of all co-corresponding authors in our system. Our team has already sent you a separate message with instructions on how to complete the ORCID ID linking.

Your manuscript will then be copy edited and you will receive page proofs prior to publication. Please note that you will be contacted by Springer Nature Author Services to complete licensing and payment information.

You may qualify for financial assistance for your publication charges - either via a Springer Nature fully open access agreement or an EMBO initiative. Check your eligibility: https://link.springer.com/journal/44318/how-to-publish-with-us

Should you be planning a Press Release on your article, please notify the production team via this form in order to coordinate publication and release dates: https://forms.cloud.microsoft/e/eH4A8JVP6g

If you have any questions, please do not hesitate to contact the Editorial Office. Thank you for your contribution to The EMBO Journal. Working with you has been a pleasure.

Best regards,

Ioannis

Ioannis Papaioannou, PhD
Editor, The EMBO Journal
i.papaioannou@embojournal.org

------------------------------------------------

*****
Please note that it is The EMBO Journal policy for the transcript of the editorial process (containing referee reports and your response letters) to be published as an online supplement to each paper. If you should prefer removal of any referee-only figures included in the point-by-point response(s), e.g. because they may still be used for future publication or because they have been reproduced from published work by others, please do let us know immediately via response email.
More information is available here: https://link.springer.com/partners/embo-press/editorial-policies#Peer%20review

** Click here to be directed to your login page: https://emboj.msubmit.net

## EMBO Press Author Checklist

| | |
|---|---|
| Corresponding Author Name: DUPUIS | |
| Journal Submitted to: The EMBO Journal | |
| Manuscript Number: EMBOJ-2025-122972R | |

**USEFUL LINKS FOR COMPLETING THIS FORM**
The EMBO Journal - Author Guidelines
EMBO Reports - Author Guidelines
Molecular Systems Biology - Author Guidelines
EMBO Molecular Medicine - Author Guidelines

**Reporting Checklist for Life Science Articles (updated January**
This checklist is adapted from Materials Design Analysis Reporting (MDAR) Checklist for Authors. MDAR establishes a minimum set of requirements in transparent reporting in the life sciences (see Statement of Task: 10.31222/osf.io/9sm4x). Please follow the journal's guidelines in preparing your
**Please note that a copy of this checklist will be published alongside your article.**

**Abridged guidelines for figures**
**1. Data**
The data shown in figures should satisfy the following conditions:
- the data were obtained and processed according to the field's best practice and are presented to reflect the results of the experiments in an accurate and unbiased manner.
- ideally, figure panels should include only measurements that are directly comparable to each other and obtained with the same assay.
- plots include clearly labeled error bars for independent experiments and sample sizes. Unless justified, error bars should not be shown for technical
- if n<5, the individual data points from each experiment should be plotted. Any statistical test employed should be justified.
- Source Data should be included to report the data underlying figures according to the guidelines set out in the authorship guidelines on Data

**2. Captions**
Each figure caption should contain the following information, for each panel where they are relevant:
- a specification of the experimental system investigated (eg cell line, species name).
- the assay(s) and method(s) used to carry out the reported observations and measurements.
- an explicit mention of the biological and chemical entity(ies) that are being measured.
- an explicit mention of the biological and chemical entity(ies) that are altered/varied/perturbed in a controlled manner.
- the exact sample size (n) for each experimental group/condition, given as a number, not a range;
- a description of the sample collection allowing the reader to understand whether the samples represent technical or biological replicates (including how many animals, litters, cultures, etc.).
- a statement of how many times the experiment shown was independently replicated in the laboratory.
- definitions of statistical methods and measures:
  - common tests, such as t-test (please specify whether paired vs. unpaired), simple χ2 tests, Wilcoxon and Mann-Whitney tests, can be unambiguously identified by name only, but more complex techniques should be described in the methods section;
  - are tests one-sided or two-sided?
  - are there adjustments for multiple comparisons?
  - exact statistical test results, e.g., P values = x but not P values < x;
  - definition of 'center values' as median or average;
  - definition of error bars as s.d. or s.e.m.

**Please complete ALL of the questions below.**
**Select "Not Applicable" only when the requested information is not relevant for your study.**

**Materials**

| Newly Created Materials | Information included in the manuscript? | In which section is the information available? (Reagents and Tools Table, Materials and Methods, Figures, Data Availability Section) |
|---|---|---|
| New materials and reagents need to be available; do any restrictions apply? | Not Applicable | |

| Antibodies | Information included in the manuscript? | In which section is the information available? (Reagents and Tools Table, Materials and Methods, Figures, Data Availability Section) |
|---|---|---|
| For **antibodies** provide the following information:<br>- Commercial antibodies: RRID (if possible) or supplier name, catalogue number and or/clone number<br>- Non-commercial: RRID or citation | Yes | Reagents and Tools tables, Materials and Methods |

| DNA and RNA sequences | Information included in the manuscript? | In which section is the information available? (Reagents and Tools Table, Materials and Methods, Figures, Data Availability Section) |
|---|---|---|
| **Short novel DNA or RNA including primers, probes**: provide the sequences. | Yes | Materials and methods |

| Cell materials | Information included in the manuscript? | In which section is the information available? (Reagents and Tools Table, Materials and Methods, Figures, Data Availability Section) |
|---|---|---|
| **Cell lines:** Provide species information, strain. Provide accession number in repository **OR** supplier name, catalog number, clone number, and/**OR** RRID. | Yes | Reagents and Tools tables, Materials and Methods |
| **Primary cultures:** Provide species, strain, sex of origin, genetic modification status. | Not Applicable | |
| Report if the cell lines were recently **authenticated** (e.g., by STR profiling) and tested for mycoplasma contamination. | Yes | Reagents and Tools tables, Materials and Methods |

| Experimental animals | Information included in the manuscript? | In which section is the information available? (Reagents and Tools Table, Materials and Methods, Figures, Data Availability Section) |
|---|---|---|
| **Laboratory animals or Model organisms:** Provide species, strain, sex, age, genetic modification status. Provide accession number in repository **OR** supplier name, catalog number, clone number, **OR** RRID. | Yes | Reagents and Tools tables, Materials and Methods |
| **Animal observed in or captured from the field:** Provide species, sex, and age where possible. | Not Applicable | |
| Please detail **housing and husbandry conditions**. | Yes | Materials and methods |

| Plants and microbes | Information included in the manuscript? | In which section is the information available? (Reagents and Tools Table, Materials and Methods, Figures, Data Availability Section) |
|---|---|---|
| **Plants:** provide species and strain, ecotype and cultivar where relevant, unique accession number if available, and source (including location for collected wild specimens). | Not Applicable | |
| **Microbes:** provide species and strain, unique accession number if available, and source. | Not Applicable | |

| Human research participants | Information included in the manuscript? | In which section is the information available? (Reagents and Tools Table, Materials and Methods, Figures, Data Availability Section) |
|---|---|---|
| If collected and within the bounds of privacy constraints report on age, sex and gender or ethnicity for all study participants. | Yes | Materials and methods |

| Core facilities | Information included in the manuscript? | In which section is the information available? (Reagents and Tools Table, Materials and Methods, Figures, Data Availability Section) |
|---|---|---|
| If your work benefited from core facilities, was their service mentioned in the acknowledgments section? | Yes | Acknowledgments |

**Design**

| Study protocol | Information included in the manuscript? | In which section is the information available? (Reagents and Tools Table, Materials and Methods, Figures, Data Availability Section) |
|---|---|---|
| If study protocol has been **pre-registered, provide DOI in the manuscript**. For clinical trials, provide the trial registration number **OR** cite DOI. | Not Applicable | |
| Report the **clinical trial registration number** (at ClinicalTrials.gov or equivalent), where applicable. | Not Applicable | |

| Laboratory protocol | Information included in the manuscript? | In which section is the information available? (Reagents and Tools Table, Materials and Methods, Figures, Data Availability Section) |
|---|---|---|
| Provide DOI OR other citation details if **external detailed step-by-step protocols** are available. | Not Applicable | |

| Experimental study design and statistics | Information included in the manuscript? | In which section is the information available? (Reagents and Tools Table, Materials and Methods, Figures, Data Availability Section) |
|---|---|---|
| Include a statement about **sample size** estimate even if no statistical methods were used. | Yes | Materials and methods |
| Were any steps taken to minimize the effects of subjective bias when allocating animals/samples to treatment (e.g. **randomization procedure**)? If yes, have they been described? | Not Applicable | |
| Include a statement about **blinding** even if no blinding was done. | Yes | Materials and methods |
| Describe **inclusion/exclusion criteria** if samples or animals were excluded from the analysis. Were the criteria pre-established?<br><br>If sample or data points were omitted from analysis, report if this was due to attrition or intentional exclusion and provide justification. | Not Applicable | |
| For every figure, are **statistical tests** justified as appropriate? Do the data meet the assumptions of the tests (e.g., normal distribution)? Describe any methods used to assess it. Is there an estimate of variation within each group of data? Is the variance similar between the groups that are being statistically compared? | Yes | Materials and methods |

| Sample definition and in-laboratory replication | Information included in the manuscript? | In which section is the information available? (Reagents and Tools Table, Materials and Methods, Figures, Data Availability Section) |
|---|---|---|
| In the figure legends: state number of times the experiment was **replicated** in laboratory. | Yes | Materials and methods, figure legends |
| In the figure legends: define whether data describe **technical or biological replicates**. | Yes | Materials and methods, figure legends |

## Ethics

| Ethics | Information included in the manuscript? | In which section is the information available? (Reagents and Tools Table, Materials and Methods, Figures, Data Availability Section) |
|---|---|---|
| Studies involving **human participants**: State details of **authority granting ethics approval** (IRB or equivalent committee(s), provide reference number for approval. | Yes | Materials and methods |
| Studies involving **human participants**: Include a statement confirming that **informed consent** was obtained from all subjects and that the experiments conformed to the principles set out in the WMA Declaration of Helsinki and the Department of Health and Human Services Belmont Report. | Yes | Materials and methods |
| Studies involving **human participants:** For publication of **patient photos**, include a statement confirming that consent to publish was obtained. | Not Applicable | |
| Studies involving experimental **animals**: State details of **authority granting ethics approval** (IRB or equivalent committee(s), provide reference number for approval. Include a statement of compliance with ethical regulations. | Yes | Materials and methods |
| Studies involving **specimen and field samples:** State if relevant **permits** obtained, provide details of authority approving study; if none were required, explain why. | Not Applicable | |

| Dual Use Research of Concern (DURC) | Information included in the manuscript? | In which section is the information available? (Reagents and Tools Table, Materials and Methods, Figures, Data Availability Section) |
|---|---|---|
| Could your study fall under dual use research restrictions? Please check biosecurity documents and list of **select agents and toxins** (CDC): https://www.selectagents.gov/sat/list.htm | Not Applicable | |
| If you used a select agent, is the security level of the lab appropriate and reported in the manuscript? | Not Applicable | |
| If a study is subject to dual use research of concern regulations, is the name of the **authority granting approval and reference number** for the regulatory approval provided in the manuscript? | Not Applicable | |

## Reporting

The MDAR framework recommends adoption of discipline-specific guidelines, established and endorsed through community initiatives. Journals have their own policy about requiring specific guidelines and recommendations to complement MDAR.

| Adherence to community standards | Information included in the manuscript? | In which section is the information available? (Reagents and Tools Table, Materials and Methods, Figures, Data Availability Section) |
|---|---|---|
| State if relevant guidelines or checklists (e.g., **ICMJE, MIBBI, ARRIVE, PRISMA**) have been followed or provided. | Not Applicable | |
| For **tumor marker prognostic studies,** we recommend that you follow the **REMARK** reporting guidelines (see link list at top right). See author guidelines, under 'Reporting Guidelines'. Please confirm you have followed these guidelines. | Not Applicable | |
| For **phase II and III randomized controlled trials**, please refer to the **CONSORT** flow diagram (see link list at top right) and submit the CONSORT checklist (see link list at top right) with your submission. See author guidelines, under 'Reporting Guidelines'. Please confirm you have submitted this list. | Not Applicable | |

## Data Availability

| Data availability | Information included in the manuscript? | In which section is the information available? (Reagents and Tools Table, Materials and Methods, Figures, Data Availability Section) |
|---|---|---|
| Have **primary datasets** been deposited according to the journal's guidelines (see 'Data Deposition' section) and the respective accession numbers provided in the Data Availability Section? | Yes | Data availability |
| Were **human clinical and genomic datasets** deposited in a public access-controlled repository in accordance to ethical obligations to the patients and to the applicable consent agreement? | Not Applicable | |
| Are **computational models** that are central and integral to a study available without restrictions in a machine-readable form? Were the relevant accession numbers or links provided? | Not Applicable | |
| If publicly available data were reused, provide the respective **data citations in the reference list**. | Yes | Materials and methods |
